# Site-specific amino acid substitution in dodecameric peptides determines the stability and unfolding of *c-MYC* quadruplex promoting apoptosis in cancer cells

**DOI:** 10.1093/nar/gky824

**Published:** 2018-09-17

**Authors:** Pallabi Sengupta, Nilanjan Banerjee, Tanaya Roychowdhury, Anindya Dutta, Samit Chattopadhyay, Subhrangsu Chatterjee

**Affiliations:** 1 Department of Biophysics, Bose Institute, P-1/12 CIT Scheme VII (M), Kolkata 700054, India; 2 Cancer Biology and Inflammatory Disorder Division, CSIR-Indian Institute of Chemical Biology, 4, Raja S.C. Mullick Road, Kolkata 700032, India

## Abstract

*c-MYC* proto-oncogene harbours a transcription-inhibitory quadruplex-forming scaffold (Pu27) upstream P_1_ promoter providing anti-neoplastic therapeutic target. Previous reports showed the binding profile of human Cathelicidin peptide (LL37) and telomeric G-quadruplex. Here, we truncated the quadruplex-binding domain of LL37 to prepare a small library of peptides through site-specific amino acid substitution. We investigated the intracellular selectivity of peptides for Pu27 over other oncogenic quadruplexes and their role in *c-MYC* promoter repression by dual-luciferase assays. We analysed their thermodynamics of binding reactions with *c-MYC* quadruplex isomers (Pu27, Myc22, Pu19) by Isothermal Titration Calorimetry. We discussed how amino acid substitutions and peptide helicity enhanced/weakened their affinities for *c-MYC* quadruplexes and characterized specific non-covalent inter-residual interactions determining their selectivity. Solution NMR structure indicated that KR12C, the best peptide candidate, selectively stabilized the 5′-propeller loop of *c-MYC* quadruplex by arginine-driven electrostatic-interactions at the sugar-phosphate backbone while KR12A peptide destabilized the quadruplex inducing a single-stranded hairpin-like conformation. Chromatin immunoprecipitations envisaged that KR12C and KR12A depleted and enriched Sp1 and NM23-H2 (Nucleoside diphosphate kinase) occupancy at Pu27 respectively supporting their regulation in stabilizing and unfolding *c-MYC* quadruplex in MCF-7 cells. We deciphered that selective arresting of *c-MYC* transcription by KR12C triggered apoptotic-signalling pathway via VEGF-A-BCL-2 axis.

## INTRODUCTION

G-quadruplex nucleic acids have recently emerged as a new class of anti-cancer targets. These non-classical DNA structures are evolved over tandem guanine repeats with a stable core of π–π stacked G-quartets in a cyclic Hoogsteen hydrogen-bonding arrangement (1,2). Bioinformatic studies revealed around 3,70,000 putative G-quadruplex-forming sequences in the telomeres, promoters, and untranslated regions of the oncogenes (3–6). *In vivo* mapping of these consensus motifs by quadruplex-specific antibodies (e.g. BG4 antibody) reinforced their prevalent distribution into translocation hot-spots and primary tumors than normal tissues (7,8). These tetra-stranded structures play key functions in diverse cellular processes (e.g. transcription, translation, mRNA stability, etc.) and neoplastic transformation (e.g. genomic instability and genetic dysfunction) (9). Therapeutic implications of G-quadruplex structures have expanded during the past decade and gained a momentum with the finding that *c-MYC* transcription, which is upregulated in 80% of solid tumors, could be inversely regulated by stabilizing a G-quadruplex-motif (Pu27) in the Nuclease Hypersensitive Element (NHE) III_1_, located –142 to –115 base pairs upstream of *c-MYC* promoter (P_1_) (10).

In cancer biology, *c-MYC* earned a formidable oncogenic reputation due to its aberrant expression through constitutive transcription (11,12), which attributes to autonomous proliferation, chromosomal translocation, relentless replication, and impaired apoptosis in a myriad of cancers (13–18). Earlier reports provided an estimate of ∼70,000 annual deaths in the United States to be associated with high level of *c-MYC* transcripts (17,19). MYC knockout experiments in the animal models demonstrated that partial inactivation of the oncogene offers acute or sustained tumor regression promoting proliferation arrest, apoptosis, differentiation, and senescence in cancer cells (14,20,21).Therefore, the design of *c-MYC-*targeted therapeutics to restore its basal level of cellular expression holds a promising avenue in anti-cancer treatment (22). In normal proliferating cells, the 27-mer quadruplex structure (Pu27) in NHE III_1_ negatively regulates *c-MYC* transcription and ensures a threshold level of *c-MYC* transcripts. In Burkitt's lymphoma, reciprocal translocation shifts *c-MYC-*NHE III_1_ under the control of an ectopic promoter (in Ramos cells) or leads to a Pu27-deleted (8,14) *c-MYC* allele (in CA46 cells) resulting constitutive *c-MYC* transcription (23–26). Simonsson and colleagues showed that the treatment of CA46 cells by synthetic 22-mer quadruplex substantially inhibits *c-MYC* transcription (10,26), which underscores the biological and clinical significance of Pu27 motif in cancer therapeutics. However, the major caveat in this study is the conformational heterogeneity in Pu27 showing a high likelihood of dynamic shuffling between intermediate quadruplex isomers (27–31). Despite having the solution NMR (Nuclear Magnetic Resonance) structures of different loop isomers of Pu27 (28–33), *c-MYC* quadruplex stabilizing compounds suffer from conformational rigidity (e.g. Bisquinolinium derivatives (34–36)), lack of bioavailability (e.g. Quarfloxin) (37), and promiscuity to other oncogenic quadruplexes (e.g. thiazole peptides, Indolylmethyleneindanone derivatives, etc.) (38–40). Most of these ligands have aromatic/pseudoaromatic π-delocalized system and stabilize *c-MYC* quadruplex via external π–π stacking interaction rendering them inefficient to distinguish the intracellular quadruplexes of various topologies. This is because as many as ∼3,70,000 sequences in human genome have the propensity to evolve quadruplexes and a vast majority of known quadruplex-interacting molecules are designed to make high-affinity stacking interactions with G-quadruplexes. They also fail to conform to the polymorphic and flexible skeleton of native quadruplex and remain ineffective from target recognition due to evolution of interconvertible quadruplex intermediates within Pu27 under cellular microenvironment (41–44). Therefore, small compounds, designed to stabilize *c-MYC* quadruplex often encounter off-target effects and narrow therapeutic window limiting their anti-cancer activities (45).

In the light of these shortcomings, peptides offer an alternative and promising avenue for G-quadruplex-targeted anti-cancer therapeutics. Compared to other ‘biologics’ treatment (e.g. protein drugs and antibodies), peptides benefit from their smaller size, ease of synthesis and purification, cell permeability, tumor-penetrating ability, and improved biocompatibility (46,47). They exhibit minimal drug-drug interaction, less immunogenicity, faster blood clearance, and better intra-tumoral diffusion due to lower molecular weight and excellent tolerability in the patients. Therapeutic peptides are also amenable to rational design for high-affinity intracellular interaction with target quadruplexes (48). Opposed to the small molecules, peptide candidates provide exquisite specificity to their *in vivo* targets due to structural and functional diversity that enable them to fold into a complementary shape relative to the dynamic topology of the target quadruplex. Furthermore, they interact with the larger portion of the target quadruplex rendering higher action potencies and greater specificity with relatively few off-target side-effects (49). Unlike small molecules, peptides degrade into naturally occurring amino acids without accumulating toxic metabolites into specific organs. Indeed, peptide drugs are susceptible to cleavage by serum proteases causing shorter half-lives and lower stability accounting for their limited oral administration, difficult intracellular delivery, and the necessity of high doses. However, adding several steps to their manufacturing, *e.g*. blocking the C- and N- termini, use of unnatural or D-amino acids, and chemical modifications can circumvent these drawbacks improving their pharmacodynamic and pharmacokinetic properties (46,50). Neidle's group was the first to develop substituted acridines with amide bonds (3,6,9-trisubstituted acridines) as effective G-quadruplex stabilizers and telomerase inhibitors (51). Balasubramanian's group developed peptide-hemicyanine ligands (52), 3,6-bispeptideacridone- and 3,6-bispeptide–acridine conjugates showing greater binding affinity to telomeric G-quadruplexes (53,54). Linear and branched peptides with nonstandard amino acids and/or acridone, methylpyrrole, or methylimidazole derivatives are reported to significantly increase the quadruplex-to-duplex selectivity (55). Planar peptide macrocycles with an extended π-delocalized system (e.g. oxazole-based peptide macrocycles) offer preferential binding to various oncogenic G-quadruplexes (56). Zagotto *et al.* constructed a small library of 2,6- and 2,7-amino-acyl/peptidyl anthraquinones that inhibits telomerase activity by inducing G-quadruplex structure formation in human telomeres (57). Similarly, a number of Distamycin A analogues (e.g. amide-linked oligopyrroles, uncharged *N*-methyl amide analog, and carbamoyl analog) and peptide–nucleic acid oligomers (PNA) are designed to selectively stabilize telomeric and oncogenic quadruplexes (58–61). With regard to the derivation of protein sequences, an arginine–glycine rich (RGG) peptide from fragile X-mental retardation protein (FMRP) showed higher affinity to G-quadruplex structures (62). Insulin and Insulin-like growth factors (IGF-2) are also observed to selectively interact with the antiparallel G-quadruplex in the Insulin-linked polymorphic region (ILPR) (63).

In this study, we formulated six peptides derived from human Cathelicidin LL37 protein, secreted by lungs epithelial cells (64). Earlier studies evidenced that LL37 binds to telomeric G-Quadruplex structures but shows non-specific interactions with other oncogenic quadruplexes (65). This coupled with a major shortcoming that the 37-mer protein is cell membrane-impermeable; we truncated its quadruplex-binding domain (FK13) and further reengineered its mutants (KR12A, KR12B, KR12C, KR12D and KR12E) to offer enhanced G-Quadruplex affinity and therapeutic efficiency via selective interaction with *c-MYC* quadruplex under cellular microenvironment. We first evaluated their intracellular selectivity for native *c-MYC* quadruplex (Pu27) and its conformationally restricted isomers (Myc22 and Pu19) over different oncogenic and telomeric G-quadruplexes by dual-luciferase assays and isothermal titration calorimetry (ITC). Then, we investigated how amino acid substitution in different peptides modulates their binding affinities for *c-MYC* quadruplexes *in vitro* and under macromolecular crowding conditions. We analysed the thermodynamic partitioning of binding free energies between entropic and enthalpic components of peptide–quadruplex association and determined the role of peptides to affect the thermal stability of *c-MYC* quadruplexes by circular dichroism (CD). We correlated differential binding profiles of the peptides and *c-MYC* quadruplex with detailed structural investigations at the atomic level by solution NMR (nuclear magnetic resonance) structure calculations and Molecular Dynamics (MD) simulations. In addition, we monitored the role of peptides to regulate the occupancy of numerous transcription factors (Sp1, RNA Polymerase II, NM23-H2 (Nucleoside diphosphate kinase), and Nucleolin) at NHE III_1_ by siRNA knockdown and chromatin immunoprecipitation (ChIP) studies, which appended a deeper insight into peptide-driven transcription regulation in cancer cells. Based on these observations, we screened the best peptide candidate having higher intracellular selectivity to *c-MYC* quadruplex and determined its mode of action to drive apoptotic signalling in cancer cells by monitoring differential expression of the apoptotic markers. This study gives fundamental insights at the atomic level how site-specific substitution of the amino acids in peptides determines the folding and unfolding of the quadruplex. These α-helical peptides offer overlapping target-specific pharmacophore model to target the dynamic structure of Pu27 and open a new paradigm for next generation quadruplex-targeting peptides with higher therapeutic indexes and minimal off-target effects.

## MATERIALS AND METHODS

### Materials

All Fmoc-protected amino acids, benzotriazol-1-yl-oxytripyrrolidinophosphonium hexafluorophosphate (PyBOP), and Rink amide MBHA resin are purchased from Novabiochem (Merck). *N*,*N*-Dimethylformamide (DMF), diethyl ether, trifluoroacetic acid (TFA), anisol, triisopropylsilane (TIS), phenol and *N*,*N*-diisopropylethylamine (DIPEA), piperidine and dimethyl sulfoxide (DMSO) are procured from Merck. Methanol, HPLC-grade water and acetonitrile are purchased from Rankem. For cell culture, Dulbecco's modified Eagle's medium (DMEM) and Gentamicin are obtained from Himedia laboratories. Trypsin–EDTA solution, Amphotericin B and DMSO for cell culture are bought from Sigma-Aldrich. Penicillin–streptomycin and fetal bovine serum (FBS) are purchased from Invitrogen. Single-stranded oligonucleotide sequences (Supplementary Table S1) for biophysical studies and the primers for real time polymerase chain reactions (qPCR) (Supplementary Table S2) are procured from Eurofins India Pvt Ltd. and Integrated DNA Technologies (IDT) respectively. For chromatin immunoprecipitations (ChIP), ChIP-grade anti-nucleolin primary antibody and NM23-H2 antibody (L15) are purchased from Abcam, and Santa Cruz laboratory respectively. Anti-Sp1 antibody and Rpb1 (RNAPII subunit b1) CTD (C-terminal domain) 4H8 antibody are obtained from Sigma and Cell signaling technologies respectively. For western blot experiments, anti-c-MYC (Rabbit polyclonal) is purchased from Cell Signalling Technology Inc. For the derivation of the signaling pathway, Mouse anti-BCL-2 (mouse monoclonal) and anti-β-actin (mouse monoclonal) are bought from Santacruz Biotechnology Inc. Other primary antibodies (anti-E2F1, anti-VEGF-A, anti-BAX, anti-P53, anti-APAF1, anti-Caspase 8, anti-PARP) are obtained from Abcam.

### Peptide synthesis

Peptides (Table 1) are synthesized in Solid phase Peptide synthesizer (Aapptec Endeavor 90) using the principles of Fmoc chemistry. Fmoc-protected amino acids are sequentially coupled followed by fmoc deprotection using 20% piperidine solution for 60 and 40 min, respectively. DIPEA and PyBOP are used as activator base and activator respectively. After washing in DMF, peptide-attached resin is cleaved by standard resin cleavage cocktail solution containing 92.5% TFA, 2.5% milliQ water, 2.5% TIS, and 2.5% phenol. The cleaved filtrates are precipitated using diethyl ether solvent and purified using reverse phase HPLC system (SHIMADZU, Japan) with Phenomenix C18 column. Peptide masses are determined by MALDI-TOF mass spectrometry (Bruker). (Supplementary information Section 5).

**Table 1.** Synthetic Peptides used in the study. Amino acid sequences are written in N (-NH2)- to C (-COOH)- direction

	Peptide sequences
FK13	N – FKRIVQRIKDFLR – C
KR12A	N – KRIVQRIKKWLR – C
KR12B	N – KRIVKRIKKWLR – C
KR12C	N – KRIVKLIKKWLR – C
KR12D	N – KRIVKVIKKWLR – C
KR12E	N – KRIVKRIKKWLL – C

### Cell culture

*c-MYC* overexpressing human breast adenocarcinoma cell line (MCF-7) (66) (ATCC) is cultured in complete DMEM medium, supplemented with 10% (v/v) FBS, 2 mM l-glutamine, 50 μg/ml Gentamicin, 1% Pen-Strep and 2.5 μg/ml Amphotericin B in a fully humidified CO_2_ incubator (ESCO cell culture CO_2_ Incubator, Model no. CCL-1708-8-UV) at 37°C and 5% CO_2_.

### Transfection of luciferase plasmids and dual-luciferase assays

MCF-7 cells are sub-cultured into 24-well microtiter plates at a cell density of 1 × 10^5^ cells/well. Reporter constructs (*c-MYC, BCL-2, KRAS* and *VEGF-A*) (500 ng) with and/or without the G-quadruplex scaffolds triggering the expression of *Renilla* luciferase are co-transfected with 50 ng of pGL3-control vectors (Promega) (used as internal control), encoding firefly luciferase into MCF-7 cells using Lipofectamine^®^ 3000 transfection reagent (Thermo-Fisher Scientific) following manufacturer's recommendations (Supplementary information Section 1). Twenty four hours post-transfection, cells are treated with peptides (FK13, KR12A, KR12B, KR12C, KR12D and KR12E) at an increasing concentration gradient.

Dual-luciferase assays are performed to examine the intracellular selectivity of peptides for *c-MYC* quadruplex isomers (Pu27, Myc22 and Pu19) over other oncogenic quadruplexes (*BCL-2, VEGF-A* and *KRAS*) of different topologies. The role of peptide-quadruplex complex in oncogene promoter activation is investigated in contrast to the GQ-null promoter constructs into MCF-7 cells (67). After 24 h of peptide treatment, cells are washed and lysed into 100 μl 1× Passive lysis buffer (PLB) for 15 min at room temperature. *Renilla* and firefly Luciferase activities in 25 μl lysate are quantified by Dual-Luciferase^®^ reporter assay system (Promega) as per manufacturer's recommendations. *Renilla* luciferase enzyme activity is normalized to that of the firefly luciferase activity and luminescence of the peptide-treated samples are normalized to the untreated ones, which yield the fold induction values for *Renilla* activities by different quadruplex structures in presence and absence of synthetic peptides. Luminescence of each sample is detected in GloMax^®^ 20/20 Single-Tube Luminometer (Promega) in triplicates and averaged from three independent experiments.

### Real time PCR

To inspect the transcription inhibitory roles of peptides in *c-MYC, BCL*-2, *VEGF*-A and *KRAS* oncogenes having putative quadruplexes in their promoters, we performed real time polymerase chain reactions. MCF-7 cells are sub-cultured into 6-well microtiter plates at a density of 1 × 10^6^ cells per well and treated with different concentrations of peptides (FK13, KR12A, KR12B and KR12C) for 24 hours. Total RNA is isolated from both untreated and treated cells using TRIzol method (Invitrogen) as per manufacturer's instructions. 2 μg of total RNA is processed for cDNA synthesis and reverse transcribed using a Super MuLV RT Kit (Biobharati Life Sciences Pvt. Ltd.). Real time PCR is performed using Maxima SYBR Green/ROX qPCR Master Mix (2×) (Thermo-Scientific) as per manufacturer's protocol. Housekeeping gene, *GAPDH* is used as an internal control to normalize the variability in *c-MYC* mRNA expression levels (Supplementary information Section 1). PCR primers are designed using Primer-BLAST, NCBI, and analyzed in OligoAnalyser 3.1-IDT (Supplementary Table S2).

### Oligonucleotide sequences

The putative wild-type and truncated G-quadruplex forming sequences (Table 2 and Supplementary Table S1), located at the upstream of *c-MYC* promoter are reconstituted in 10 mM Potassium Phosphate buffer (pH 7.0) having 100 mM potassium chloride (KCl). The sequences are allowed to evolve G-quadruplex structure in solution by annealing at 95°C followed by gradual cooling. (Supplementary information Section 1).

**Table 2.** The putative wild-type (Pu27) and truncated quadruplex–forming oligonucleotide sequences (Myc22 and Pu19) used in the biophysical studies

	Oligonucleonucleotide sequences
Pu27 (wild-type)	5′- T GGGG A GGG T GGGG A GGG T GGGG AA GG -3′
Pu19	5′ – T GGGG A GGG T GGGG A GGG T – 3′
Myc22	5′ – GGA GGG T GGGG A GGG T GGGG AA – 3′

### Circular dichroism

To understand the role of peptides (FK13, KR12A, KR12B, KR12C, KR12D and KR12E) in stabilizing native and truncated isomers of *c-MYC* G-quadruplex, we have performed temperature-driven CD melting experiments for free and peptide-bound complexes in JASCO-J815 CD spectrometer. We have gradually titrated the peptides into 10 μM of putative quadruplexes and acquired the spectra after 5 min of peptide addition to allow complex formation and equilibration. Samples are heated from 20 to 95°C having the temperature gradient and the delay time of 2.5°C/min and 150 s respectively. Since the quadruplexes unfold at higher temperatures and revert back to their initial conformations upon renaturation, we have assumed a ‘two-state transition’ model (folded and unfolded) to analyze the melting curves and estimated the melting temperatures (*T*_m_) by fitting the data points into sigmoidal curves (Supplementary information Section 1).

### Isothermal titration calorimetry (ITC)

Thermodynamic attributes of the interaction profiles between peptides and putative quadruplex sequences are analysed by ITC using iTC200 Microcalorimeter at 25°C. Synthetic peptides (FK13, KR12A, KR12B, KR12C, KR12D and KR12E) are diluted to 20 μM in 10 mM Potassium phosphate buffer (10 mM K_2_HPO_4_ + 10 mM KH_2_PO_4_) containing 0.1 M KCl (pH 7.0). Syringe is filled with 500 μM quadruplex sequences present in the upstream promoter regions of *c-MYC* (Pu27, Pu19, and Myc22), *BCL-2* (Pu30), *VEGF-A* (Pu22), *KRAS* (Pu32) oncogenes, and telomere (Tel26) dissolved into the same buffer. We also carried out the experiment for KR12C and Pu27 using MCF-7 nuclear extract as the solvent to mimic the cellular microenvironment and impart macromolecular crowding during binding events. Same concentration of oligonucleotides is injected into the identical buffer or in nuclear extract without peptides to normalize the heat of dilution of peptide-quadruplex association to that of the control before curve-fitting. Oligonucleotides are injected 20 times at an interval of 150 s into the calorimeter cell having the peptides to achieve binding saturation. The heat of reaction per injection (μcal/s) is determined by integration of the peak areas using in-built Origin 7.0 software that provides the best-fit values of enthalpy (Δ*H*) and entropy (Δ*S*) of binding, the binding stoichiometry (*n*), and dissociation constant (*K*_d_) (Supplementary Tables S5(a), S5(b) and S5(c)). Data points are further simulated with ‘one-site’ binding modes (Supplementary information Section 1).

### Molecular docking and simulations

To analyze the stable non-covalent interactions between native *c-MYC* quadruplex and synthetic peptides (FK13, KR12A, KR12B, KR12C, KR12D, and KR12E), we performed blind docking and constraint-driven (5′- and 3′-side of Pu27) docking employing Patchdock and Firedock webservers. Docking benchmarks are established by superimposing the docked poses followed by constructing docking clusters reproduced independently by the docking protocol. To inspect further, we minimized the docking complexes in AMBER14 and calculated all atom root mean square deviation (RMSD) values for each pose (Supplementary Table S3). The docking cluster(s) having the RMSD of 1.0–2.0 Å are considered as the near-native conformations and are taken up for the Molecular Dynamics (MD) simulation for the validation and evaluation of the binding pose(s) and molecular interactions (Supplementary information Section 1).

### 1D and 2D ^1^H–^1^H NMR spectroscopy

NMR experiments are performed in Bruker AVANCE III 700 MHz NMR spectrometer, equipped with a 5 mm SMART probe. 100 μM of KR12C is titrated into 500 μM Myc22 in the identical annealing buffer containing 90% water and 10% D_2_O. Both one- and two-dimentional the experiments are carried out in 5 mm NMR tubes having an active sample volume of 600 μl. The spectra are referenced to an internal standard, TSP (3-(trimethylsilyl)-2,2′,3,3′-tetradeuteropropionic acid) at 0.0 ppm. The exchangeable and non-exchangeable protons of Myc22 are observed in the one-dimensional proton spectra under free and KR12C-bound conditions using Bruker Pulprog ‘zgesgp’ with a spectral width (sw) of 20 ppm, number of scans (ns) of 512, and calibrated pulse length (p1) of 12.48 μs. 2D ^1^H-^1^H NOESY spectra of free and peptide–DNA complex are collected using Bruker's ‘noesyesgpph’ pulse program at 15°C with a mixing time of 300 ms. Data is processed using Lorentzian-to-Gaussian filtering functions applied in both dimensions, and zero filling to 2048 (*t*_1_) and 1024 (*t*_2_) data points. Time domain data sets consist of 2048 × 256 complex data points in *t*_2_ and *t*_1_ dimensions respectively. Structure calculations of free Myc22 are performed as recommended in the previously published paper (68). 2D spectra are further processed and analysed in Topspin v3.1 and SPARKY softwares for structure calculations. Exchangeable and non-exchangeable protons of Myc22 are observed in the one-dimensional proton spectra under free and KR12C-bound conditions using Bruker Pulprog ‘zgesgp’ with a spectral width (sw) of 20 ppm, number of scans (ns) of 512, and calibrated pulse length (p1) of 12.48 μs. 2D ^1^H–^1^H NOESY spectra of free quadruplex and peptide–quadruplex complex are collected in 90% H_2_O and 10% D_2_O using Bruker's ‘noesyesgpph’ pulse program with a mixing time of 300 ms (Supplementary material section 1).

### Structure calculation and refinement

Three-dimensional structure of Myc22-KR12C complex is calculated following NMR restrained molecular dynamics (MD) simulation. The docked structure of Myc22–KR12C complex (Cluster 4) showing high agreement with the intermolecular NOE cross-peaks is selected to develop the parameter and coordinate files using ff14SB and gaff force-fields. Tleap program of AMBER14 is employed to read the force-fields, coordinates, and topology information. Then, distance restraints are simulated from NOE cross-peak volumes and intensities into NOESY spectra. NOE intensities are qualitatively classified as strong, medium, and, weak having the upper distance limit of 3.0, 4.0 and 6.0 Å and the lower limits of 1.8–3.0 Å respectively. Then, NMR distances, dihedral restraints, and structure statistics are given for structure refinement (Supplementary Table S6). Restraint files are processed in AMBER14 using nmropt = 1 and then the model is minimized along with the lower and upper bound force constants of 2 kcal.mol^−1^. A simulated annealing of 20 ps is further carried out in vacuum with Generalized Born Model. The model is first heated upto 400 K for 10 ps, and then gradually cooled to 300 K in next 10 ps, and finally cooled to 0 K for next 5 ps. The cycle is repeated thrice. PyMOL and Maestro softwares are used to visualize and validate the solution NMR structure (Supplementary information Section 1).

### Chromatin immunoprecipitation (ChIP) assays

Chromatin immunoprecipitation studies are conducted to monitor the promoter occupancies of different transcription factors (Sp1, NM23-H2 and Nucleolin) and RNA polymerase II (RNAPII) on the region encompassing *c-MYC-*NHE III_1_ (69). We have grown MCF-7 cells in 10-cm^2^ culture flask at a density of 1 × 10^6^ cells per well and treated with the synthetic peptides (FK13, KR12A, KR12B, and KR12C) for 24 hours. Cells are cross-linked with 1% formaldehyde at room temperature for 10 min and the reaction is stopped by 0.125 M glycine at room temperature for 10 min. Fixed cells are lysed and sonicated to yield DNA fragments of 200–500 bp. ChIP-grade antibodies (NM23-H2 (L-15) Antibody (sc-14790, Santa Cruz), and Anti-Nucleolin antibody [4E2]-ChIP Grade (Abcam)), Anti Sp1 antibody (Sigma), and Rpb1 (RNAPII subunit b1) CTD (C-terminal domain) 4H8 antibody are added to the sonicated chromatin and incubated overnight at 4°C. Then, 10 μl protein A magnetic beads (Dynal, Invitrogen), previously washed in RIPA buffer are added to the samples and bead–protein complexes are washed thrice with RIPA buffer and twice with TE buffer. The genomic DNA is eluted for 2 h at 68°C in complete elution Buffer (20 mM Tris, pH 7.5, 5 mM EDTA, 50 mM NaCl, 1% SDS and 50 μg/ml proteinase K) and further combined with the DNA eluted from second elution for 10 min at 68°C in 100 μl of elution buffer (20 mM Tris, pH 7.5, 5 mM EDTA and 50 mM NaCl). ChIP-isolated DNA is purified using MinElute Purification kit (Qiagen) and is amplified by PCR (Polymerase Chain Reaction) reactions using forward and reverse primers (70) (Supplementary Table S4) specific to the quadruplex-enriched regions at *c-MYC* promoter and Phusion® High-Fidelity PCR Kit (NEB). Anti-rabbit IgG is employed for mock immunoprecipitation (Negative control).

### Western blot

To observe the effect of KR12C in c-MYC protein expression, and to decipher the peptide-driven signaling pathway to trigger apoptosis within cancer cells, we have performed an array of Western Blot experiments. MCF-7 cells are seeded at a density of 7 × 10^5^ cells per well into 60 mm dishes and allowed to attach overnight. Next day cells are serum starved for 18 h. At the end of 18 h fresh medium supplemented with 2% FBS +1% antibiotic and anti-mycotic is added. Then cells are treated with 5 μM and 10 μM KR12C. Recombinant VEGF is added at a concentration of 200 ng/ml of medium either alone or in combination with 10 μM KR12C. Cells are harvested after 24 hrs of treatment.

Whole cell lysate is prepared using the Cell Lysis Buffer (Cell Signalling Technologies) according to the manufacturer's protocol supplemented with Phenylmethylsulfonyl fluoride (PMSF) (Merck) and Protease Inhibitory Cocktail (PIC) (Merck). 40 μg of protein is separated using sodium dodecyl sulfate polyacrylamide gel electrophoresis (SDS-PAGE). (4% stacking gel and 12–15% resolving gel). Samples are transferred to polyvinylidene fluoride (PVDF) membranes (Immobilon-P, EMD Millipore). Membranes were blocked using 5% milk in Tris-buffered saline (TBS) with Tween 20 (Amresco) for 45 min at room temperature. Membranes are then incubated overnight at 4°C with the following antibodies: anti-c-Myc (Rabbit polyclonal, 1:500 dil. Cell Signalling Technology Inc.), Mouse anti-Bcl2 (Mouse monoclonal, 1:250 dil. Santacruz Biotechnology Inc.), anti E2F1, anti VEGF-A, anti-P53, anti-APAF1, anti-Caspase 8, anti-PARP (Abcam), anti-β-actin (Mouse monoclonal, 1:10 000 dil. Santacruz Biotechnology Inc.). Next membranes are incubated with peroxidase conjugated anti-rabbit IgG raised in Goat (Sigma, 1:8000 dil.) and peroxidase conjugated anti-mouse IgG raised in Rabbit (Sigma, 1:10 000 dil.) for 2 h at room temperature. Target proteins are visualised on membranes using Clarity TM Western ECL substrate (Bio-rad) and images are captured using ChemiDoc MP system with Image Lab™ software for PC version 6.0 (Bio-rad).

## RESULTS

### Design and synthesis of the peptides from naturally occurring human Cathelicidin protein, LL37

We constructed a small library of six peptides by pruning the quadruplex interacting domain of LL37 and further by substituting and mutating the position of the amino acids in the peptide sequence (Table 1 and Supplementary Figure S1). We considered few criteria to enable the peptides with anti-cancer properties and selective affinity for *c-MYC* quadruplex. (i) The amphipathic and cationic nature of the peptides are retained such that the net positive charge ranges between +2 to +9 at pH 7. Due to higher expression of phosphorylserines and *O*-glycosylated mucins, cancer cell membrane carries a net negative charge. Therefore, we put Arginine and lysine residues at termini to attract more phosphates and water to make stable amino acid-phosphate clusters across plasma membranes, which plays a central role in lipid membrane disruption and permeabilization (71–73). (ii) We incorporated lysines and leucines at different positions of the peptide to increase the α-helical stability as they are more prone to pass through the plasma membrane. (iii) Due to unbranched side chains and accessible rotamers, lysines enrichment is hypothesized to make more electrostatic interactions with quadruplex sugar-phosphate backbone in the loop regions of *c-MYC* quadruplex having distinct topologies. (iv) Phenylalanine and tryptophan are rationalized to make stacking interaction to improve selectivity for the quadruplex structure.

### Intracellular selectivity of synthetic peptides for G-quadruplex structure at *c-MYC* promoter

**Figure 1.**
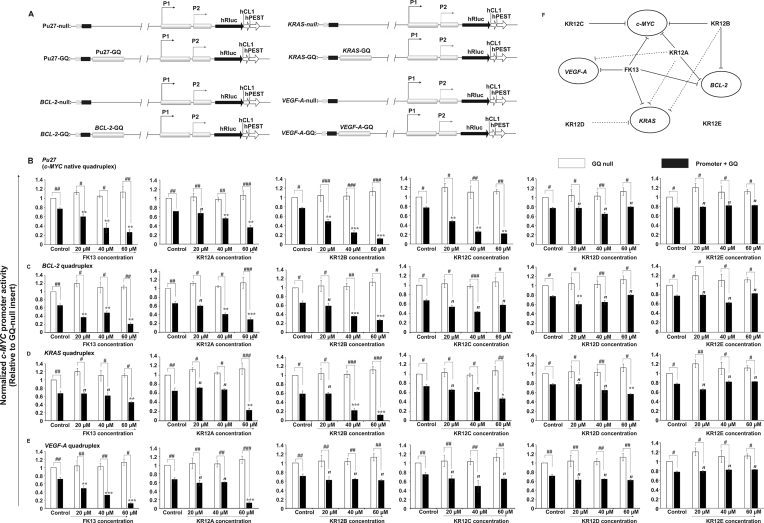
Conformer selectivity of the peptides for different oncogenic quadruplexes in MCF-7 cells. (**A**) pGL4.72[*hRlucCP*] vector having the inserts containing oncogenic promoter sequences (*c-MYC, BCL-2, KRAS* and *VEGF-A*) and upstream G-quadruplex forming elements ahead of the *hRluc* coding region. The promoter sequences are cloned into KpnI and HindIII restriction sites with or without the wild-type quadruplex scaffolds. *hRluc, Renilla* luciferase gene; *hCL1* and *hPEST*, protein destabilizing sequences; oriC, origin of replication; AmpR, ampicillin resistance gene; SV40 (Simian virus 40 polyadenylation signal cassette), P1 and P2, promoter sequences; null, constructs having no quadruplex motif upstream the oncogene promoters; GQ, G-quadruplex forming motif. (**B**) Dual-luciferase assays. Evaluation of the promoter activity of different oncogenes ((**B**) *c-MYC*, (**C**) *BCL-2*, (**D**) *KRAS* and (**E**) *VEGF-A*) of using the reporter plasmids with or without the wild-type quadruplex-forming sequences (Pu27-GQ, BCL-2-GQ, KRAS-GQ and VEGF-A-GQ) in MCF-7 cells. Relative promoter activity is determined by normalizing the Rluc/Fluc values to that of the cells transfected with P1-P2 promoter construct (GQ-null), having no quadruplex-forming motif. Error bars represent mean ± SE (*N* = 5). Statistical differences in the luciferase activities compared to the respective GQ-null constructs of the target oncogenes used one-way ANOVA followed by Tukey–Kramer Test (^#^*P* < 0.05, ^##^*P* < 0.01, ^###^*P* < 0.001). Statistical differences in the luciferase activities compared to that of the peptide-untreated cells used two-tailed Student's *t* test (*P < 0.05, **P < 0.01, ***P < 0.001). (**F**) Schematic diagram of the peptides having differential selectivity to oncogenic quadruplexes with various folding topologies and differential regulation of the promoter activies of target oncogenes by the peptides.

Peptide candidates suitable for the therapeutic interventions are expected to selectively interact with G-quadruplex scaffolds at the target oncogenes over duplex and other oncogenic quadruplexes under cellular environment. To evaluate the intracellular conformer selectivity of the peptides for *c-MYC* quadruplex isomers (Pu27, Myc22 and Pu19) over other oncogenic quadruplexes (*BCL-2, VEGF-A* and *KRAS*) of different folding topologies, we performed dual-luciferase assays in MCF-7 cells transfected with the luciferase constructs containing *Renilla* luciferase coding sequences downstream of the G-quadruplex scaffolds of the oncogenes of interest (Figure 1A and Supplementary Figure S2A). Earlier studies suggested that the peptides alter their secondary structures depending upon cellular microenvironment. In addition, conformational flexibility of the peptides is significantly implicated in their effective functional expression, diversification, and ability to penetrate the cell membrane (74,75). We determined the magnitude of promoter inhibition of the target oncogenes with an increasing gradient of peptides’ treatment (0–60 μM) considering that the stable association of peptides and quadruplexes in MCF-7 cells at the oncogene promoters would drive lower expression of downstream *Renilla* luciferase in contrast to the GQ-null plasmids. We observed that the constructs lacking G-quadruplex motifs (GQ null) in the oncogene promoters were constitutively activated irrespective of the peptide concentrations, while the promoters having G-quadruplex structures of various folding topologies manifested differential luciferase activities reflecting differential regulation of oncogene promoters by the peptides (Figure 1B–E). KR12D and KR12E had no significant effects upon oncogene promoters regardless of the upstream quadruplex motif(s), which envisaged their incompetence to recognize oncogenic quadruplexes *in cellulo*. However, at higher concentration, KR12D showed meager inhibitory effect (∼1.6-fold) upon *KRAS* promoter activity (Figure 1D and F). FK13 and KR12A abrogated *c-MYC* promoter activity (∼2-fold repression at 60 μM) dose-dependently. Coupled with that, they were observed to significantly downregulate the promoter activation of *VEGF-A* and *BCL-2*, which indicated their failure to discriminate different oncogenic quadruplex conformers and non-specific binding with different quadruplexes in the cells (Figure 1B, C, E and F). KR12A further repressed *KRAS* promoter activity (∼2.5-fold) at higher concentrations suggesting its weak interaction with *KRAS* quadruplexes *in cellulo* (Figure 1D and F). KR12B and KR12C selectively attenuated *c-MYC* promoter activation in a dose-dependent manner. While KR12B was found to show inverse regulation of *BCL-2* and *KRAS* promoters at higher doses, KR12C had no significant impact upon other oncogenic promoters, even at higher concentrations, conferring its conformer selectivity for *c-MYC* quadruplex *in cellulo* (Figure 1B, C, D and F). These results prompted us to investigate the intracellular selectivity of peptides for different loop isomers (Myc22 and Pu19) of native *c-MYC* quadruplex (Pu27). As per earlier investigations, Pu27 enables high likelihood of conformational dynamics in the cellular microenvironment involving dynamic shuffling of multiple overlapping quadruplex conformers (10,27). We monitored if these peptides could negatively regulate *c-MYC* promoter in presence of the biologically relevant and stable loop isomers (Myc22 and Pu19) of Pu27 (Supplementary Figure S2B). Interestingly, we observed that KR12C led to promoter attrition (∼5-fold) in presence of Myc22 and Pu19 isomers while the inhibitory role of KR12B remained weaker in Pu19 (Supplementary Figures S2C and S2D). FK13 and KR12A, at higher concentrations, attenuated *c-MYC* promoter activation under the control of Myc22 and Pu19 (Supplementary Figures S2C and S2D). Consistent with the earlier data, KR12D and KR12E exerted no regulatory effect in *c-MYC* promoter activation in presence of Myc22 and Pu19 conformers. This experiment allowed a rapid screening of synthetic peptides for their selective targeting at *c-MYC* quadruplex in the cells driving *c-MYC* promoter repression.

### Comparative analyses of the interaction profiles between peptides and *c-MYC* quadruplex isomers

**Figure 2.**
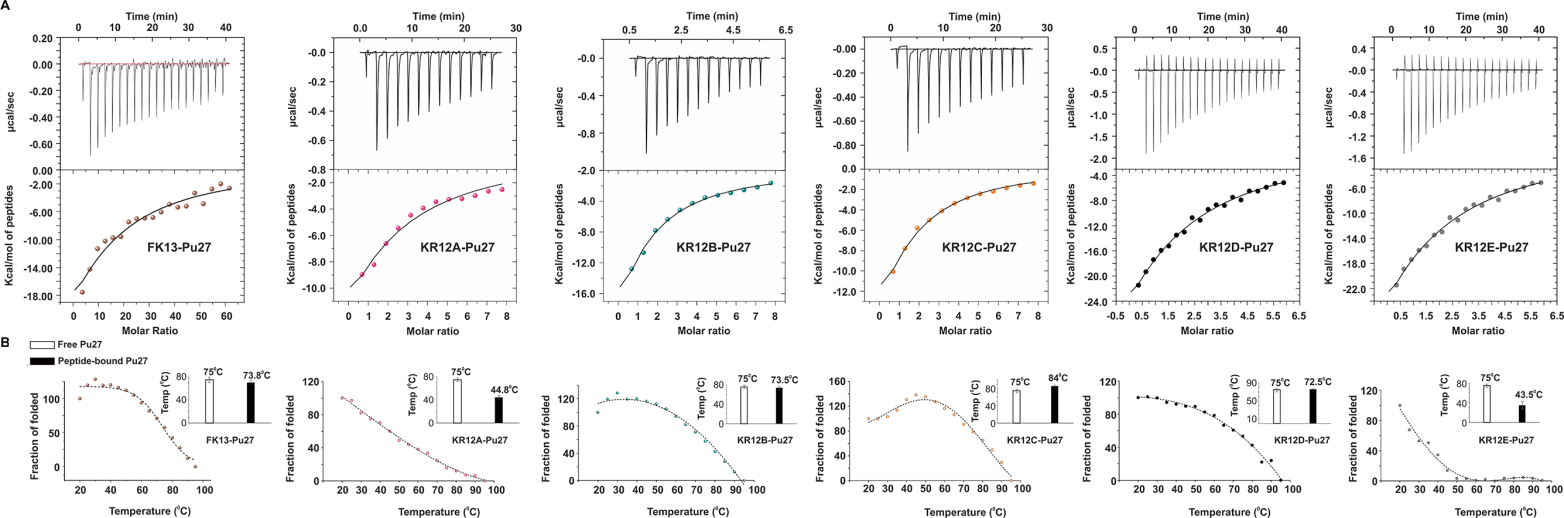
Comparative analyses of the thermodynamic profiling of peptide-Pu27 complexes. (**A**) Isothermal titration calorimetry showing intermolecular interactions in Potassium Phosphate buffer (pH 7.0) and 100 mM KCl. Top panels: enthalpic heat released versus time at 25°C during titrations. Bottom panels: thermogram of the integrated peak intensities plotted against the molar ratio of the complex. Best-fit curves using single-site binding model. (**B**) Melting curves of free and peptide-bound Pu27 complex by temperature driven CD (circular dichroism) spectroscopy. Data points denote fraction of folded quadruplex at each temperature. Sigmoidal fitting of the melting curves using two-state transition model (folding and unfolding) yields the melting temperature (*T*_m_). Bar plots denote the *T*_m_ of free and bound Pu27.

**Figure 3.**
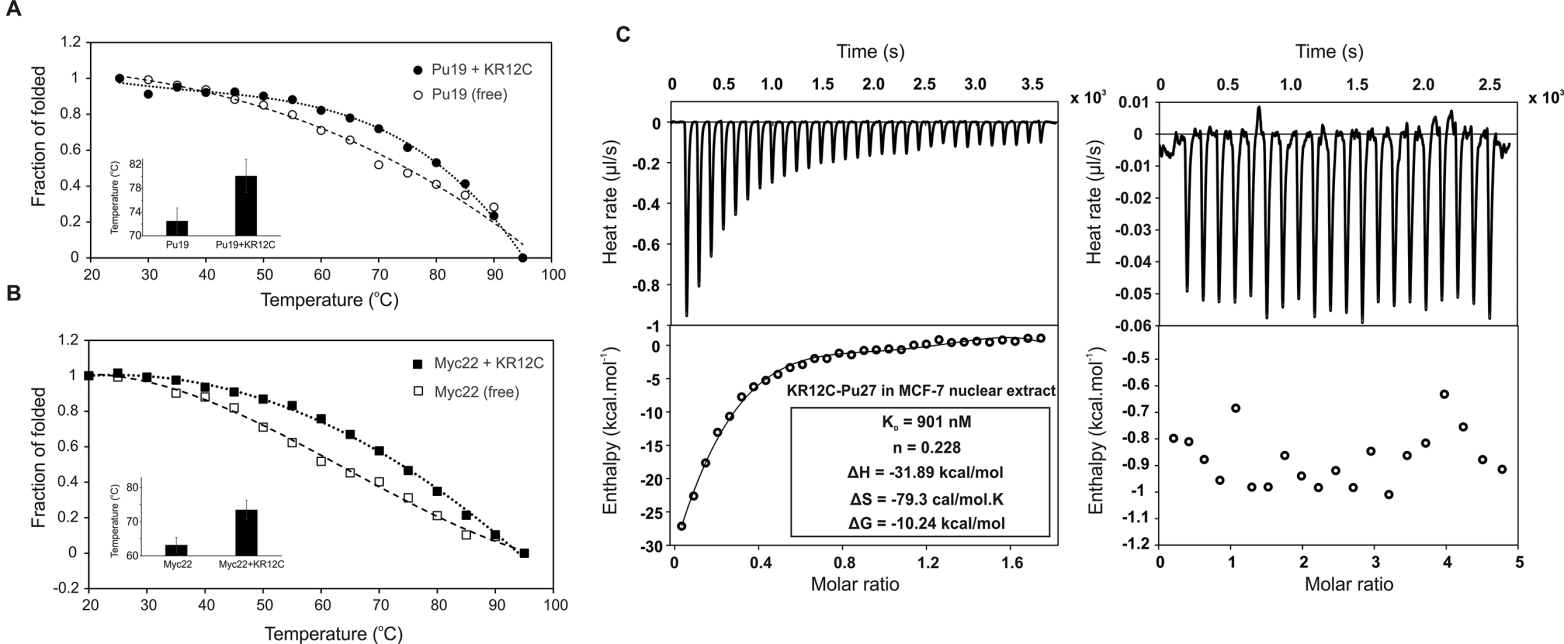
Conformer selectivity and stabilizing effect of KR12C for wild-type Pu27 and its different loop isomers. (**A, B**) CD melting curves of Pu19 and Myc22 with and without KR12C peptide. Bar plots indicate escalation of the melting temperature (*T*_m_) of Pu19 and Myc22 upon KR12C interaction. (**C**) Isothermal titration calorimetry showing intermolecular interactions between KR12C and Pu27 under macromolecular crowding conditions provided by MCF-7 nuclear extract (left). Top panels: enthalpic heat released versus time at 25°C during titrations. Bottom panels: thermogram of the integrated peak intensities plotted against the molar ratio of the complex. Best-fit curves using single-site binding model. Thermodynamic parameters of binding reactions and binding affinity of KR12C for Pu27 under macromolecular crowding conditions are provided. Binding isotherm is subtracted from the background titrations; i.e., oligonucleotides into MCF-7 nuclear extract (Right).

Luciferase assays provided a real time read out of *c-MYC* promoter regulation and quadruplex selectivity by synthetic peptides underpinning their different behaviour in *c-MYC* quadruplex interaction under cellular environment. This observation led us investigate the thermodynamic parameters of *in vitro* binding reactions between the peptides and different oncogenic quadruplexes (*c-MYC* (Pu27, Myc22, and Pu19), *BCL-2, VEGF-A, KRAS*) and telomeric quadruplex by Isothermal titration calorimetry (ITC). We slowly titrated different quadruplex sequences into synthetic peptides dissolved into Potassium phosphate buffer (pH 7.0) and analysed the integrated heat release of the titrations to explain the thermodynamic partitioning of binding free energy (Δ*G*) between enthalpic and entropic components. Indeed, we witnessed a series of enthalpy-dominated exothermic binding reactions between peptides and the quadruplexes since the binding enthalpy (ΔH) overcompensated for the unfavorable loss in conformational entropy (ΔS) (Figure 2A and Supplementary Figures S3–S5). The net entropy changes (ΔS) were negative values, which might attribute to the restriction of the conformational freedom at Pu27 interacting sites and release of the constrained water molecules upon complex formation (Table 3 and Supplementary Tables S5(a)–(c)). The magnitude of ΔH were relatively more negative than Δ*S*, hence contributed substantially to the binding free energies during the interactions (Table 3 and Supplementary Tables S5(a)–(c)). Consistent with the previous results of dual-luciferase assays, site-specific substitution of the amino acids in peptides offered differential magnitude of enthalpy–entropy compensation suggesting varying mode of interactions during the binding reactions between peptides and different quadruplexes. Replacing Q5 (in KR12A) with K5 in KR12B, KR12C, and KR12D significantly lowered their binding dissociation constants (*k*_d_) for Pu27 and its isomers as well as telomeric quadruplex (Table 3 and Supplementary Tables S5(a) and S5(b)). The amide side chain of Glutamine does not ionize at physiological pH while ϵ-amino group of lysine (p*K*_a_ > 10) remains positively charged at pH 7.0. This enabled the hydrogen donor ammonium (NH_3_^+^) in Lysine to favour the electrostatic interactions with the negatively charged sugar-phosphate backbone of quadruplex DNA resulting the binding ΔH relatively more negative. In contrast, FK13, which also has a glutamine at fifth position, showed enhanced affinity for Pu27 compared to KR12A, KR12B, KR12D and KR12E despite having lower binding affinity with the isomers of Pu27 and the telomeric quadruplex. Earlier studies showed that phenylalanines possess additional stability due to π-electron cloud across their aromatic rings, which enable them to make aromatic-aromatic interactions (76) and stacking interactions with nucleic acids. Phenylalanines also play important role in stabilizing α-helix and phe-phe pair in peptides are enthalpically favoured to impart additional stability in peptide–DNA complexes (77,78). FK13 has two phenylalanines at 1st and 11th positions, which might impart additional stability to the helicity of the peptide upon binding with Pu27 rendering improved affinity towards Pu27. On the other hand, single-residue replacement (R12 > L12) in KR12E at C-terminus severely compromised the binding affinity of KR12E for Pu27, Myc22, Pu19 and telomeric quadruplexes (Table 3 and Supplementary Tables S5(a) and S5(b)). Since the guanidium moiety of R12 has a greater intrinsic p*K*_a_ (<∼12) allowing its side chains to be invariably protonated at neutral pH (79), the peptides with C-terminal Arginine preferably made electrostatic interactions with quadruplex backbone, which concomitantly enhanced their binding affinity (except KR12E) for *c-MYC* quadruplexes. Furthermore, the conformational flexibility of Arginine-side chain at C-termini enabled the guanidinium group to search for polar atoms to satisfy its propensity to donate up to five hydrogen bonds to make more stabilized H-bonded network with the phosphate backbone of the quadruplex. This corroborated with the inefficiency of KR12E to recognize *c-MYC* quadruplex into NHE III_1_ in the cellular context. KR12B showed moderate binding affinity towards *c-MYC* quadruplexes and telomere. It was also found to inversely regulate the promoter activity of other oncogenes. Indeed, KR12B exhibited enhanced affinity for the quadruplexes compared to FK13 and KR12A due to having a greater net positive charge (+7). However, it failed to discriminate different quadruplexes like that of FK13 and KR12A under cellular milieu. This accounted for their non-specific interactions with different quadruplex topologies rendering FK13 and KR12B to be equally effective in reducing *c-MYC* promoter activity compared to KR12C. On the contrary, KR12C demonstrated ∼2-fold higher binding ΔH for Pu27 association and ∼5 times higher affinity for Pu27 (*K*_d_ = 8.01 μM) and its conformationally restricted biologically significant conformers (Myc22 and Pu19) compared to KR12B and KR12D (Table 3 and Supplementary Table S5(a)). This might account for the spatial position of K5. K5 in KR12C was buried in a non-polar environment in KR12C while was followed by a positive charge (R6) in KR12B. The p*K*_a_ of lysine side chain (ϵ-amino group) is subject to fluctuate depending upon the neighbouring residues (80,81), which might have altered its localized charge distribution and accessibility to make salt bridge interactions. In addition, KR12C showed very poor or no binding affinity with other oncogenic quadruplexes (*BCL-2, KRAS* and *VEGF-A*). It binds to *BCL-2* and telomeric quadruplex having ∼3- and ∼4-fold decreased affinity compared to *c-MYC* quadruplexes (Supplementary Figure S4 and S5 and Supplementary Table S5(b-c)). This was also in agreement with intracellular conformer selectivity of KR12C for *c-MYC* quadruplexes. However, peptide conformation is subject to change under cellular microenvironment. Therefore, peptide affinities for *c-MYC* quadruplex would be radically different in the cells compared to the potassium phosphate buffer. To address this concern, we reiterated the ITC experiment between KR12C and *c-MYC* quadruplex (Pu27) using MCF-7 nuclear extract as the solvent to mimic the cellular microenvironment and impart macromolecular crowding (82). We observed as significant as ∼8-fold increase in the binding affinity (*K*_d_ = 901 nM), which envisages a sharp enhancement in *c-MYC* quadruplex selectivity due to the crowding effect of macromolecules in MCF-7 nuclear extract (Figure 3C).

**Table 3.** Thermodynamic attributes of interaction between peptides (FK13, KR12A, KR12B, KR12C, KR12D and KR12E) and native *c-MYC* quadruplex (Pu27) calculated from Isothermal titration calorimetry. Binding enthalpy (Δ*H*), entropy (Δ*S*), binding energy (Δ*G*)

Complex	Δ*G* (kcal.mol^−1^)	Δ*H* (kcal.mol^−1^)	Δ*S* (cal/mol/deg)	*K*_A_ (M^−1^)	*K*_d_ (μM)
FK13-Pu27	−5.982	−77.8	−241	2.89 × 10^4^	34.6
KR12A-Pu27	−6.44	−72	−220	1.62 × 10^4^	61.73
KR12B-Pu27	−6.18	−137.3	−440	2.68 × 10^4^	37.31
KR12C-Pu27	−6.974	−47.8	−137	1.24 × 10^5^	8.06
KR12D-Pu27	−5.774	−136	−437	2.04 × 10^4^	49.01
KR12E-Pu27	−5.89	−168	−544	1.88 × 10^4^	53.19

Since Pu27 exhibits conformational dynamics between different isomers, its conformational entropy is correlated to a broadened energy landscape which enables free Pu27 to sample more conformational states than its peptide-bound counterparts (83). To evaluate if these peptides could alter the secondary structure of the quadruplexes and increase its thermal stability by decreasing the entropy of quadruplex unfolding, we titrated the peptides into G-quadruplexes and quantitated the changes in the melting temperature (Δ*T*_m_) of Pu27 and its conformers (Myc22 and Pu19) upon peptide binding (Figure 2B and Supplementary Table S5(a), Supplmentary Figures S6(A) and S6(B)). These peptides did not significantly affect the secondary conformation of the quadruplexes. However, striking find of the study was that KR12A and KR12E, which exhibited lower binding affinity as well as non-specific and negligible intracellular selectivity for *c-MYC* quadruplex, decreased the *T*_m_ of Pu27 by ∼30°C rendering conformational destabilization. In contrast, KR12C, which offered maximal affinity and intracellular specificity for Pu27 and its conformers (Myc22 and Pu19), promoted a sharp rise in the *T*_m_ by 9°C (Pu27), 6.91°C (Myc22), and 10.87°C (Pu19). This signified that KR12C could adopt a flexible α-helical conformation that allowed the peptide to conform to the flexible skeleton of Pu27 and stabilize the biologically significant quadruplex isomers of native *c-MYC* quadruplex (Figure 3A and B). This might attribute to the lysine in KR12C, which is known to produce a much greater population of accessible rotamers compared to arginine and glutamine causing enhanced stability of Pu27 by KR12C and a sharp decrease in the conformational entropy of Pu27 (Δ*S*) during unfolding (84). KR12B and KR12D showing moderate affinities for Pu27 did not significantly induce thermal stability. These observations gave a fair explanation to the varying intracellular specificity of peptides for *c-MYC* quadruplexes and their roles in differential promoter regulation.

### Differential affinity of peptides for Pu27 depends on their tethering point at the quadruplex and non-covalent interactions

The thermodynamic profiling of peptide–Pu27 association and their differential selectivity for Pu27 under cellular microenvironment impinge on the assessment of intermolecular interactions. Unfortunately, the structural ambiguity in Pu27 limits the mapping of the binding interface using solution NMR studies due to the broad envelope of overlapping 9-guaninyl-N1(H) resonances in the imino proton region (δ, 10–12 ppm) of Pu27. To resolve the problem, we performed constrained molecular docking between Pu27 and the peptides. We established docking benchmarks by clustering similar binding poses. The clusters with root mean square deviation (RMSD) of 1.0–2.0 Å were considered as the near-native conformations and taken up for the molecular dynamics (MD) simulation studies for the validation and evaluation of the intermolecular interactions (Supplementary Table S3). KR12C was involved in a multitude of electrostatic and weaker CH_3_–π interactions to impart selectivity and stability to Pu27 structure (Supplementary Figure S7A), confirmed by the average RMSD of the binding interface during simulation (Supplementary Figure S7B). The guanidino group of R12 attached to the sugar phosphate backbone of A6 by electrostatic interaction (Supplementary Figure S7C). Similarly, the penultimate R2 at N-terminus was clung to the sugar-phosphate backbone of G14 and G16 (Supplementary Figure S7D). These two interactions were highly stable and observed in more than 80% of the complexes in last 10 ns of the MD trajectories. They protected the G-quartet proximal at the 5′-propeller loop by gripping the backbone of the guanines delineating the quartet. Furthermore, L11 and I7 came into spatial proximity of the terminal quartet and stabilized the reverse Watson-Crick hydrogen bonding between A6 and A15 via CH_3_–π interactions (Supplementary Figure S7E). These were weaker interactions, wherein the delocalized π-electrons of Adenines served as the acceptors and the CH-vector directly pointed at the ring centre and was collinear with the ring normal, which maintained the integrity of the G-quartet (Supplementary Figures S7E and S7F)

FK13 involves R3 to make bisected electrostatic interactions with G3 and G4 bases and R7 to clip G14 backbone. In addition, the side chain of F11 is spatially inclined between 130° to 140° with the sugar backbone of Pu27 near 5′-terminus (Supplementary Figure S8A). Earlier studies showed that this type of interactions mediated by phenylalanines induce non-specific interactions at DNA grooves (85). Similar interactions were reflected in FK13-Pu27 complex, which agrees with the non-specific binding between FK13 and quadruplexes. In KR12A, K5 was replaced with Q5. This altered the helical property of the peptide during binding reaction such that L11 and I7 were far apart giving no protection to the A6-A15 pair. This destabilized the quartet, which corroborated with the decline of the thermal stability as observed in the CD melting experiment. However, KR12A was still able to tether to the quadruplex at C-termini wherein R12 and R6 played the key role to make H-bonds with sugar backbone of G6 and G20 respectively (Supplementary Figure S8B). In KR12B, R12 and K9 made an electrostatic interaction with G3 and G14 backbones respectively while R2 gripped G11 backbone of Pu27 via strong electrostatic interactions. L6>R6 replacement perturbed the helicity of KR12B that could no longer induce further stability to the quartet. KR12B formed a network of electrostatic interactions in quadruplex loop via terminal residues, which enhanced its affinity for quadruplex DNA at the cost of compromised selectivity for *c-MYC* quadruplex (Supplementary Figure S8C). In KR12D, inclusion of V6 incapacitated the helical stability, which hindered quadruplex stability and compromised its binding affinity for quadruplex. R2 and R12 developed transient hydrogen bond interactions with G11, G13, G4 and G5 backbones respectively, which accounted for its poor affinity to Pu27 (Supplementary Figure S8D). In KR12E, C-terminal R12 was replaced with L12 rendering it unable to grip the quadruplex backbone. Only the N-terminal K1 made transient H-bond with G3 backbone. R2 established electrostatic interactions with G4 backbone and K5 made transient polar contact with G11 (Supplementary Figure S8E). These interactions were less prevalent (observed in <60% in MD trajectory) due to solvent exposure that allowed water molecules to sequester the H-bond offering weaker interaction between KR12E and Pu27. This was also consistent with our previous results of KR12E-induced structural destability and poor binding affinity.

### Site-specific substitution of amino acids is the deterministic factor of quadruplex unfolding and stability

#### Stable association of KR12C at the 5′-quartet of Myc22 by solution NMR structure determination

**Figure 4.**
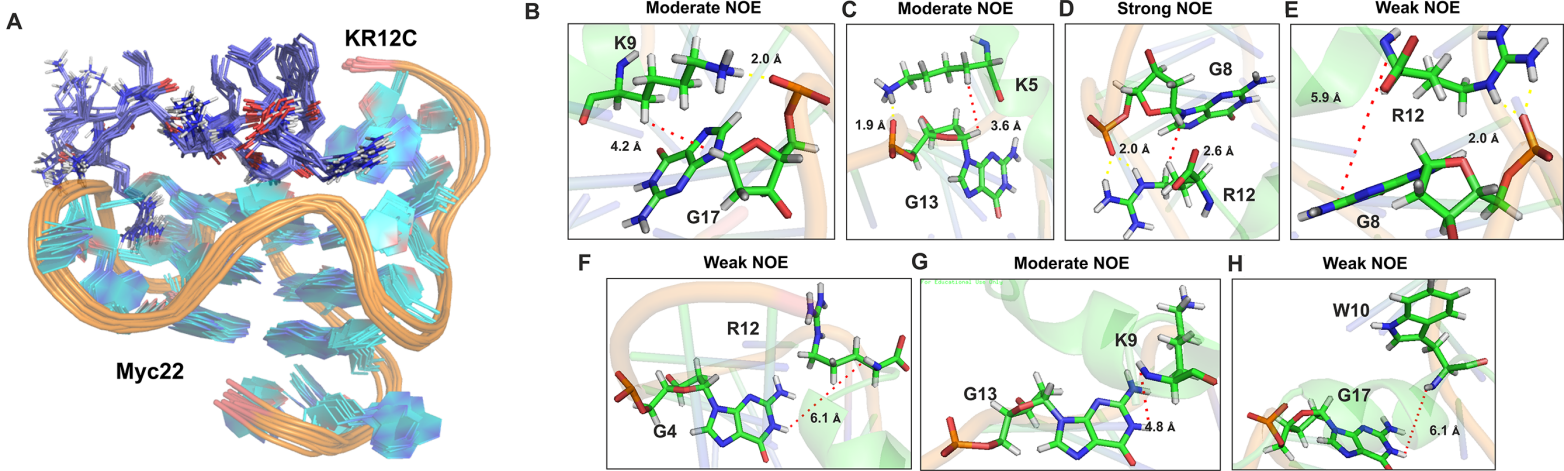
Solution NMR structure calculation of Myc22-KR12C complex. (**A**) Solution NMR structure of KR12C-Myc22 complex. An ensemble of 10 lowest energy structures of Myc22-KR12A complex. (**B**) Moderate NOE between K9(Hb)/G17(H1′) due to H-bond interaction at the sugar-phosphate backbone. (**C**) Moderate NOE between K5(Hb)/G13(H1′) favoured by electrostatic interaction between K5 and G13 at the backbone. (**D**) Strong NOE signal between R12(Hb)/G8(H1′) due to H-bond interaction at the backbone. (**E**) Weak NOE signal between between R12(NH)/G8(H1) due to H-bond interaction. (**F**) Weak NOE between R12(NH)/G4(H1). (**G**) Moderate NOE between K9(NH)/G13(H1). (**H**) Weak NOE between W10(NH)/G17(H1). Interatomic distances for NOE signals (in Red) and hydrogen-bond interactions (in yellow) are given in Å of the minimized structure.

So far, our investigations suggested opposing role of KR12A and KR12C in *c-MYC* quadruplex stability and binding affinities. MD simulations indicated that terminal positively charged residues of the peptides drove major intermolecular cationic contacts between KR12A-Pu27 and KR12C-Pu27. However, KR12A and KR12C differed by amino acid substitutions at fifth and sixth positions albeit having identical net charge (+6). To gain a detailed insight into the binding interface, intermolecular interactions, and the plasticity of the quadruplex structure upon peptide binding, we determined the solution NMR structure of Myc22-KR12C complex (Figure 4A). In this study, Myc22 was regarded as the biologically relevant quadruplex structure of Pu27 because it yields unambiguous assignment of the imino proton resonances in proton NMR spectra and retained the potential to downregulate *c-MYC* promoter activation. We performed ^1^H–^1^H NOESY (Nuclear Overhauser Effect Spectroscopy) experiment of unbound Myc22 and Myc22-complexed with KR12C at 6:1 molar ratio. NMR samples were prepared into 100 mM KCl and 10 mM potassium phosphate at pH 7.0_._ G-tetrad alignment was determined by the intra-residual connectivities of H1 and H8 protons in exchangeable proton NOESY spectra since base H8 of one guanine comes into closer vicinity to imino H1 of adjacent guanine upon Hoogsteen H-bond formation in the G-tetrad (Supplementary Figure S9). The H8/H1 intensities of G4/G8, G8/G13, G18/G13 and G10/G6 in the complex were significantly increased, which corroborated with KR12C driven enhanced stability of the quadruplex structure (Supplementary Figure S9). The glycosidic angles remained in anti-conformation, as confirmed by the H8–H1 NOE (nuclear overhauser effect) intensities. We observed the broadening of the imino signals between 10 to 12 ppm region upon KR12C titration, which indicated intermediate to slow exchange between the free and bound states or pre-equilibrium conformational change during binding reactions or the structural rearrangement and allosteric changes of the complex. The intraresidual H1′/H2′ and H4′/H1′ connectivites and interresidual NOE signals between H1′ of guanines and NH of amino acids supported that KR12C made electrostatic interactions with the sugar-phosphate backbone of quadruplex to alleviate the stability (Supplementary Figure S10). K9 and K5 strongly interacted with the sugar phosphate backbone of G17 and G13, which resulted moderate NOE signals between Hb of K9, K5 and H1′ of G17, G13 respectively (Figure 4B and C). Another electrostatic interaction between R12 and G8 backbone resulted strong Hb/H1′ crosspeak intensity, which in turn enabled the G8 base into spatial proximity of R12 reflecting weak NOE between N1(H1) of G8 and R12(NH) (Figure 4D and E). These compelling evidences confirmed that KR12C was associated at the 5′-quartet plane of Myc22 (Figure 4A) as to which weak to moderate intermolecular NOE signals were found between N1(H1) of G4, G17, G13 and NH of R12, W10 and K9 respectively (Figure 4F, G and H).

#### KR12A destabilizes c-MYC quadruplex by unfolding the quartets to single-stranded hairpin loop-like conformation

**Figure 5.**
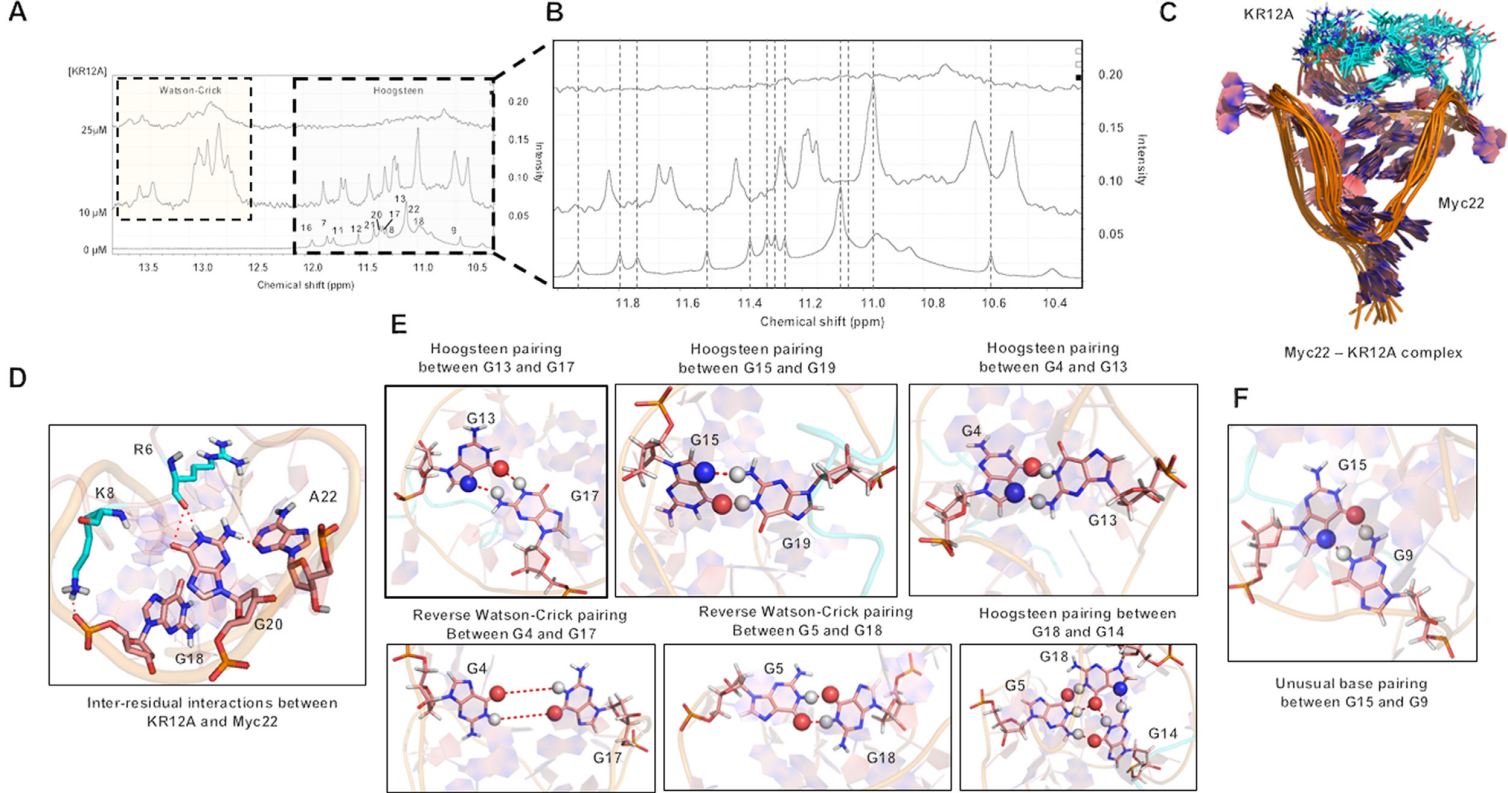
KR12A destabilizes Myc22 quadruplex by the formation of single-stranded hairpin like conformation. (**A**) Exchangeable proton resonances of Myc22 quadruplex (10–14 ppm) in 1D NMR spectra. KR12A is titrated into Myc22 quadruplex at increasing concentrations followed by incubation of 1 h at 25°C. (**B**) Enlarged region of 10–12 ppm of Myc22 and Myc22–KR12C complex. Chemical shift changes and broadening of the imino signals upon KR12A titrations. (**C**) *In silico* modelling of KR12A-Myc22 complex. An ensemble of 10 structures of Myc22–KR12A complex over last 10 ns of a 100 ns simulation in explicit solvent. (**D**) Major interresidual electrostatic interactions. R6 interacts with G20 base and K8 stabilizes the sugar-phosphate backbone of G18. (**E**) Induced Hoogsteen pairing (G13–G17, G15–G19, G14–G18 and G4–G13), Reverse Watson–Crick Pairing (G4–G17, G5–G18). (**F**) Unusual guanine–guanine (G–G) pairing (G15–G9) upon KR12A interaction with Myc22.

Opposed to KR12C-Myc22 association, we observed the evolution of 1D proton signals between 12.5 and 14 ppm in KR12A-Myc22 complex, which reflected conformational change in the quadruplex upon KR12A titration (Figure 5A). Upon further titration at 12:1 molar ratio (KR12A:Myc22) the imino signals between 10 to 12 ppm completely disappeared whereas the signals at 12.5–14 ppm sustained and were significantly broadened (Figure 5B). This observation validated our earlier finding, which showed significant depletion (∼30°C) of the melting temperature of Pu27 and Myc22 upon KR12A binding. Based on these experimental observations, we conducted constrained docking of Myc22 and KR12A and further simulated the complex for 100 ns, which clearly detected the perturbation of the quartets upon KR12A interaction (Figure 5C). We mapped the binding interface at atomic level, which evidenced strong electrostatic interactions between R6 and G20, K8 and G18 (Figure 5D). In contrast to KR12C, KR12A preferred to target the 3′-termini of Myc22 quadruplex, which was further substantiated by significant broadening and downfield shift of the proton resonances near the binding site (Figure 5B–D). This denotes greater exposure of the imino protons to the solvent, which sequestered the stable Hoogsteen bonding and resulted in conformational fluctuation. We also observed formation of reverse Watson–Crick interaction between G4-G17, G5-G18 and G14-G18. Furthermore, G18 was involved to maintain an equilibrium between G5 and G14 via Hoogsteen and Reverse Watson–Crick interactions respectively. We observed Guanine-guanine (G–G) pairing via Hoogsteen interaction (G13–G17, G15–G19, G4–G13 and G14–G18) and Reverse Watson–Crick interactions, which contributed to the signals between 12.5 and 13 ppm (Figure 5E). To our surprise, we found an unusual G–G interaction involving N7-imino(N1(H1)) and amino(N2(H2))-O6, which occurred in 80% of the MD trajectories (Figure 5F). These interactions might have further caused downfield shift of the 1D signal at 13.5 ppm due to enhanced solvent accessibility of the base pair.

### Peptides regulate the occupancy of Sp1, NM23-H2 and Nucleolin at *c-MYC* quadruplex motif to downregulate *c-MYC* transcription

**Figure 6.**
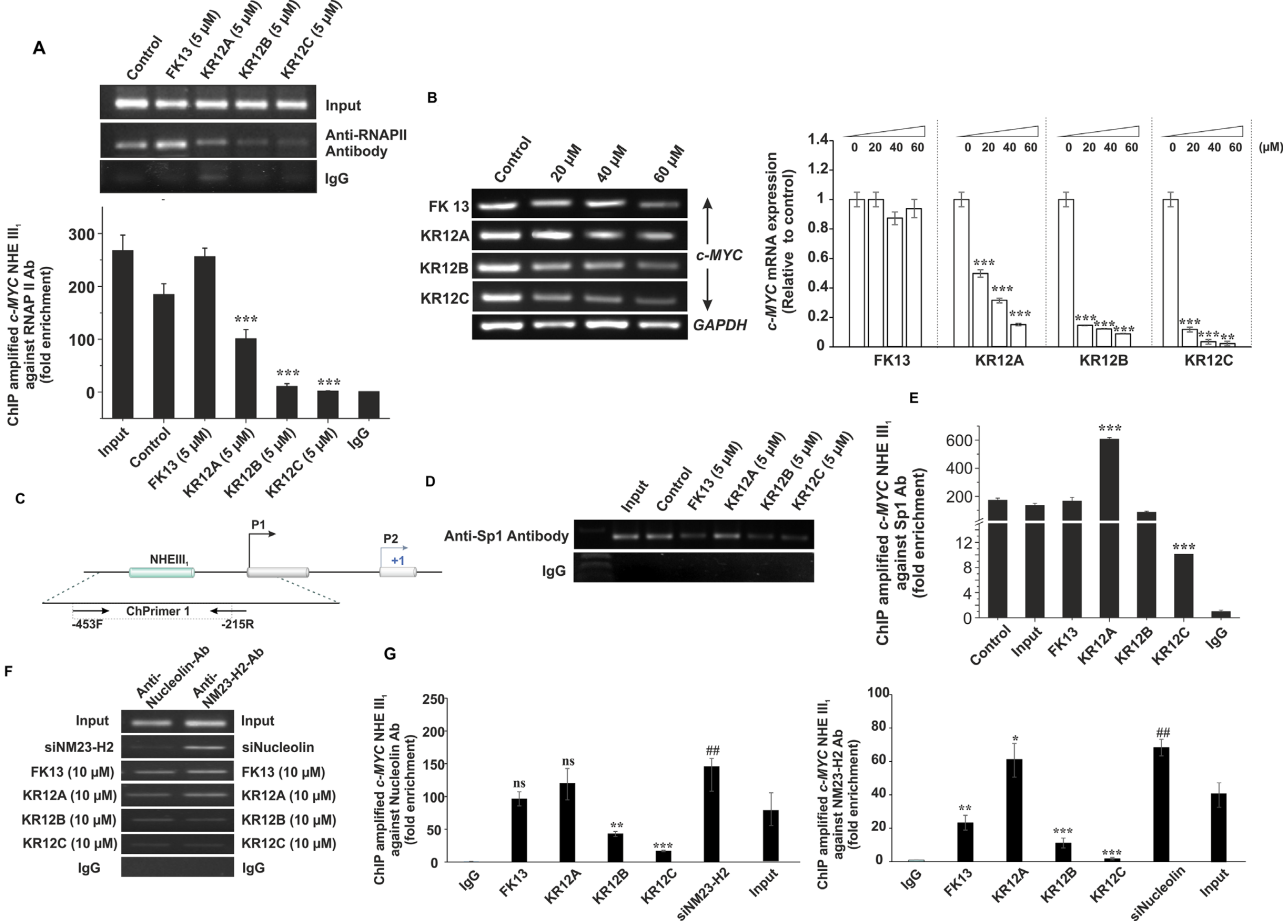
Differential recruitment of NM23-H2 and Nucleolin at Pu27 triggered by the peptides. (**A**) ChIP results using RNA polymerase II (RNAPII) specific antibody. RNAPII recruitment at *c-MYC* promoter shown under the treatment of FK13, KR12A, KR12B, and KR12C. Quantification of the binding of RNAPII at *c-MYC* promoter by fold enrichment in binding compared with the IgG control based on quantitative real-time PCR. (**B**) Expression profile of *c-MYC* transcripts from P_1_ promoter upon the treatment of FK13, KR12A, KR12B and KR12C with increasing concentrations. Quantification of the transcripts’ level relative to the control by qPCR analyses. Error bars represent mean ± SE (*N* = 3). Statistical differences are determined compared to the control by two-tailed Student's *t* test (*P < 0.05, **P < 0.01, ***P < 0.001). (**C**) Schematic representation of *c-MYC* promoter region. Transcription initiation sites P1 and P2, Nuclease Hypersensitive Element (NHE III_1_) having the quadruplex-forming motif are shown. Position of the ChIP primers used in semi-quantitative and Real time PCR reactions following immunoprecipitation is indicated. (**D**) ChIP results using Sp1 specific antibody. Sp1 recruitment at the NHE III_1_ shown under the treatment of FK13, KR12A, KR12B and KR12C. (**E**) Quantification of the binding of Sp1 at NHE III_1_ by fold enrichment in binding compared with the IgG control based on quantitative real-time PCR (**F**) ChIP results using NM23-H2 and Nucleolin specific antibody. NM23-H2 and nucleolin enrichment at NHE III_1_ shown under siRNA knockdown of Nucleolin, and NM23-H2 respectively and peptide treatment. (**G**) Quantification of the binding of NM23-H2 and Nucleolin at NHE III_1_ by fold enrichment in binding compared with the IgG control based on quantitative real-time PCR. Error bars represent mean ± SE (*N* = 4). Statistical differences are determined by one-way ANOVA followed by Tukey–Kramer test (*P < 0.05, **P < 0.01, ***P < 0.001).

We have earlier witnessed that FK13, KR12A, KR12B and KR12C inhibited *c-MYC* promoter activation in MCF-7 cells albeit showing differential affinity or selectivity to Pu27 and other quadruplex topologies. To substantiate these results, we determined the differences in the occupancy of RNA Polymerase II (RNAPII) across the promoter upon peptide treatment and estimated the level of *c-MYC* transcripts in MCF-7 cells upon increasing peptide concentrations (Figure 6A and B). Consistent with the earlier results, KR12B and KR12C significantly depleted RNAPII recruitment in the promoter and repressed *c-MYC* transcription by ∼5-fold. Interestingly, FK13 did not significantly altered RNAPII occupancy across *c-MYC* promoter and did not downregulate *c-MYC* transcription despite having an inverse role in *c-MYC* promoter repression in MCF-7 cells. KR12A downregulated *c-MYC* transcription in a dose-dependent manner and also depleted RNAPII occupancy in the promoter albeit having poor affinity on the quadruplex (Figure 6A and B). However, the magnitude of transcription repression by KR12A is highly reduced compared to KR12C and KR12B. This might be due to their non-specific binding to other oncogenic quadruplexes as shown in the luciferase assays and their associated off-target effects. FK13 and KR12A also abrogated the transcriptions of other oncogenes (*BCL-2, VEGF-A, KRAS*), which are known to regulate *c-MYC* transcription through an intricate network (Supplementary Figure S11). Therefore, the inverse effects of FK13 and KR12A in *c-MYC* promoter inhibition might be integrated with their interferences in the upstream signalling pathways, which compromised the stable complexation with *c-MYC* quadruplex in the cells.

To gain a deeper insight into the role of peptides in *c-MYC* transcription, we monitored the occupancy of Sp1, NM23-H2 (Nucleoside diphosphate kinase), and Nucleolin in NHE III_1_. Earlier reports claim that Sp1-induced negative super helicity declines nucleolin occupancy at NHE III_1_ driving an increased *c-MYC* transcription (86). There are reports that NM23-H2 disentangles *c-MYC* quadruplex to enhance the rate of transcription while Nucleolin, which attenuates the promoter activation by stabilizing Pu27, antagonizes this effect (87,88). In this study, we conducted Chromatin immunoprecipitation studies on a region encompassing *c-MYC*-NHE III_1_ and monitored the changes in promoter occupancy of Sp1, NM23-H2 and Nucleolin upon peptide treatment (Figure 6C). We observed that KR12C depleted the enrichment of Sp1, NM23-H2 and nucleolin at *c-MYC*-NHE III_1_ while FK13 exhibited no role in the recruitment or depletion of these transcription factors at Pu27 (Figure 6D–G). Since Sp1 and NM23-H2 are the positive regulators of *c-MYC* transcription, a significant depletion in their recruitment across NHE III_1_ by KR12C accounted for its selective binding with *c-MYC* quadruplex under cellular microenvironment and its inverse effect in *c-MYC* transcription regulation. KR12B had no role in Sp1 occupancy but depleted the abundance of NM23-H2 and nucleolin (Figure 6D–G). Therefore, the inhibitory effects of KR12B in *c-MYC* transcription and promoter activation were associated with its non-specific interactions with other oncogenic quadruplexes and off-target effects because *c-MYC* transcription is stringently regulated by cellular crosstalk involving the congregation of different transcription factors across the P_1_/P_2_ promoter (Supplementary Figure S11). Smits *et al.* showed that the decreased expression of miR-125b by VEGF results in an upregulation of MAZ (Myc-associated zinc finger protein) expression, which specifically binds to a GA box sequence (GGGAGGG) in *c-MYC-*P_2_ promoter to regulate *c-MYC* transcription (88,89). Since FK13 and KR12B strongly inhibited *VEGF-A* promoter activity and its transcription, the negative effects of these peptides on *c-MYC* promoter might be due to their non-specific targeting at VEGF-A quadruplexes. KR12A did not significantly affect Nucleolin recruitment but slightly enriched NM23-H2 and Sp1 occupancy (Figure 6D–G). Since KR12A destabilized the quartets facilitating the passage of G-rich motif into hairpin-loop like structure, it favoured the recruitment of NM23-H2 at Pu27. However, KR12A did not unfold *c-MYC* quadruplex in a transcriptionally active form. Instead, it destabilized the tetrad core allowing the formation of reverse Watson–Crick G–G pairs and Hoogsteen base pairs; i.e., significantly different from the quartet core observed in the canonical G-quadruplexes. This type of secondary structures were highly flexible and less stable opposed to the quadruplex structures as observed in the CD melting experiments. However, such conformation can potentially act as a roadblock for RNAPII as evidenced by our ChIP results. This is a plausible explanation why KR12A depleted the abundance of RNAPII across NHE III_1_ which resulted in a downregulation of *c-MYC* transcription in MCF-7 cells. However, we observed that KR12A non-specifically interacted with other oncogenic quadruplexes and repressed their transcription like FK13 and KR12B. Therefore, KR12A-mediated off-target effects and its involvement in the upstream signalling cascade could also affect *c-MYC* transcription. Wierstra I *et al.* demonstrated that FOXM1c (Forkhead Box M1) transactivates P_1_/P_2_ promoter synergistically with Sp1 (86,87). KR12A was also found to show poor affinity to *c-MYC* quadruplex and enhance Sp1 recruitment in NHE III_1_, which further corroborated why it could not strongly abrogate *c-MYC* transcription compared to KR12B and KR12C.

### KR12C drives apoptosis signalling cascade via E2F-1-VEGF-A-BCL-2 axis in MCF-7 cells

**Figure 7.**
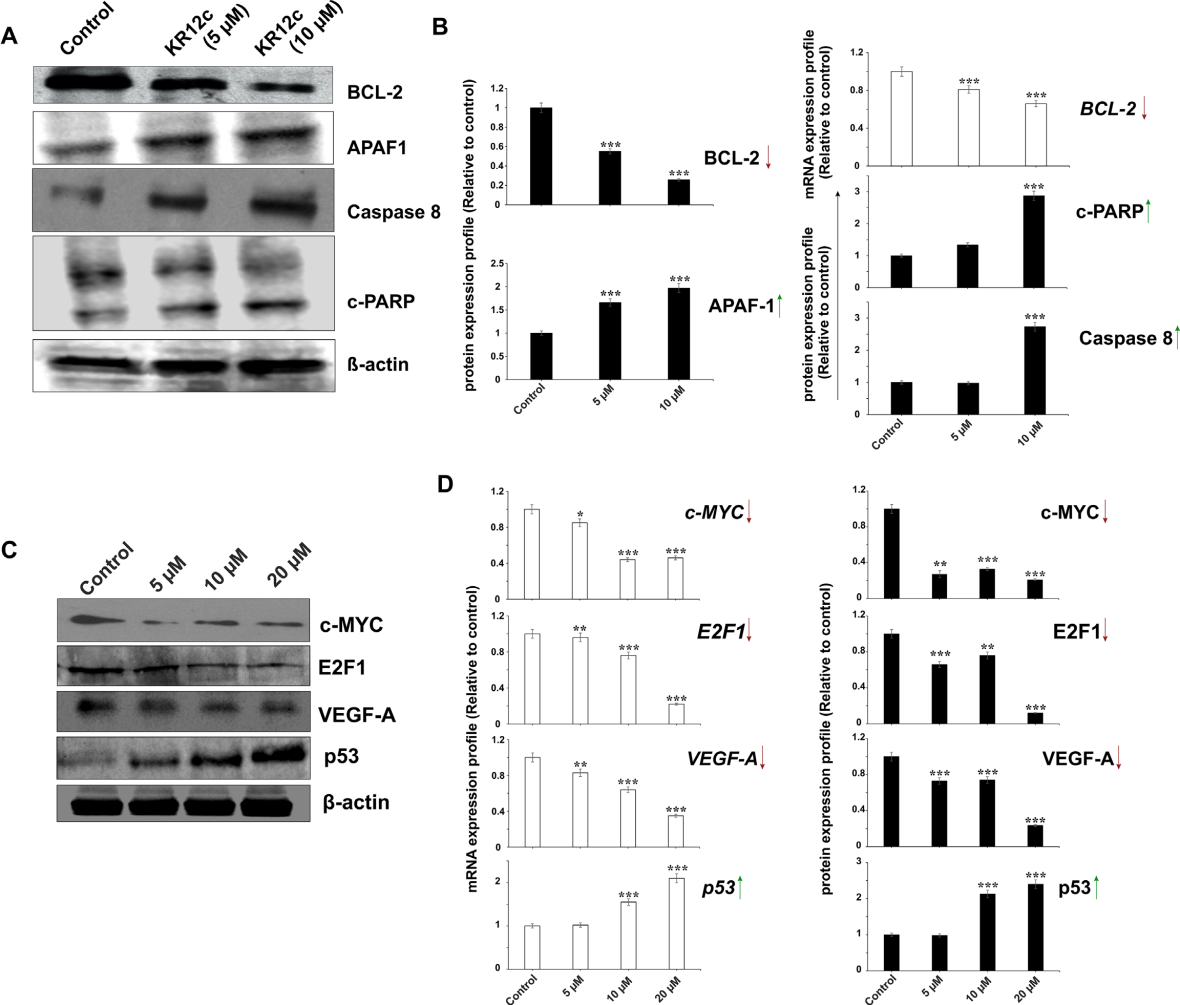
Selective arresting of *c-MYC* quadruplex by KR12C induce apoptotic signalling in cancer cells. (**A**) Western blot analyses of the major apoptotic markers (BCL-2, APAF-1, caspase 8 and PARP). (**B**) Estimation of protein expression level of the apoptotic markers by semi-densitometric analyses. Estimation of *BCL-2* transcripts by real time PCR analyses at 5 and 10 μM KR12C concentration. (**C**) Western blot analyses of other proteins associated with the signalling cascade (c-MYC, E2F-1, VEGF-A and P53). (**D**) Quatification of protein and mRNA expression level of c-MYC, E2F-1, VEGF-A and P53 by semi-densitometric analyses and real time PCR respectively. Arrow pointing upwards and downwards denote the upregulation of the respective protein and mRNA expression level. Error bars represent mean ± SE (*N* = 3). Statistical differences are determined compared to the control by two-tailed Student's *t* test (*P < 0.05, **P < 0.01, ***P < 0.001).

**Figure 8.**
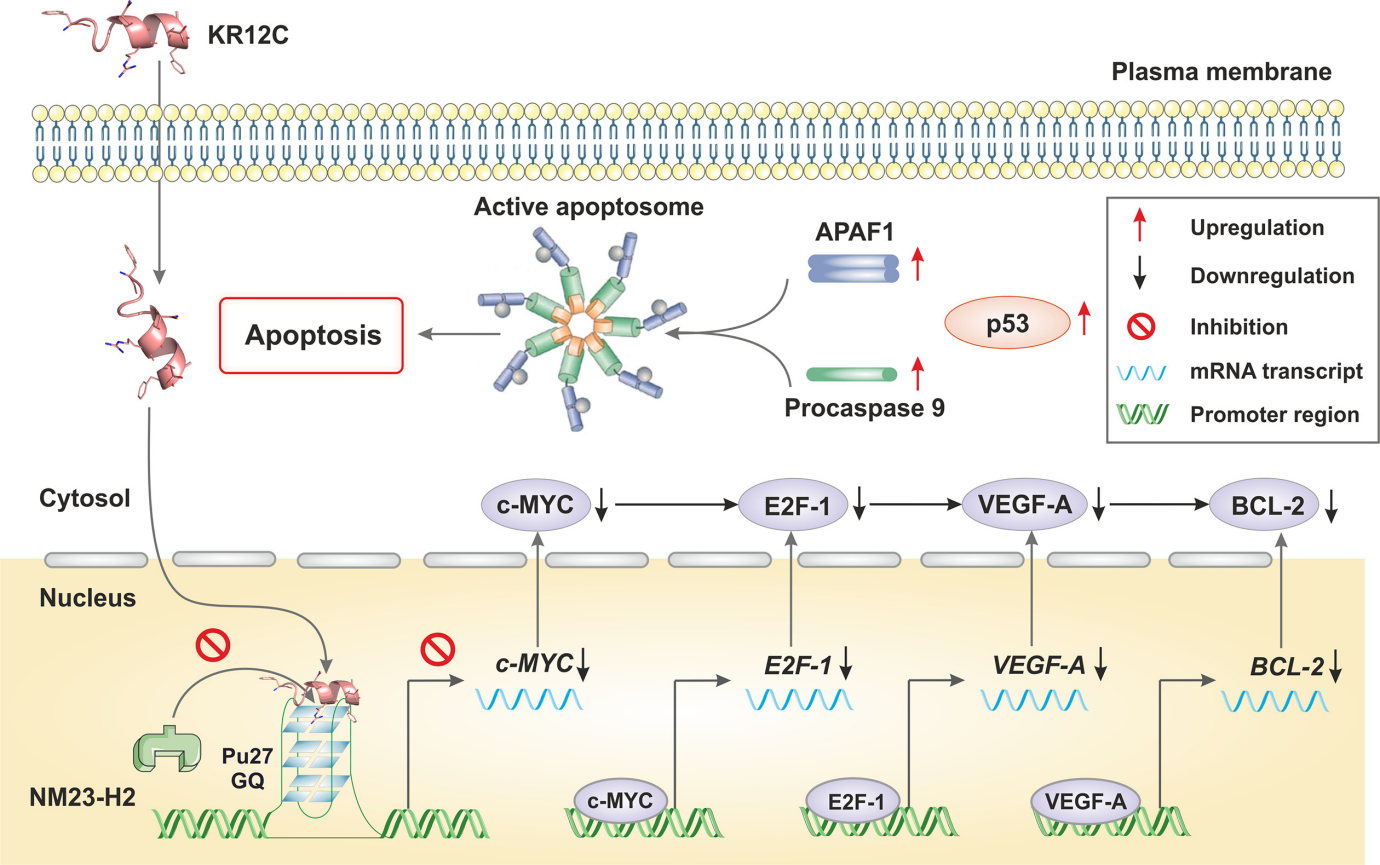
Schematic representation showing selective quadruplex interaction at *c-MYC* promoter by KR12C promotes apoptotic signalling in VEGF-A-BCL-2 axis in MCF-7 cells.

So far, we observed that KR12C is the best peptide candidate that selectively repressed *c-MYC* promoter activation in cancer cells. Earlier reports showed its ability to trigger apoptosis in cancer MCF-7 cells (89). We further validated these results through monitoring the expression profiles of different oncogenes and apoptosis markers in order to deduce the mechanism how G-quadruplex stabilization at *c-MYC* promoter triggered selective apoptosis in cancer cells. We observed significant upregulation of p53, cleaved PARP (poly (ADP-ribose) polymerase), caspase 8 (cysteine-aspartic proteases), and APAF-1 (apoptotic protease activating factor-1), which had been well studied to positively regulate the apoptotic cascade (Figure 7A and C). To our surprise, we observed that KR12C suppressed BCL-2 expression at both transcription and translation level despite having no regulation upon its promoter under the control of *BCL-2* quadruplex (Figure 7A and B). This result envisaged that quadruplex stabilization at *c-MYC* promoter by KR12C turned on a specific downstream cascade as to which the expression of *BCL-2* was depleted to such an extent to promote apoptosis in MCF-7 cells. To unravel this downstream pathway, we checked the expression of E2F-1 and VEGF-A at transcription and protein level, which were the upstream oncogenes of BCL-2 and significantly regulate its transcription. Compelling evidences are available about the crosstalk between c-MYC and VEGF-A and their positive role and overexpression in breast cancer progression (90). VEGF-A is also found to stimulate the growth of breast cancer cells in a c-MYC dependent manner in xenograft models (91). c-MYC binds to an E-box motif at *VEGF-A* promoter which positively regulates its transcription in cancer cells. Another study revealed that c-MYC activates miR-9, which in turn elevates VEGF-A expression via β-catenin signalling pathway (92). c-MYC also upregulates E2F-1 transcription (93), which is shown to positively regulate VEGF-A expression (94,95). We observed that VEGF-A and E2F-1 were significantly suppressed due to repressed transcription and lower abundance of c-MYC (Figure 7C and D). Furthermore, VEGF-A promotes survival in both tumor and endothelial cells and prevents apoptosis via inducing BCL-2. This observation was strengthened by the treatment with recombinant VEGF (rVEGF) which partially rescued the inhibitory effects of KR12C, highlighting the role of VEGF-A in this context (Supplementary Figures S12A–C). Our data complied with the available reports and showed that KR12C is arresting *c-MYC* transcription leading to decreased survival and apoptosis via perturbing the VEGF-BCL-2 axis (Figure 8).

## DISCUSSION

Selective targeting of the oncogenic quadruplexes by therapeutic peptides raised great expectations for future pharmacological and clinical applications due to their variability and plasticity of the three-dimensional structures and potential to mimic protein–quadruplex interactions, which manifest into enhanced intracellular quadruplex affinity and selective modulation of the oncogenic functions with minimal off-target effects in cancer cells. Since native quadruplex structures upstream *c-MYC* promoter exhibit structural heterogeneity allowing optimal kinetic advantage for the shuffling of the transcription factors, small molecules often fail to conform to the flexible conformation of the target quadruplex resulting non-specific binding to other oncogenic quadruplexes in the cells. Therefore, the clinical pharmacology of quadruplex-based treatment is still in its dawn albeit offering a promising avenue for anti-cancer therapies.

In this study, we designed six peptides by pruning the quadruplex-binding domain of a naturally occurring human cathelicidin peptide (LL37) and substituted the amino acids in the peptide sequences with the rationale of selective anti-tumor properties and enhanced quadruplex affinity. First, we conducted dual-luciferase assays for rapid screening of the synthetic peptides to identify the candidates having preferential targeting at *c-MYC* quadruplex over other intracellular quadruplexes of different folding topologies. We observed that KR12A, KR12B and FK13 exhibit non-specific targeting at oncogenic quadruplexes while KR12C specifically suppressed *c-MYC* promoter activation via stabilized interaction with the wild-type quadruplex upstream its promoter region. We analysed differential selectivity of the peptides for *c-MYC* quadruplexes by thermodynamic profiling of their binding reactions in ITC experiments, which again supported that KR12C had maximum binding affinity for *c-MYC* quadruplex and its isomers (Myc22 and Pu19) while rest of the peptides showed poor and moderate binding to the quadruplex element. The binding affinity of KR12C for *c-MYC* quadruplex is enhanced by ∼8 fold under macromolecular crowding of MCF-7 nuclear extract, which suggests that the volume exclusion by the increased chemical activity of the macromolecules in nuclear extract increases binding affinity by favouring the association of KR12C and *c-MYC* quadruplex. This underscores intracellular selectivity of KR12C for *c-MYC* quadruplexes. We correlated the differences in the magnitude of binding affinities with specific non-covalent interresidual interactions by *in silico* modelling of the peptide-quadruplex complexes and their simulation for 100 ns in explicit solvent. We observed that the electrostatic interactions by positively charged amino acids (Arginine and Lysine) prevailed in the sugar-phosphate backbone of the quadruplexes. However, the binding affinity and selectivity is driven by differential helical properties of the peptides, which alleviate CH_3_-π interaction with the tetrad plane and Hoogsteen hydrogen bonding between the adenine residues (A6 and A15). These interactions are pronounced in KR12C and Pu27, which accounts for its selective interaction with *c-MYC* quadruplex.

In most cases, ligands with aromatic/pseudoaromatic π-delocalized system are developed to bind *c-MYC* quadruplex *via* external π–π stacking. These compounds generally suffer from non-specific target recognition resulting toxic off-target effects (e.g. porphyrine derivative TmPyP4(41), perylene derivative PIPER (42,43), quindoline derivative SYUIQ-05(44)) since around 3,70,000 nucleic acid sequences in human genome have the propensity to fold into quadruplex structures and maximum of the lead compounds offer G-quadruplexe-to-duplex selectivity through stacking interaction. Therefore, the loop characteristics of *c-MYC* quadruplex should definitely be considered (*i.e.*, the length and folding pattern of the loops; (e.g. diagonal or propeller-loop), the groove width, torsion angles of glycosidic bonds, strand polarity) for designing a specific drug candidate. For example, *c-MYC* and *c-KIT* quadruplexes allow ligand accessibility at 5′ and 3′ terminal quartets, whereas telomeric and *BCL-2* quadruplexes (1:13:1 isomer (Pu39WT) and 1:1:11:1 isomers (P1G4)) restrict this access of due to steric clashes at the terminal quartet regions. The groove width of the *BCL-2* quadruplex is also significantly different from others. (e.g. groove widths of *BCL-2* quadruplex (PDB: 2F8U) are 10.3, 6.4, 17.5 and 7.7 Å, in *c-MYC* (PDB: 1XAV) the groove widths are 9.7, 14.9, 12.7, 11.9 Å). Considering these unique properties of quadruplex loops in *c-MYC* quadruplex, we put more emphasis on selective interaction in quadruplex loops by the peptides. In Pu27, the planar conformation of A6 and A15, stacked over the first quartet generates a specific binding pocket for KR12C at the 5′-terminus groove of *c-MYC* quadruplex. The peptide, KR12C makes highly stable Coulombic interactions with the sugar-phosphate backbone at the 5′-propeller loop to strengthen A5–A16 Reverse Watson interaction. This interaction between A15-A6 is further stabilized by L11 and I7 via CH_3_–π interactions, which gives a bona fide advantage to maintain the integrity of the quartet core of *c-MYC* quadruplex without external stacking interaction, opposed to other known quadruplex-targeting small compounds. Solution NMR structure calculations were also consistent with our premonitions as KR12C is preferentially targeted at the vicinity of the 5′-tetrad plane of Myc22 (the restricted conformer of Pu27), which in turn stabilized the 5′-propeller loop by a network of electrostatic interactions supported by strong, moderate, and weak interresidual crosspeak intensities at the proximity of the quartet and sugar backbone. These interactions not only confer quadruplex-to-duplex selectivity but also specifically target *c-MYC* quadruplex under cellular microenvironment. This observation is further supported by a sharp rise (∼8-fold) in the binding affinity (*K*_d_ = 901 nM) of KR12C for Pu27 under macromolecular crowding. In contrast, we witnessed the unfolding of *c-MYC* quadruplex by KR12A that resulted formation of reverse Watson–Crick interactions and guanine–guanine Hoogsteen pairing at the G-rich motif. This accounts for the depletion of quadruplex thermostability and enhancement of the entropy of unfolding.

Our investigations further perpetuate with the role of peptides in regulating the promoter occupancy of transcription factors (NM23-H2 and Nucleolin) at *c-MYC* quadruplex. We observed that KR12B and KR12C inhibit the recruitment of NM23-H2, a protein known to upregulate the transcription by unfolding the quadruplex into single-stranded motif. However, KR12A increases NM23-H2 enrichment in the quadruplex as similar as the control and provides minimal repression in the transcription. Therefore, the flexible skeleton of KR12C not only adapts an optimized posture upon Pu27 binding, but its selective contacts at its loop region induces allosteric changes in the receptor such that NM23-H2 no longer binds to the native quadruplex. This led us further investigate how selective stabilization of Pu27 by KR12C propagates downstream signalling cascade to promote apoptosis in cancer cells. We deduced that selective arresting of *c-MYC* promoter via stabilizing G-quadruplex activates a unique crosstalk with E2F-1 and VEGF-A, that concomitantly reduces BCL-2 expression triggering apoptosis in cancer cells. Our study improves the insight of increasing the selectivity of the peptides for *c-MYC* quadruplex at the atomic level and opens a new paradigm for next generation quadruplex-targeting peptides with higher therapeutic index and minimal off-target effects in the cells.

## Supplementary Material

Supplementary Data
