# Supplementary material for: Site-specific amino acid substitution in dodecameric peptides determines the stability and unfolding of c-MYC quadruplex promoting apoptosis in cancer cells
Source: Nucleic Acids Res. 2018 Sep 17;46(19):9932–50. doi: 10.1093/nar/gky824 (PMC6212778; doi:10.1093/nar/gky824)
Supplement: Supplementary Data [file gky824_supplemental_files.pdf]

# Supplementary Material

## Site-specific Amino Acid Substitution in Dodecameric Peptides Dictates the Stability and Unfolding of c-MYC Quadruplex and Promote Apoptosis in Cancer Cells

Pallabi Sengupta[1†], Nilanjan Banerjee[1†], Tanaya Roychowdhury[2], Anindya Dutta[1], Samit Chattopadhyay[2], and Subhrangsu Chatterjee[1*]

[1] Department of Biophysics, Bose Institute, P-1/12 CIT Scheme VII (M), Kolkata 700054, India

[2] Cancer Biology and Inflammatory Disorder Division, CSIR-Indian Institute of Chemical Biology, 4, Raja S. C. Mullick Road, Kolkata-700032, India

† Both Author contributed equally

*To whom correspondence should be addressed. Tel: (033) – 2569-3340; Email: subhrangsu@gmail.com

### Table of contents

**1. Experimental Section:**

**1.1. Cell and Molecular Biology**

**1.1.1.** Construction of the reporter luciferase vectors 2
**1.1.2.** Transfection of luciferase constructs and siRNA into MCF-7 cells 2
**1.1.3.** Real time PCR and Semi-quantitative Reverse Transcription (RT)-PCR 2

**1.2. *In silico* experiments and biophysical studies**

**1.2.1.** Bioinformatic studies 3
**1.2.2.** Preparation of oligonucleotide sequences 3
**1.2.3.** Peptide synthesis 3
**1.2.4.** Circular dichroism 3
**1.2.5.** Isothermal titration calorimetry 4
**1.2.6.** Molecular Docking 5
**1.2.7.** Molecular dynamics and simulation 5
**1.2.8.** NMR spectroscopy 6

**2. Tables** **6 – 10**

**3. Supplementary Figures Legends** **11 – 12**

**4. Sequence information of cloning** **13 -14**

**5. References** **29**

# 1. Experimental Section:

## 1.1. Cell and Molecular Biology:

**1.1.1. Construction of the reporter luciferase vectors:** Luciferase constructs having oncogene promoters (*BCL-2, KRAS,* and *VEGF-A*) with and without the G-quadruplex scaffolds are prepared following the protocol published earlier(1). *c-MYC* promoter sequences ($P_1$ and $P_2$) with or without upstream quadruplex-forming elements (wild-type-Pu27, mutants-Myc22 and Pu19) are cloned into a promoter-less pGL4.72 [*hRlucCP*] luciferase vector at KpnI and HindIII restriction sites. The reporter vector encodes a luciferase reporter gene, *hRlucCP* (*Renilla reniformis*) engineered with lesser cryptic DNA regulatory sequences and a synthetic poly(A) signal pause site upstream the Multiple cloning site (MCS) to minimize spurious transcriptions. The synthetic reporter gene, *hRlucCP* is followed by two protein destabilizing sequences, *hCL1* and *hPEST* to enhance the rate of reporter response with a higher magnitude to the changes in the transcription activity and further codon optimized for mammalian expression(2,3). The 503 bp (base pair) long *in vitro* custom synthesized *c-MYC* promoter construct (Pu27C) has been procured from Biobharati India Pvt. Ltd. and sub-cloned into pGL4.72 [*hRlucCP*] at the same restriction sites. Pu27C construct encompasses -467 to +36 bases of *c-MYC* promoter, which harbour both $P_1$ and $P_2$ promoters along with the 27 bp stretched wild-type quadruplex forming motif (Pu27) within NHE $III_1$ (**Figure 1A**)(4). The quadruplex–deficient *c-MYC* promoter sequence (144 bp) is PCR (Polymerase chain reaction) amplified from the genomic DNA, isolated from MCF-7 cell line using primers, -107GQ-nullFP (containing the KpnI site) and GQ-nullRP (containing the HindIII site)[1*]. The PCR products are purified using QIAquick Gel Extraction Kit (Qiagen), cut with KpnI-HF® (NEB) and HindIII-HF® (NEB) enzymes and further purified with QIAquick PCR Purification Kit (Qiagen). Then, the double-digested PCR-products are cloned into same restriction sites of pGL4.72[*hRlucCP*] vector using T4 DNA ligase (NEB). This reporter plasmid is designated as 'GQ-null' (quadruplex-deficient), which includes bases between -107 and +36 of the promoter while eliminates out the quadruplex-forming motif in NHE $III_1$, situated upstream P1 promoter (**Figure 1A**). The mutant constructs (Pu19C and Myc22C), which are overlapped into different regions of wild-type NHE $III_1$ (Pu27) are further created over Pu27C template using overlap-extension PCR techniques (**Supplementary Figure S2**). For Pu19C construct, one-step overlap extension PCR has been employed using mutagenic primers (**Supplementary Table S7**) whereas for Myc22C, two-step overlap PCR are conducted. The first round PCR reaction is carried out using primers -335FP and Pu24RP while the second round reaction is separately performed with primers -319mFP and Pu25RP. All the PCR products are purified and combined to use as templates for the final extension using -467wtFP and wtRP primers. The resultant product is cleaned, cleaved by restriction enzymes and cloned as explained above. Successful cloning of different inserts are validated by sequencing (**Supplementary section 4. Sequence details**).

**1.1.2. Transfection of luciferase constructs in MCF-7 cells:** Luciferase constructs having oncogene promoters (*c-MYC, BCL-2, KRAS,* and *VEGF-A*) with and without the G-quadruplex scaffolds are transformed into One Shot® Mach1™ T1 competent *E. coli* cells (Invitrogen) to amplify the reporter plasmids. Transformed cells are harvested into Luria broth (LB) agar plates having 100 μg/ml Ampicillin and incubated at 37°C for 8-9 hours. Singular colony from each plate is inoculated into Ampicillin-supplemented LB media for 10-12 hours at 37°C and plasmids are isolated using QIAprep Spin Miniprep Kit (Qiagen) for transfection assays. MCF-7 cells are sub-cultured into 24-well microtiter plates at a cell density of $1\times10^5$ cells per well. 500 ng of the reporter constructs (with and/or without quadruplex motifs) driving the expression of Renilla luciferase are co-transfected with 50 ng of pGL3-control vectors (Promega) (used as internal control), encoding firefly luciferase into MCF-7 cells using Lipofectamine® 3000 transfection reagent (Thermo-Fisher Scientific) as per manufacturer's protocol. After 24 hours of transfection, cells are treated with synthetic peptides (FK13, KR12A, KR12B, KR12C, KR12D, and KR12E) at an increasing concentration gradient.

**1.1.3. Real time PCR and Semi-quantitative Reverse Transcription (RT)-PCR:** Real time polymerase chain reactions are conducted to elucidate the role of peptides in the downregulation of *c-MYC* expression profiles at the transcription level. We have also evaluated the mRNA expression profiles of other genes (*BCL-2*, *VEGF*-A, *KRAS, p53,* and *E2F-1*) to compare the magnitude of transcription downregulation upon peptide treatment and further determine the signaling cascade due to endogenous peptide-quadruplex association. MCF-7 cells are sub-cultured into 6-well microtiter plates at a density of $1 \times 10^6$ cells per well. Cells are treated with different concentrations of peptides (FK13, KR12A, KR12B, and KR12C) for 24 hours.

[1*] FP: Forward Primer; RP: Reverse Primer

Then, total RNA is isolated from both untreated and treated cells using TRIzol method (Invitrogen) as per manufacturer's instructions. 2 μg of total RNA is processed for cDNA synthesis and reverse transcribed using a Super MuLV RT Kit (Biobharati Life Sciences Pvt. Ltd.). Real time PCR is performed using Maxima SYBR Green/ROX qPCR Master Mix (2X) (Thermo-Scientific) as per manufacturer's protocol. Housekeeping gene, *GAPDH* is used as an internal control to normalize the variability in *c-MYC* mRNA expression levels. Relative fold change in gene expression is measured by $2^{-\Delta\Delta C_T}$ method. First, $\Delta C_T$ is computed that dictates the difference in threshold cycle between the target and housekeeping genes,

$$\Delta C_T = C_{T(Target)} - C_{T(GAPDH)} \ldots\ldots\ldots\ldots\ldots\ldots\ldots\ldots \textbf{Eq. (1)}$$

Then, the difference between $\Delta C_T$ values of untreated and treated samples are quantitated giving the value of $\Delta\Delta C_T$.

$$\Delta\Delta C_T = \Delta C_{T(Untreated)} - \Delta C_{T(Treated)} \ldots\ldots\ldots\ldots\ldots\ldots\ldots\ldots \textbf{Eq. (2)}$$

Fold changes in the gene expression level relative to the calibrator is determined by the formula:

$$R = 2^{-\Delta\Delta C_T} \ldots\ldots\ldots\ldots\ldots\ldots \textbf{Eq. (3)}$$

In semi-quantitative Reverse Transcription PCR, Polymerase chain reactions are carried out with a 5 min denaturation step at $94^0$C followed by 45 amplification cycles of denaturation at $94^0$C for 30 s, annealing at specific temperature for 45 s, and extension at $72^0$C for 45 s. Final PCR products are run on 1.5% agarose gels along with a 100 bp DNA ladder (Thermo Scientific). Semi-quantitative densitometric analyses are performed to determine the relative mRNA expression levels. PCR primers were designed using Primer-BLAST, NCBI, and analyzed in OligoAnalyser 3.1-IDT (**Supplementary Table S2**).

## 1.2. *In silico* studies and biophysical experiments:

**1.2.1. Bioinformatic studies:** Anti-CP prediction server is used to estimate the net charge distribution and anti-cancer properties of the peptides used in the study. Cell PPD server is utilized to predict the cell membrane permeability of the peptides. ToxinPred web-server is employed for predict potential cellular toxic effects of the peptides.

**1.2.2. Preparation of oligonucleonucleotide sequences:** The putative wild-type (Pu27) and truncated quadruplex–forming oligonucleotide sequences (Myc22 and Pu19) residing at the Nuclease Hypersensitive Element $III_1$ upstream *c-MYC*-$P_1$, the native quadruplex-forming sequences in the upstream promoter region of *BCL-2* (Pu30), *KRAS* (Pu32), *VEGF-A* (Pu22), and Telomere region (Tel26) are obtained from Eurofins Genomics India Pvt Ltd. in lyophilized form (**Table 2 and Supplementary Table S1**). These sequences are reconstituted in 10 mM Potassium phosphate buffer (10 mM $K_2HPO_4$ + 10 mM $KH_2PO_4$) supplemented with 0.1 M KCl and 1 mM Ethylenediaminetetraacetic acid (EDTA) at pH 7.0. The sequences are annealed by heating at $95^0$C for 5 minutes followed by gradual cooling to room temperature overnight to alleviate the formation of G-quadruplex structures *in vitro*.

**1.2.3. Preparation and purification of the peptides:** Peptides are synthesized in Solid phase Peptide synthesizer (Aapptec Endeavor 90) following Fmoc chemistry. 14 mg of Rink amide MBHA resin is taken into peptide vessel and allowed to swell overnight in DMF solvent. Fmoc-protected amino acids are sequentially coupled followed by fmoc deprotection using 20% piperidine solution in peptide synthesizer. Coupling and deprotection steps are maintained for sixty and forty minutes respectively. DIPEA and PyBOP are used as activator base and activator respectively and DMF is used as solvent. Peptide attached resin is washed by DMF solvent and cleaved by standard resin cleavage cocktail solution containing 87.5% trifluoroacetic acid (TFA), 5% milli Q water, 2.5% TIS, 2.5% Anisol, and 2.5% phenol. Resin attached peptide is kept for two hours containing the peptide cleavage solution and TFA is subsequently removed from the filtrate by rotatory evaporator. The filtrate is added gradually to cold diethyl ether solvent to ensure complete precipitation and then is separated by centrifugation. Peptides are purified using reverse phase HPLC system with Phenomenix C18 column (dimension 250 × 10 mm, pore size 100 Å, particle size 5 μm) by linear gradient elution having dual solvent system (Water and Acetonitrile) containing 0.1 % TFA. Peptide masses are confirmed by MALDI-TOF mass spectroscopy (Bruker).

**1.2.4. Circular dichroism (CD):** Circular dichroism experiments are carried out in JASCO-J815 CD spectrometer (Jasco International Co., Ltd.) equipped with a peltier cell holder and a temperature controller CDF-426 L at $25^{\circ}$C. The putative

quadruplexes (**Table 2**) are diluted to 10 μM in the annealing buffer and peptides (FK13, KR12A, KR12B, KR12C, KR12D, and KR12E) is subsequently titrated in an increasing concentration gradient in the quadruplexes. Each scan is recorded after 5 minutes of peptide addition to allow complex formation and equilibration. Samples are scanned over the wavelength range of 320 – 210 nm having a scanning speed of 100 nm/min. Data points are acquired at an interval of 1 nm and averaged over three repetitive scanning accumulations. For each measurement, 1 mm path length quartz cuvette (Starna Scientific Ltd) is used having an active reaction volume of 350 μl. The digital integration time and bandwidth are 2 s and 1 nm respectively. Molar ellipticity values for all scans are corrected for solvent contribution.

To decipher the role of peptides in G-quadruplex stabilization, temperature induced melting experiments are conducted for free and peptide-bound complexes using the aforementioned parameters. For thermal scans, the samples are heated from 20° – 95°C using Jasco programmable Peltier element. Temperature gradient and delay time are defined at 2.5°C/min and 150 s respectively considering the observed independence of thermal transitions of the heating/cooling rate of the Peltier. Cuvette-holding chamber is constantly flushed with a stream of dry nitrogen gas to avoid condensation of water vapour exteriorly over the cuvette at low temperature. Assuming that the quadruplexes undergo two state transitions (folded and unfolded) upon heat-induced denaturation, the molar ellipticity values are normalised with those of 20°C (minimal) and 95°C (maximal) (i.e., a scale of 0-100%) to compare the melting curves(5). The melting profiles are analyzed by plotting the fraction of folded and temperatures in ordinate and abscissa respectively (**Eq. (4)**). Melting temperature ($T_m$) is calculated by fitting the data points of the sigmoidal curve into a two-state transition model (**Eq. (5)**):

Fraction of folded at any temperature,

$$\alpha = ([F])/([F]+[U]) = ((\theta_t - \theta_U))/((\theta_F - \theta_U)) \dots\dots\dots\dots\dots \textbf{Eq. (4)}$$

[F] and [U] are the concentrations of the folded and unfolded forms of G-quadruplex respectively. α denotes the fraction folded at any temperature, t. $\theta_t$ defines the observed ellipticity at any temperature whereas $\theta_F$ and $\theta_U$ are the ellipticity values of the folded and unfolded forms of quadruplexes respectively. The fitting equation is as follows:

$$y = a/(1 + e^{(-k(x-x_c)}) \dots\dots\dots\dots\dots \textbf{Eq. (5)}$$

Here, x is the temperature; a defines the higher asymptote, k is the coefficient and $x_c$ gives the melting temperature or $T_m$, which is conventionally considered as the midpoint between higher and lower asymptotes and is estimated using the fitting parameters. Curves are fitted with high degree of precision, with percentage of variance accounted for >99%. The adequacy of the sigmoidal curve fitting is evaluated by inspection of the weighted residual plots and reduced Chi-squared values ($\chi^2_R$) (*i.e.*, $\chi^2$ divided by degrees of freedom (df), where df is the number of data points minus the number of parameters being fitted).

**1.2.5. Isothermal titration calorimetry:** Thermodynamic attributes of the interaction profiles between peptides and putative quadruplexes are analyzed by Isothermal titration calorimetry using iTC200 Microcalorimeter at 25°C(6). Oligonucleotide sequences and peptides are freshly prepared and degassed under vacuum for 10 minutes ahead of the titrations to ensure the removal of bubbles, if any. Peptides are diluted to a concentration of 20 μM into 10 mM Potassium phosphate buffer (10 mM $K_2HPO_4$ + 10 mM $KH_2PO_4$) containing 0.1 M KCl (pH 7.0). Syringe is filled with the quadruplex sequences (500 μM) present in the upstream promoter regions of *c-MYC* (Pu27, Pu19, and Myc22), *BCL-2* (Pu30), *VEGF-A* (Pu22), *KRAS* (Pu32) oncogenes, and telomere (Tel26), which are injected at an interval of 150 s into the calorimeter cell containing 20 μM of peptides. Another experiment is conducted having KR12C in the calorimeter cell and Pu27 in the syringe. MCF-7 nuclear extract is used as the solvent in order to mimic the cellular microenvironment and impart macromolecular crowding during binding reaction. A control experiment is performed in parallel by injecting the same concentration of oligonucleotides into the identical buffer without peptides to subtract the heat of dilution from peptide-quadruplex binding experiments before curve-fitting. The number of injections are set at 20 or 30 to achieve the binding saturation. The heat of reaction per injection (μcal/s) is determined by integration of the peak areas using in-built Origin 7.0 software, which provides the best-fit values of the enthalpy of binding (ΔH), the stoichiometry of binding (n), and the dissociation constant ($K_d$). Data points are further simulated with "one – site" and/or "sequential" binding modes. The quality of fitting curve is inspected by the reduced Chi-squared values ($\chi^2_R$). Given the total concentration of peptide, L inside the calorimetric cell is known, after each consecutive injection i:

$$[M]_{T,i} = [M]_0(1 - \frac{v}{V})^i \dots\dots\dots\dots\dots\dots \textbf{Eq. (5)}$$

$$[[L]_{T,i}=[L]_0 \times \{1-(1-\frac{v}{V})^i \ldots\ldots\ldots \textbf{Eq. (6)}$$

where $[M]_0$ is the initial concentration of quadruplex in the syringe and $[L]_0$ is the concentration of Peptides in the cell. V signifies the cell volume and v is the injection volume. (1 − v/V) is the factor that accounts for the change in the concentration of reactants due to dilution upon sequential titrations. Therefore, using the mass action law and the conservation of mass for each species:

$$[M]_T = [M] + [ML] = [M] + [K]_{a,L}\,[M][L] \ldots\ldots\ldots\ldots\ldots\ldots \textbf{Eq. (7)}$$
$$[L]_T = [L] + [ML] = [L] + [K]_{a,L}\,[M][L] \ldots\ldots\ldots\ldots\ldots\ldots\ldots \textbf{Eq. (8)}$$

where $[K]_{a,L}$ is the binding constant for Peptides for the quadruplexes. Solving the series of equations yields the concentration of complexes, [ML] in the calorimetric cell after each injection i. The heat released or absorbed due to each injection, $q_i$, is the heat associated with the formation/dissociation of each complex in the injection i:

$$q_i = V[\Delta H_{a,L}\,\{[ML]_i - [ML]_{i-1}\left(1-\frac{v}{V}\right)\}] \ldots\ldots\ldots\ldots\ldots\ldots \textbf{Eq. (9)}$$
$$\Delta G = RTlnK_a \ldots\ldots\ldots\ldots \textbf{Eq. (10)}$$
$$\Delta G = \Delta H - T\Delta S \ldots\ldots\ldots\ldots\ldots \textbf{Eq. (11)}$$

Here, $\Delta H_{a,L}$ is the binding or association enthalpy for each ligand. T is temperature at which the experiment is performed. ΔG is the binding free energy and ΔS is the entropic contribution in each binding event.

**1.2.6. Molecular docking**: The 5'-end of Pu27 is flanked by a tandem stretch of four guanines, which does not participate in the quartet formation and imparts dynamicity to the structure. The 3'-end constitutes a GAG triad wherein a guanine (G27) is plugged back to the third quartet displacing another guanine (G13) from a continuous G-tract into a stable diagonal loop. Therefore, both 5' and 3' sites of Pu27 may serve as the plausible docking sites for the peptides (FK13, KR12A, KR12B, KR12C, KR12D, and KR12E). To validate this, we have performed blind docking as well as constrained docking (biasing constraints over both in 5' and 3' ends) between Pu27 and the peptides using Patchdock server. Then, Firedock server is used to refine the docking models and score them according to their energy functions. This increases the number of highly accurate conformations in the initial selection of poses while reducing the number of false positives (e.g., geometrically distant complexes with good scores) and the poses that require computationally expensive electrostatic calculations(7). For solution NMR structure calculation, we have reiterated the molecular docking protocol between Myc22 and KR12C; because 9-Guaninyl-N1(H) resonances in Myc22 are highly resolved and exhibit improved linewidth in NMR spectra and KR12C is the best peptide candidate to bind to all the conformers of native quadruplex with higher affinity *in vitro* and *in cellulo*. We have further established docking benchmarks by superimposing the docked poses and clustered similar poses reproduced independently by these docking protocols. We have also minimized the docked poses for 500 minimization cycles (first 250 cycles using steepest descent minimization and next 250 cycles using conjugate gradient minimization) in AMBER14 and calculated the all atom root mean square deviation (RMSD) values for each pose (**Supplementary Table S3**). The cluster(s) of binding poses having the RMSD of 1.0–2.0 Å are considered as the near-native conformations and are taken up for the Molecular Dynamics (MD) simulation for the validation and evaluation of the binding pose(s) and molecular interactions.

**1.2.7. Molecular Dynamics Simulation**: All atom molecular dynamics simulations are conducted in explicit solvent using the simulation program AMBER14. Two potassium (K+) ions are placed into the central region of the quartet channels in Pu27 to neutralize the electrostatic repulsion of inwardly pointing guanine oxygens using the xleap module of AMBER14. Ions are parameterized using a General Amber Force Field (GAFF)(8). In contrast, Pu27 quadruplex and the intermolecular complexes are parameterized using the ff14SB force field(9). Potassium ions were incorporated to neutralize the charges, and the systems are submerged within TIP3PBOX water model(10) with box boundaries 10 Å away from the outermost complex atoms along x-, y- and z-directions. This gives a more intuitively realistic picture of intermolecular interactions that retains specific solvent interactions as occurs in the cellular systems. The solvated complexes undergo two-step minimization in vacuum to 0.001 kcal/mol/Å of RMS gradient of potential energy to eliminate the steric clashes, if any. After minimization, we have carried out MD simulation at a time step of 1 fs and gradually increased the temperature of the system to 300 K over 50 ps simulation under NVT conditions. Next, simulations are carried out for another 50 ps under NPT conditions at 1 atm pressure. Then, we have simulated the equilibrated complexes for the production dynamics for 100 ns under NVT conditions. Temperature and pressure are regulated using a Langevin thermostat-barostat, and the SHAKE algorithm is applied for bond length corrections.

Also, Particle Mesh Ewald method is applied for long-range electrostatic interactions with a grid space of 0.1 nm and the non-bonded cut-off is set at 12.0 Å. All the calculations are performed for 2 fs time window and coordinates are recorded at an interval of 10 ps. MD trajectories are analysed using the cpptraj module of AMBER14(11). An ensemble of structures from last 10 ns of MD simulations for complexes are used for the evaluation and analyses of the binding contacts. The snapshots are extracted at an interval of 50 ps resulting in 10 conformations of the complexes for each 100 ns trajectory. The interacting amino acids between the peptides and Pu27 are identified using a distance cut-off of 4.0 Å. Only those contacting residue pairs, which are found in more than 80% of the complexes in last 10 ns of the MD trajectories are considered as the residue pairs having stable interactions. VMD(12) and PyMOL softwares are used for the evaluation and visualization of the simulation trajectories.

1.2.8. **NMR spectroscopy.** Solution NMR structure of the wild-type quadruplex (Pu27) yields a broad envelope of overlapping 9-Guaninyl-N1(H) resonances in the imino proton region ($\delta$10-12 ppm) of 1D $^1$H NMR spectra due to structural ambiguity and shuffling of multiple conformers *in vitro*. Therefore, to map out the binding interface of the peptide-DNA complex at the atomic level, we have used one of the stable conformers of Pu27 quadruplex, Myc22 because, (i) unlike Pu27, the conformer is enriched in a particular topology offering better resolution and improved linewidth of the 9-Guaninyl-N1(H) resonances in the imino proton region; and (ii) the conformer exhibits high affinity interaction with KR12C both *in vitro* and *in cellulo*.

For NMR spectroscopy, Myc22 is diluted to 500 µM in the identical annealing buffer containing 90% water and 10% $D_2O$ and KR12C is slowly titrated upto 5:1 molar ratio. Both one- and two-dimentional NMR experiments are performed in Bruker AVANCE III 700 MHz NMR spectrometer, equipped with a 5 mm SMART probe. All the experiments are carried out in 5 mm NMR tubes having an active sample volume of 600 µL. The spectra are referenced to an internal standard, TSP (3-(trimethylsilyl)-2, 2′, 3, 3′-tetradeuteropropionic acid) at 0.0 ppm. The exchangeable and non-exchangeable protons of Myc22 are observed in the one-dimensional proton spectra under free and KR12C-bound conditions using Bruker Pulse program '**zgesgp**' with a spectral width (sw) of 20 ppm, number of scans (ns) of 512, and calibrated pulse length (p1) of 12.48 µs. 2D $^1$H-$^1$H NOESY spectra of free and peptide-DNA complex is collected using Bruker's '**noesyesgpph**' pulse program at 15°C with a mixing time of 300 ms. Data is processed using Lorentzian-to-Gaussian filtering functions applied in both dimensions, and zero filling to 2048 ($t_1$) and 1024 ($t_2$) data points. Time domain data sets consist of 2048 by 256 complex data points in $t_2$ and $t_1$ dimensions respectively. Structure calculations of the free Myc22 are performed as recommended in the previously published paper (13). 2D spectra are further processed and analysed in Topspin v3.1 and SPARKY softwares for structure calculations.

## 2. Tables

**Table S1**: Putative G-quadruplex forming oligonucleotide sequences located in the promoters of *c-MYC, BCL-2, VEGF-A,* and *KRAS* oncogenes and telomere, used in biophysical studies

| | | **Oligonucleonucleotide sequences (5′– 3′)** |
|---|---|---|
| *c-MYC* | Pu27 (wild-type) | 5′- T GGGG A GGG T GGGG A GGG T GGGG AA GG -3′ |
| | Pu19 | 5′ – T GGGG A GGG T GGGG A GGG T – 3′ |
| | Myc22 | 5′ – GGA GGG T GGGG A GGG T GGGG AA – 3′ |
| *BCL-2* | Pu30 | 5' – AG GGG C GGG CGCGGGAGGAAGG GGG C GGG A – 3' |
| *VEGF-A* | Pu22 | 5' – CGGGGCGGGCCGGGGGCGGGGT – 3' |
| *KRAS* | Pu32 | 5' – AGGGCGGTGTGGGAAGAGGGAAGAGGGGGAGG – 3' |
| Telomere | Tel26 | 5' – AAA GGG TTA GGG TTA GGG TTA GGG AA – 3' |

**Table S2**: Primer sequences used in Real time Polymerase chain reactions along with their corresponding annealing temperature.

| | Oligonucleotide sequences (5′- 3′) | Annealing temperature ($^0$C) |
|---|---|---|
| *c-MYC* | Forward: AATGAAAAGGCCCCCAAGGTAGTTATCC (28 bp)<br>Reverse: GTCGTTTCCGCAACAAGTCCTCTTC (25 bp) | 55 |
| *BCL-2* | Forward: CTGCACCTGACGCCCTTCACC (21 bp)<br>Reverse: CACATGACCCCACCGAACTCAAAGA (25 bp) | 61 |
| *VEGF-A* | Forward: ATCTGCATGGTGATGTTGGA (20 bp)<br>Reverse: GGGCAGAATCATCACGAAGT (20 bp) | 56 |
| *KRAS* | Forward: GCCTGCTGAAAATGACTGAATATA (24 bp)<br>Reverse: TTAGCTGTATCGTCAAGGCACTC (23 bp) | 54 |
| *GAPDH* | Forward: GATGCTGGCGCTGAGTACGTCGTG (24 bp)<br>Reverse: AGTGATGGCATGGACTGTGGTCATGAG (24 bp) | 62.5 |
| *P53* | Forward: GCCCAACAACACCAGCTCCT (20 bp)<br>Reverse: CCTGGGCATCCTTGAGTTCC (20 bp) | 55 |
| *E2F-1* | Forward: GTGTAGGACGGTGAGAGCAC (20 bp)<br>Reverse: TCAAGGGTAGAGGGAGTTGG (20 bp) | 54.5 |

**Table S3:** Docking clusters of the binding poses of Pu27 – Peptides having a total number of nine conformations. 5′ and 3′ sites serve as plausible docking sites. At 5′, Cluster 1 represents three similar number of binding poses while the occurrence of poses in cluster 2 and 3 are lower. Binding poses in cluster 1 show the lowest range all atom RMSD values as compared to the starting conformation. In contrast, the 3′ site displays two clusters having one pose in each cluster with very high RMSD values[2].

| | | KR12A – Pu27 | KR12B – Pu27 | KR12C – Pu27 | KR12D – Pu27 | KR12E – Pu27 | FK13 – Pu27 |
|---|---|---|---|---|---|---|---|
| 5’ | Cluster 1 | 3.1 | 2.21 | 0.76 | 4.2 | 3.3 | 4.1 |
| | | 4.56 | 1.98 | 0.92 | 3.29 | 3.76 | 5.12 |
| | | 4.01 | 2.32 | 0.69 | 7.1 | 4.11 | 2.12 |
| | Cluster 2 | 4.11 | 2.51 | 1.61 | 7.12 | 2.97 | 4.32 |
| | | 4.92 | 2.98 | 0.98 | 6.11 | 3.10 | 4.23 |
| | Cluster 3 | 6.2 | 2.09 | 0.68 | 3.12 | 8.98 | 3.87 |
| | | 6.81 | 4.21 | 2.01 | 19.29 | 7.92 | 12.11 |
| 3’ | Cluster 1 | 11.9 | 10.09 | 12.19 | 12.29 | 11.24 | 13.22 |
| | Cluster 2 | 9.57 | 9.89 | 11.11 | 12.33 | 12.78 | 11.29 |

**Table S4:** Primer sequences used in Chromatin immunoprecipitation (ChIP) experiment:

| | Oligonucleonucleotide sequences (5′– 3′) | Annealing temperature ($^0$C) | Amplicon size (bp) |
|---|---|---|---|
| *c-MYC* – NHE $III_1$ | -453F: GCAGCAGAGAAAGGGAGAGG<br>-215R: GCGGAGATTAGCGAGAGAGG | 56 | 238 |

[2] RMSD values are calculated for all atoms in the minimized Pu27 – peptide complex considering the starting conformation as the reference frame.

**Table S5(a)**: Thermodynamic attributes of interaction between peptides (FK13, KR12A, KR12B, KR12C, KR12D, and KR12E) and multiple conformers of *c-MYC* quadruplex (Myc22 and Pu19) calculated from Isothermal titration calorimetry. Binding enthalpy (ΔH), entropy (ΔS), binding energy (ΔG), dissociation constants ($K_d$), and binding stoichiometry (*n*) are mentioned for each set of binding events[3].

| Complex | ΔG (Kcal.mol$^{-1}$) | ΔH (cal/mol) | ΔS (cal/mol/deg) | *n* | $K_d$ (μM) | $\Delta T_m$ ($^0$C) |
|---|---|---|---|---|---|---|
| **FK13-Myc22** | -110.7 | $-1.159 \times 10^5$ | -371 | 1.2 | 108.9 | 2 |
| **FK13-Pu19** | -91.8 | $-1.027 \times 10^5$ | -326 | 1 | 101 | 0.61 |
| **KR12A-Myc22** | -26.06 | $-2.857 \times 10^4$ | -77.6 | 6.06 | 104.16 | -17.8 |
| **KR12A-Pu19** | -27.65 | $-2.962 \times 10^4$ | -78.8 | 3.18 | 32.78 | -12.98 |
| **KR12B-Myc22** | -10.425 | $-1.079 \times 10^4$ | -14.6 | 4.03 | 19.4 | 0.97 |
| **KR12B-Pu19** | -52.59 | $-5.687 \times 10^5$ | -171 | 2.21 | 60.97 | 2.32 |
| **KR12C-Myc22** | -12.05 | $-1.26 \times 10^5$ | -21.9 | 3.17 | 30.21 | 10.87 |
| **KR12C-Pu19** | -15.63 | $-1.645 \times 10^4$ | -32.7 | 4.5 | 12.31 | 6.91 |
| **KR12D-Myc22** | -128.32 | $-1.39 \times 10^5$ | -447 | 1 | 59.17 | 0.98 |
| **KR12D-Pu19** | -7.6 | -7693 | -3.61 | 12.5 | 14.22 | 0.33 |
| **KR12E-Myc22** | -91.6 | $-1.06 \times 10^5$ | -336 | 1 | 38.46 | -5.35 |
| **KR12E-Pu19** | $-3.1 \times 10^5$ | $-3.486 \times 10^8$ | $-1.17 \times 10^6$ | 0.0163 | 1243.78 | -10.02 |

**Table S5(b)**: Thermodynamic attributes of interaction between peptides (FK13, KR12A, KR12B, KR12C, KR12D, and KR12E) and Telomere (Tel26) calculated from Isothermal titration calorimetry. Binding enthalpy (ΔH), entropy (ΔS), binding energy (ΔG), dissociation constants ($K_d$), and binding stoichiometry (*n*) are mentioned for each set of binding events[4].

| Complex | ΔG (Kcal.mol$^{-1}$) | ΔH (cal/mol) | ΔS (cal/mol/deg) | *n* | $K_d$ (μM) |
|---|---|---|---|---|---|
| **FK13-Tel26** | -5.72 | $-5.43 \times 10^4$ | -163 | 1.2 | 67.89 |
| **KR12A- Tel26** | -6.08 | $-2.915 \times 10^4$ | -77.4 | 1.3 | 37.4 |
| **KR12B- Tel26** | -6.31 | $-4.982 \times 10^4$ | -146 | 1.03 | 25.2 |
| **KR12C- Tel26** | -6.38 | $-7.16 \times 10^4$ | -219 | 1.17 | 22.5 |
| **KR12D- Tel26** | -6.79 | $-4.463 \times 10^4$ | -127 | 1.5 | 11.31 |
| **KR12E- Tel26** | - | - | - | - | No binding |

**Table S5(c)**: Thermodynamic attributes of interaction between KR12C and other oncogenic quadruplexes (*BCL-2*, *VEGF-A*, and *KRAS*) calculated from Isothermal titration calorimetry. Binding enthalpy (ΔH), entropy (ΔS), binding energy (ΔG), dissociation constants ($K_d$), and binding stoichiometry (*n*) are mentioned for each set of binding events[5].

| Complex | ΔG (Kcal.mol$^{-1}$) | ΔH (Kcal/mol) | ΔS (cal/mol/deg) | *n* | $K_d$ (μM) |
|---|---|---|---|---|---|
| **KR12C-*BCL-2*** | -6.25 | -1.336 | 16.3 | 5.2 | 26 |
| **KR12C-*VEGF-A*** | - | - | - | - | No binding |
| **KR12C-*KRAS*** | - | - | - | - | No binding |

3 All these thermodynamic parameters are simulated with "one-site" binding mode.

4 All these thermodynamic parameters are simulated with "one-site" binding mode.

5 All these thermodynamic parameters are simulated with "one-site" binding mode.

**Table S6: NMR distance and dihedral constraints**

| NMR distance and dihedral constraints | |
|---|---|
| Distance constraints | |
| Total NOE | 355 |
| Inter residual NOE | 348 |
| Intra residual NOE | 7 |
| Strong (\|*i* – *j*\|≤3) | 72 |
| Medium range (\|*i* – *j*\|≤4) | 256 |
| Long range (\|*i* – *j*\|≥5) | 27 |
| Total dihedral angle restraints | |
| Sugar restraints | 27 |
| Backbone restraints | |
| Gamma | 25 |
| Chi | 25 |
| Epsilon | 25 |
| Beta | 25 |
| Hoogsteen bonding restrains | 24 |
| Structure statistics | |
| Violations (Mean and S.D.) | |
| Deviation from idealized geometry | |
| Bond length | $1.5 \pm 0.0234$ |
| Bond angle | $3.98 \pm 0.0043$ |
| Average pairwise r.m.s.d. (Å) | |
| Backbone | 0.358 |
| Heavy atoms | 0.957 |

**Table S7: Details of the primer sequences for cloning**. GQ-null is the insert (-467 to +36 bases of c-MYC promoter), which lacks the quadruplex forming sequences within Nuclease hypersensitivity element III1 (NHE III1). Pu19C and Myc22Care the mutant vectors. Wild-type primers are used for final extension of the one-step and two-step overlap-PCR experiments while creating the mutant vectors.

| | | Primer sequences for reporter luciferase constructs (5′-3′)[6] | Size (bp) | Annealing temperature ($^0$C) |
|---|---|---|---|---|
| P1 – P2 | GQ-null | -107GQ-null FP: CGAGCC<u>GGTACC</u>TCGAGAAGGGCAGGGCTTCTCAGAGGCTTGGCGGG[7] | 47 | 67 |
| | | GQ-null RP: CGCTCC<u>AAGCTT</u>GGCCGCCCGCTCGCTCCCTCTGCC[8] | 36 | 69.2 |
| | Pu22C | 1st round of PCR | 44 | 69.2 |
| | | -335 FP[9]: CATTCCCCACCCTCCCCACCCTCCTAAGCGCCCCTCCCGGGTTC | | |
| | | Pu24 RP[10]: GAACCCGGGAGGGGCGCTTAGGAGGGTGGGGAGGGTGGGGAATG | | |
| | | 2nd round of PCR | 37 | 68.7 |
| | | -319m FP: GGCTGAGTCTCCTCCCCATTCCCCACCCTCCCCACCC | | |
| | | Pu25 RP: GGGTGGGGAGGGTGGGGAATGGGGAGGAGACTCAGCC | | |

6 All the primers for cloning are obtained from Biobharati India Pvt. Ltd. All the mutant constructs are created over Pu27 wild-type template and cloned into the KpnI and HindIII sites of pGL4.72 [hRlucCP] luciferase vector.

7 GGTACC: KpnI restriction site

8 AAGCTT: HindIII restriction site

9 FP (Forward primer)

10 and RP (Reverse primer)

| | | | | |
|---|---|---|---|---|
| | Pu19C | -317 FP: GGCTGAGTCTCCTCCCCAACCCTCCCCACCCTCCCCATAAG | 41 | 66 |
| | | Pu19 RP: CTTATGGGGAGGGTGGGGAGGGTTGGGGAGGAGACTCAGCC | | |
| Wild-type | | -467wt FP: CGAGCCGGTACCACTGCTACGGAGGAGCAGCAGAGAAAGGGAGAGGG | 47 | 67.5 |
| | | wt RP: CGCTCCAAGCTTGGCCGCCCGCTCGCTCCCTCTGCCTC | 38 | 68.5 |

## 3. Supplementary Figures

**Figure S1**: **Design of therapeutic peptides**. Site-specific amino acid substitution in designed peptides based on the quadruplex-binding domain of LL37. Amino acids (17 – 29) in LL37 underlined denote the active quadruplex-binding domain. Prediction of the net charge and anti-cancer potential by AntiCP prediction; potential of cell permeability and cellular toxicity of designed peptides using CellPPD and ToxinPred prediction respectively.

**Figure S2**: ***c-MYC* promoter repression by synthetic peptides under the control of biologically relevant quadruplex intermediates (Myc22 and Pu19) within native Pu27 by dual-luciferase assays**. (**A**) Luciferase vector map**.** Diagram of the pGL4.72[*hRlucCP*] vector having the insert containing *c-MYC* promoter sequences (P1 and P2) and upstream Nuclease Hypersensitive Elements (NHE $III_1$) ahead of the *hRluc* coding region. *hRluc*, *Renilla* luciferase gene; *hCL1* and *hPEST*, protein destabilizing sequences; oriC, origin of replication; AmpR, ampicillin resistance gene; SV40 (Simian virus 40 polyadenylation signal cassette), (**B**) *c-MYC* promoter sequences are cloned into KpnI and HindIII restriction sites with or without the biologically relevant quadruplex intermediates (Myc22, and Pu19) into NHE $III_1$. Myc22 lacks G-tract I and VI while G-tracts V and VI are absent in Pu19. (**C**) *c-MYC* promoter repression under the influence of Myc22 and (**D**) Pu19 motifs with an increasing concentration of peptides (FK13, KR12A, KR12B, KR12C, KR12D, and KR12E). Relative promoter activity is determined by normalizing the Rluc/Fluc values to that of the cells transfected with P1-P2 promoter construct (GQ-null), having no quadruplex-forming motif. Error bars represent mean ± SE ($N = 3$). Statistical differences in the luciferase activities between untreated and treated cells used one-way ANOVA followed by Tukey Kramer's Post hoc Test (*P < 0.05, **P < 0.01, ***P < 0.001). Pairwise comparison in the luciferase activities between GQ-null construct and the vectors having Myc22 and Pu19 motifs are performed by two tailed Student's *t* test (#P < 0.05, ##P < 0.01, ###P < 0.001).

**Figure S3: Binding profiles of synthetic peptides (FK13, KR12A, KR12B, KR12C, KR12D, and KR12E) and intermediate quadruplex isomers of Pu27 (Myc22 and Pu19).** (**A**) Isothermal titration calorimetry. Myc22 interaction profile with the peptides. (**B**) Pu19 interaction profile with the peptides. Top panels: enthalpic heat released versus time at 25°C during titrations. Bottom panels: thermogram of the integrated peak intensities plotted against the molar ratio of the complex. Best-fit curves using single-site binding model.

**Figure S4: Binding profiles of synthetic peptides (FK13, KR12A, KR12B, KR12C, KR12D, and KR12E) and telomeric G-quadruplex.** Top panels: heat rate versus time at 25°C during titrations. Bottom panels: thermogram of the integrated peak intensities plotted against the molar ratio of the complex. Best-fit curves using single-site binding model.

**Figure S5: Binding profiles of synthetic peptide KR12C and *BCL-2, KRAS* and *VEGF-A* G-quadruplex.** Top panels: heat rate versus time at 25°C during titrations. Bottom panels: thermogram of the integrated peak intensities plotted against the molar ratio of the complex. Best-fit curves using single-site binding model

**Figure S6(A): Circular Dichroism titration profiles of synthetic peptides ((A) FK13, (B) KR12A, (C) KR12B, (D) KR12C, (E) KR12D, and (F) KR12E) with Pu27.** The change in ellipticity was plotted against wavelength with increasing concentrations (in μM) of peptides. All experiments were carried out using 10 mM potassium phosphate buffer containing 100 mM potassium chloride at pH 7.0

**Figure S6(B): Circular Dichroism titration profiles of synthetic peptides (FK13, KR12A, KR12B, KR12C, KR12D, and KR12E) and intermediate quadruplex isomers of Pu27 (Myc22 and Pu19).** The change in ellipticity was plotted against wavelength. All experiments were carried out using 10 mM potassium phosphate buffer containing 100 mM potassium chloride at pH 7.0

**Figure S7: *In silico* modelling and simulation of Pu27-KR12C complex.** (**A**) An ensemble of 10 structures of Pu27-KR12C complex over last 10 ns of a 100 ns simulation in explicit solvent. (**B**) The root mean square values (RMSD) values (in Å) of the binding interface in Pu27 and KR12C are plotted versus the number of frames across 100 ns under bound condition. Dotted line indicates the average RMSD value (right of the plot). (**C**) Electrostatic interaction between R12 and sugar-phosphate backbone A6. (**D**) R2 side-chain interaction with G14 and G16 bases. (**E**) $CH_3$-π interaction by L11 and I7 at A6 and A15. Induced Hoogsteen pairing between A6 and A15. The distance (in Å) between the interacting atoms are shown at 25 ns interval over 100 ns of the production run. (**F**) Schematic representation of the selective interaction between Pu27 and KR12C for quadruplex selectivity.

**Figure S8: *In silico* modelling and simulation of Pu27-FK13, Pu27-KR12A, Pu27-KR12B, Pu27-KR12D, and Pu27-KR12E complexes.** (**A**) An ensemble of 10 structures of and (**A**) Pu27-FK13 , (**B**) Pu27-KR12A, (**C**) Pu27-KR12B, (**D**) Pu27-KR12D, and (**E**) Pu27-KR12E complexes over last 10 ns of a 100 ns simulation in explicit solvent. Non-covalent interresidual interactions are shown in right.

**Figure S9: $^1$H-$^1$H NOESY Spectra of Myc22 complexed with KR12C.** Intraresidual H1/H8 NOE crosspeaks showing tetrad plane alignment in Myc22. Interresidual NOE crosspeaks between H1 of guanine base and (NH) of amino acids.

**Figure S10: $^1$H-$^1$H NOESY Spectra of Myc22 complexed with KR12C.** Intraresidual H1'/H2', H1'/H2" NOE (Nuclear Overhauser effect) crosspeaks of deoxyribonucleotide sugars in Myc22. Interresidual crosspeaks between H1' of the sugar and Hb of the amino acids (Marked in Red).

**Figure S11: Expression profile of *BCL2, VEGF-A* and *KRAS* transcripts upon the treatment of FK13, KR12A and KR12B.**

**Figure S12: KR12C driven signalling pathway.** (**A**) Western blot analysis. BCL-2 and c-MYC expression at 5 and 10 μM of KR12C treatment, combined treatment of KR12C and recombinant VEGF (rVEGF), and rVEGF treatment. β- actin used as the housekeeping protein. (**B**) Estimation of c-MYC and (**C**) BCL-2 at protein levels by densitometric analyses. Error bars represent mean ± SE ($N$ = 3). Statistical differences in the protein expression compared to the control cell used one-way ANOVA followed by Tukey Kramer's Post hoc Test (*$P < 0.05$, **$P < 0.01$, ***$P < 0.001$).

## 4. Sequence information of cloning details

### (A) Cloning of *c-MYC* promoter (P1 and P2) having Myc22 G-quadruplex element in NHE III$_1$ into pGL4.72[*hRlucCP*] (KpnI - HindIII)

```
>1st_BASE_2261086_PL3b_RV_Primer_3
TGGCAAGGCAGAGCAGACTTTCTCTGGCCTAACTGGCCGGTACCACTGCTACGGAGGAGCAGCAGAGAAAGGGAGAGGGTTTGAGAGGGAGCA
AAAGAAAATGGTAGGCGCGCGTAGTTAATTCATGCGGCTCTCTTACTCTGTTTACATCCTAGAGCTAGAGTGCTCGGCTGCCCGGCTGAGTCT
CCTCCCCATTCCCCACCCTCCCCACCCTCCTAAGCGCCCCTCCCGGGTTCCCAAAGCAGAGGGCGTGGGGGAAAAGAAAAAAGATCCTCTCTC
GCTAATCTCCGCCCACCGGCCCTTTATAATGCGAGGGTCTGGACGGCTGAGGACCCCCGAGCTGTGCTGCTCGCGGCCGCCACCGCCGGGCCC
CGGCCGTCCCTGGCTCCCCTCCTGCCTCGAGAAGGGCAGGGCTTCTCAGAGGCTTGGCGGGAAAAAGAACGGAGGGAGGGATCGCGCTGAGTA
TAAAAGCCGGTTTTCGGGGCTTTATCTAACTCGCTGTAGTAATTCCAGCGAGAGGCAGAGGGAGCGAGCGGGCGGCCAAGCTTGGCAATCCGG
TACTGTTGGTAAAGCCACCATGGCTTCCAAGGTGTACGACCCCGAGCAACGCAAACGCATGATCACTGGGCCTCAGTGGTGGGCTCGCTGCAA
GCAAATGAACGTGCTGGACTCCTTCATCAACTACTATGATTCCGAGAAGCACGCCGAGAACGCCGTGATTTTTCTGCATGGTAACGCTGCCTC
CAGCTACCTGTGGAGGCACGTCGTGCCTCACATCGAGCCCGTGGCTAGATGCATCATCCCTGATCTGATCGGAATGGGTAAGTCCGGCAAGAG
CGGGAATGGCTCATATCGCCTCCTGGATCACTACAAGTACCTCACCGCTTGGTTCGAGCTGCTGAACCTTCCAAAGAAAATCATCTTTGTGGG
CCACGACTGGGGGGCTTGTCTGGCCTTTCACTACTCCTACGAGCACCAAGACAAGATCAAGGCCATCGTCCATGCTGAAAATGTCCTGGACGT
GATCCAATCCTGGGACGAGTGGCCTGACATCGAGGAGGATATCGCCCTGATCAAGAGCGAAGAGGGCGAGAAAATGGTGCTTGAAAATAACTT
CTTCGTCAAGACCATGCTCCCAAGCAAGATCATGCGGAAACTGGAACCTGAAGAATTCCCTGCCTACCTGGAGCCTTTCAGGGAAAGGGCGAG
GTTAAACGGCCTACCCTCTCCGGGCCTCCCGGAATCCCTTTCCTTAAGGGAAGGCAAGCCGAATCCCTCCAAATTGTCCCAAATACCAAGCCT
AACTTCGGGCCCGGACAATTTGCTTAAAAGTTTCCCGGTTCCACCTGGGTTTTTTTCCACCGTTTGTGCAGAGGGCTAAAAGTTCCCTAACCC
GAGTTTTGAAGGGGAAGGGCTCCCCTCCCCCCGGAGAACCTCCCATAAAAGGGGGAATTCCAAAACTTTTGGTGGGCGGTGTTACAAAACAAA
TTCTTTTACAGGGGTTTAATTCAAGCATTCTATGCTCTAAAACAACGTCTCTCTACCGAGGGAGAGGGCGGGCCCCCTCTTTTGCCCAGAAAA
GGAGTGACAAATGTTTCCGCCTCCCGGACAAAATAAAAGACTGCGCGCGCACACGCAAAATATAAAAAGTTAAAGATTAACCAG
```

**GGTACC**: KpnI cut site, **AAGCTT**: HindIII cut site

(Sequence in blue denotes the complimentary strand of NHE III$_1$ in *c-MYC* promoter. The part of sequence highlighted in yellow is the Pu22 quadruplex motif after deleting G-tract-I and G-tract-VI)

| Score | Expect | Identities | Gaps | Strand |
|---|---|---|---|---|
| 896 bits(485) | 0.0 | 498/503(99%) | 5/503(0%) | Plus/Plus |

```
Query  1    ACTGCTACGGAGGAGCAGCAGAGAAAGGGAGAGGGTTTGAGAGGGAGCAAAAGAAAATGG  60
            ||||||||||||||||||||||||||||||||||||||||||||||||||||||||||||
Sbjct  1    ACTGCTACGGAGGAGCAGCAGAGAAAGGGAGAGGGTTTGAGAGGGAGCAAAAGAAAATGG  60

Query  61   TAGGCGCGCGTAGTTAATTCATGCGGCTCTCTTACTCTGTTTACATCCTAGAGCTAGAGT  120
            ||||||||||||||||||||||||||||||||||||||||||||||||||||||||||||
Sbjct  61   TAGGCGCGCGTAGTTAATTCATGCGGCTCTCTTACTCTGTTTACATCCTAGAGCTAGAGT  120

Query  121  GCTCGGCTGCCCGGCTGAGTctcctccccaccttccccaccctccccaccctccccataa  180
            ||||||||||||||||||||||||||||||  ||||||||||||||||||||||   |||
Sbjct  121  GCTCGGCTGCCCGGCTGAGTCTCCTCCCCA--TTCCCCACCCTCCCCACCCTCC---TAA  175

Query  181  gcgcccctcccgggttcccaAAGCAGAGGGCGTGGGGGAAAAGAAAAAAGATCCTCTCTC  240
            ||||||||||||||||||||||||||||||||||||||||||||||||||||||||||||
Sbjct  176  GCGCCCCTCCCGGGTTCCCAAAGCAGAGGGCGTGGGGGAAAAGAAAAAAGATCCTCTCTC  235

Query  241  GCTAATCTCCGCCCACCGGCCCTTTATAATGCGAGGGTCTGGACGGCTGAGGACCCCCGA  300
            ||||||||||||||||||||||||||||||||||||||||||||||||||||||||||||
Sbjct  236  GCTAATCTCCGCCCACCGGCCCTTTATAATGCGAGGGTCTGGACGGCTGAGGACCCCCGA  295

Query  301  GCTGTGCTGCTCGCGGCCGCCACCGCCGGGCCCCGGCCGTCCCTGGCTCCCCTCCTGCCT  360
            ||||||||||||||||||||||||||||||||||||||||||||||||||||||||||||
Sbjct  296  GCTGTGCTGCTCGCGGCCGCCACCGCCGGGCCCCGGCCGTCCCTGGCTCCCCTCCTGCCT  355

Query  361  CGAGAAGGGCAGGGCTTCTCAGAGGCTTGGCGGGAAAAAGAACGGAGGGAGGGATCGCGC  420
            ||||||||||||||||||||||||||||||||||||||||||||||||||||||||||||
Sbjct  356  CGAGAAGGGCAGGGCTTCTCAGAGGCTTGGCGGGAAAAAGAACGGAGGGAGGGATCGCGC  415

Query  421  TGAGTATAAAAGCCGGTTTTCGGGGCTTTATCTAACTCGCTGTAGTAATTCCAGCGAGAG  480
            ||||||||||||||||||||||||||||||||||||||||||||||||||||||||||||
Sbjct  416  TGAGTATAAAAGCCGGTTTTCGGGGCTTTATCTAACTCGCTGTAGTAATTCCAGCGAGAG  475

Query  481  GCAGAGGGAGCGAGCGGGCGGCC  503
            |||||||||||||||||||||||
Sbjct  476  GCAGAGGGAGCGAGCGGGCGGCC  498
```

**(B) Cloning of *c-MYC* promoter (P1 and P2) having Pu19 G-quadruplex element in NHE III$_1$ into pGL4.72[*hRlucCP*] (KpnI - HindIII)**

```
>1st_BASE_2261086_PL3b_RV_Primer_3
TGGCAAGGCAGAGCAGACTTTCTCTGGCCTAACTGGCCGGTACCACTGCTACGGAGGAGCAGCAGAGAAAGGGAGAGGGTTTGAGAGGGAGCA
AAAGAAAATGGTAGGCGCGCGTAGTTAATTCATGCGGCTCTCTTACTCTGTTTACATCCTAGAGCTAGAGTGCTCGGCTGCCCGGCTGAGTCT
CCTCCCCAACCCTCCCCACCCTCCCCATAAGCGCCCCTCCCGGGTTCCCAAAGCAGAGGGCGTGGGGGAAAAGAAAAAAGATCCTCTCTCGCT
AATCTCCGCCCACCGGCCCTTTATAATGCGAGGGTCTGGACGGCTGAGGACCCCCGAGCTGTGCTGCTCGCGGCCGCCACCGCCGGGCCCCGG
CCGTCCCTGGCTCCCCTCCTGCCTCGAGAAGGGCAGGGCTTCTCAGAGGCTTGGCGGGAAAAAGAACGGAGGGAGGGATCGCGCTGAGTATAA
AAGCCGGTTTTCGGGGCTTTATCTAACTCGCTGTAGTAATTCCAGCGAGAGGCAGAGGGAGCGAGCGGGCGGCCAAGCTTGGCAATCCGGTAC
TGTTGGTAAAGCCACCATGGCTTCCAAGGTGTACGACCCCGAGCAACGCAAACGCATGATCACTGGGCCTCAGTGGTGGGCTCGCTGCAAGCA
AATGAACGTGCTGGACTCCTTCATCAACTACTATGATTCCGAGAAGCACGCCGAGAACGCCGTGATTTTTCTGCATGGTAACGCTGCCTCCAG
CTACCTGTGGAGGCACGTCGTGCCTCACATCGAGCCCGTGGCTAGATGCATCATCCCTGATCTGATCGGAATGGGTAAGTCCGGCAAGAGCGG
GAATGGCTCATATCGCCTCCTGGATCACTACAAGTACCTCACCGCTTGGTTCGAGCTGCTGAACCTTCCAAAGAAAATCATCTTTGTGGGCCA
CGACTGGGGGGCTTGTCTGGCCTTTCACTACTCCTACGAGCACCAAGACAAGATCAAGGCCATCGTCCATGCTGAAAATGTCCTGGACGTGAT
CCAATCCTGGGACGAGTGGCCTGACATCGAGGAGGATATCGCCCTGATCAAGAGCGAAGAGGGCGAGAAAATGGTGCTTGAAAATAACTTCTT
CGTCAAGACCATGCTCCCAAGCAAGATCATGCGGAAACTGGAACCTGAAGAATTCCCTGCCTACCTGGAGCCTTTCAGGGAAAGGGCGAGGTT
AAACGGCCTACCCTCTCCGGGCCTCCCGGAATCCCTTTCCTTAAGGGAAGGCAAGCCGAATCCCTCCAAATTGTCCCAAATACCAAGCCTAAC
TTCGGGCCCGGACAATTTGCTTAAAAGTTTCCCGGTTCCACCTGGGTTTTTTTCCACCGTTTGTGCAGAGGGCTAAAAGTTCCCTAACCCGAG
TTTTGAAGGGGAAGGGCTCCCCTCCCCCCGGAGAACCTCCCATAAAAGGGGGAATTCCAAAACTTTTGGTGGGCGGTGTTACAAAACAAATTC
TTTTACAGGGGTTTAATTCAAGCATTCTATGCTCTAAAACAACGTCTCTCTACCGAGGGAGAGGGCGGGCCCCCTCTTTTGCCCAGAAAAGGA
GTGACAAATGTTTCCGCCTCCCGGACAAAATAAAAGACTGCGCGCGCACACGCAAAATATAAAAAGTTAAAGATTAACCAG
```

**GGTACC**: KpnI cut site, **AAGCTT**: HindIII cut site

(Sequence in blue denotes the complimentary strand of NHE III$_1$ in *c-MYC* promoter. The part of sequence highlighted in yellow is the Pu19 quadruplex motif after deleting G-tract-V and G-tract-VI)

| Score | Expect | Identities | Gaps | Strand |
|---|---|---|---|---|
| 878 bits(475) | 0.0 | 495/503(98%) | 8/503(1%) | Plus/Plus |

```
Query  1    ACTGCTACGGAGGAGCAGCAGAGAAAGGGAGAGGGTTTGAGAGGGAGCAAAAGAAAATGG  60
            ||||||||||||||||||||||||||||||||||||||||||||||||||||||||||||
Sbjct  1    ACTGCTACGGAGGAGCAGCAGAGAAAGGGAGAGGGTTTGAGAGGGAGCAAAAGAAAATGG  60

Query  61   TAGGCGCGCGTAGTTAATTCATGCGGCTCTCTTACTCTGTTTACATCCTAGAGCTAGAGT  120
            ||||||||||||||||||||||||||||||||||||||||||||||||||||||||||||
Sbjct  61   TAGGCGCGCGTAGTTAATTCATGCGGCTCTCTTACTCTGTTTACATCCTAGAGCTAGAGT  120

Query  121  GCTCGGCTGCCCGGCTGAGTctcctccccaccttccccaccctccccaccctccccataa  180
            ||||||||||||||||||||||||||||||        ||||||||||||||||||||||
Sbjct  121  GCTCGGCTGCCCGGCTGAGTCTCCTCCCCA--------ACCCTCCCCACCCTCCCCATAA  172

Query  181  gcgcccctcccgggttcccaAAGCAGAGGGCGTGGGGGAAAAGAAAAAAGATCCTCTCTC  240
            ||||||||||||||||||||||||||||||||||||||||||||||||||||||||||||
Sbjct  173  GCGCCCCTCCCGGGTTCCCAAAGCAGAGGGCGTGGGGGAAAAGAAAAAAGATCCTCTCTC  232

Query  241  GCTAATCTCCGCCCACCGGCCCTTTATAATGCGAGGGTCTGGACGGCTGAGGACCCCCGA  300
            ||||||||||||||||||||||||||||||||||||||||||||||||||||||||||||
Sbjct  233  GCTAATCTCCGCCCACCGGCCCTTTATAATGCGAGGGTCTGGACGGCTGAGGACCCCCGA  292

Query  301  GCTGTGCTGCTCGCGGCCGCCACCGCCGGGCCCCGGCCGTCCCTGGCTCCCCTCCTGCCT  360
            ||||||||||||||||||||||||||||||||||||||||||||||||||||||||||||
Sbjct  293  GCTGTGCTGCTCGCGGCCGCCACCGCCGGGCCCCGGCCGTCCCTGGCTCCCCTCCTGCCT  352

Query  361  CGAGAAGGGCAGGGCTTCTCAGAGGCTTGGCGGGAAAAAGAACGGAGGGAGGGATCGCGC  420
            ||||||||||||||||||||||||||||||||||||||||||||||||||||||||||||
Sbjct  353  CGAGAAGGGCAGGGCTTCTCAGAGGCTTGGCGGGAAAAAGAACGGAGGGAGGGATCGCGC  412

Query  421  TGAGTATAAAAGCCGGTTTTCGGGGCTTTATCTAACTCGCTGTAGTAATTCCAGCGAGAG  480
            ||||||||||||||||||||||||||||||||||||||||||||||||||||||||||||
Sbjct  413  TGAGTATAAAAGCCGGTTTTCGGGGCTTTATCTAACTCGCTGTAGTAATTCCAGCGAGAG  472

Query  481  GCAGAGGGAGCGAGCGGGCGGCC  503
            |||||||||||||||||||||||
Sbjct  473  GCAGAGGGAGCGAGCGGGCGGCC  495
```

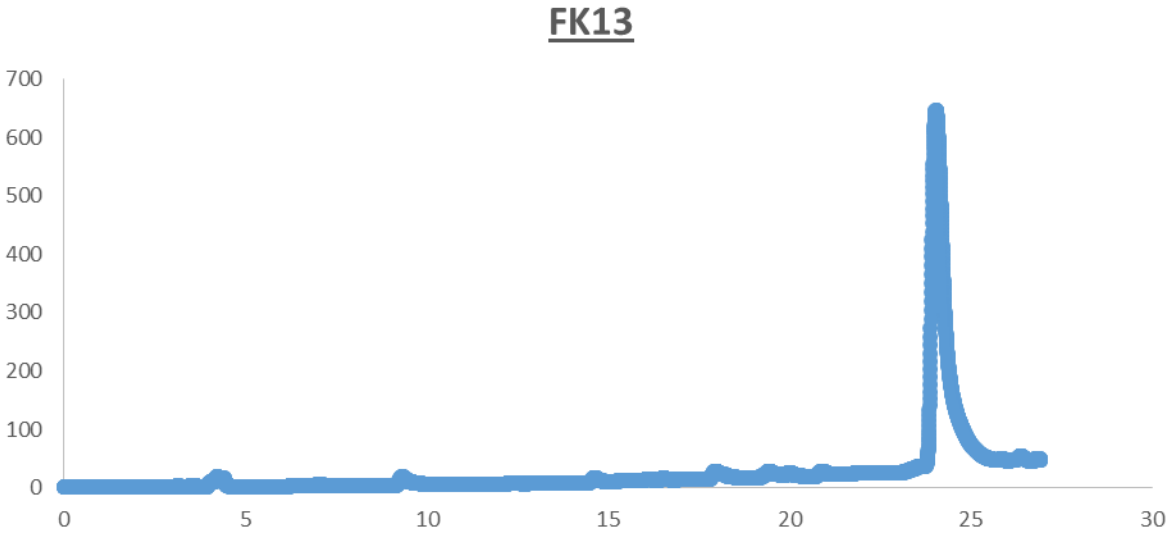


| Peak No. | Reten. time | Area [mV.s] | Height [mV] | Area [%] | Height [%] |
|---|---|---|---|---|---|
| | | | | | |
| 1 | 24.04 | 16596.59 | 611.926 | 95.542 | 95.429 |
| 2 | 28.267 | 774.3399 | 29.308 | 4.458 | 4.571 |
| | Total | 17370.93 | 641.234 | 100 | 100 |

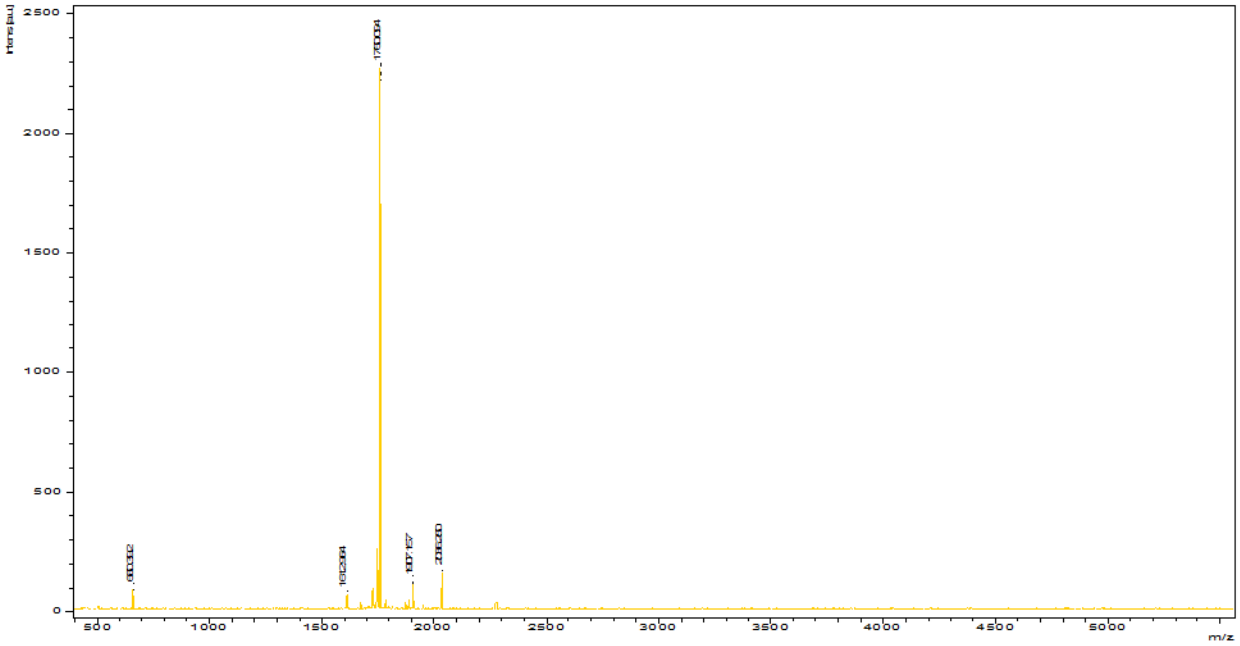


*Figure 5 (A). Chromatogram and Mass Spectrum of FK13*

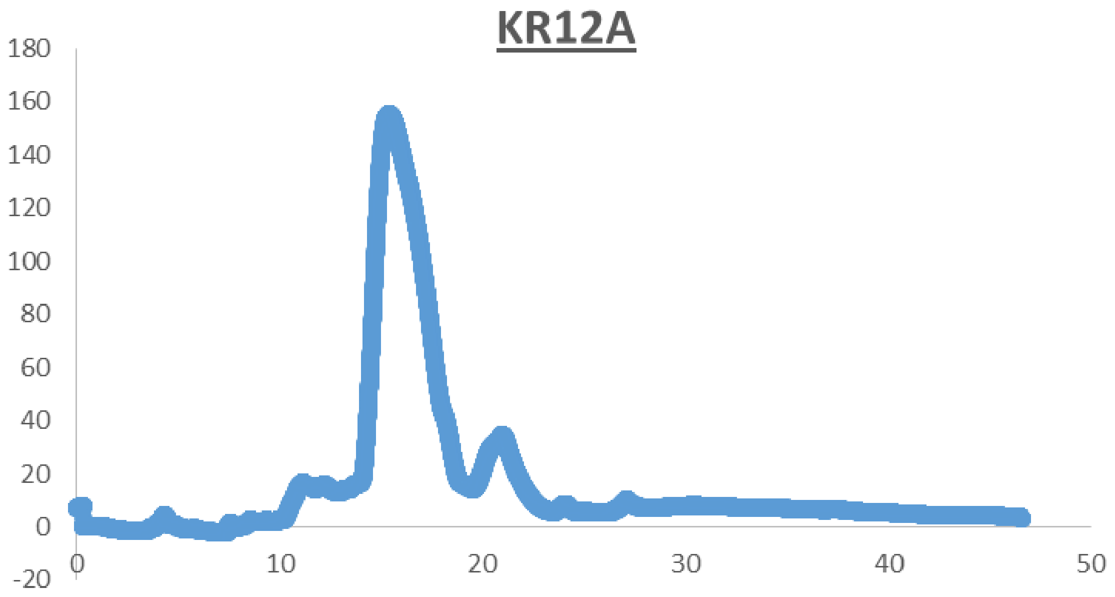


| Peak No. | Reten. time | Area [mV.s] | Height [mV] | Area [%] | Height [%] |
|---|---|---|---|---|---|
| | | | | | |
| 1 | 15.397 | 28356.33 | 155.773 | 86.239 | 81.918 |
| 2 | 20.983 | 4524.943 | 34.384 | 13.761 | 18.082 |
| | Total | 32881.27 | 190.157 | 100 | 100 |

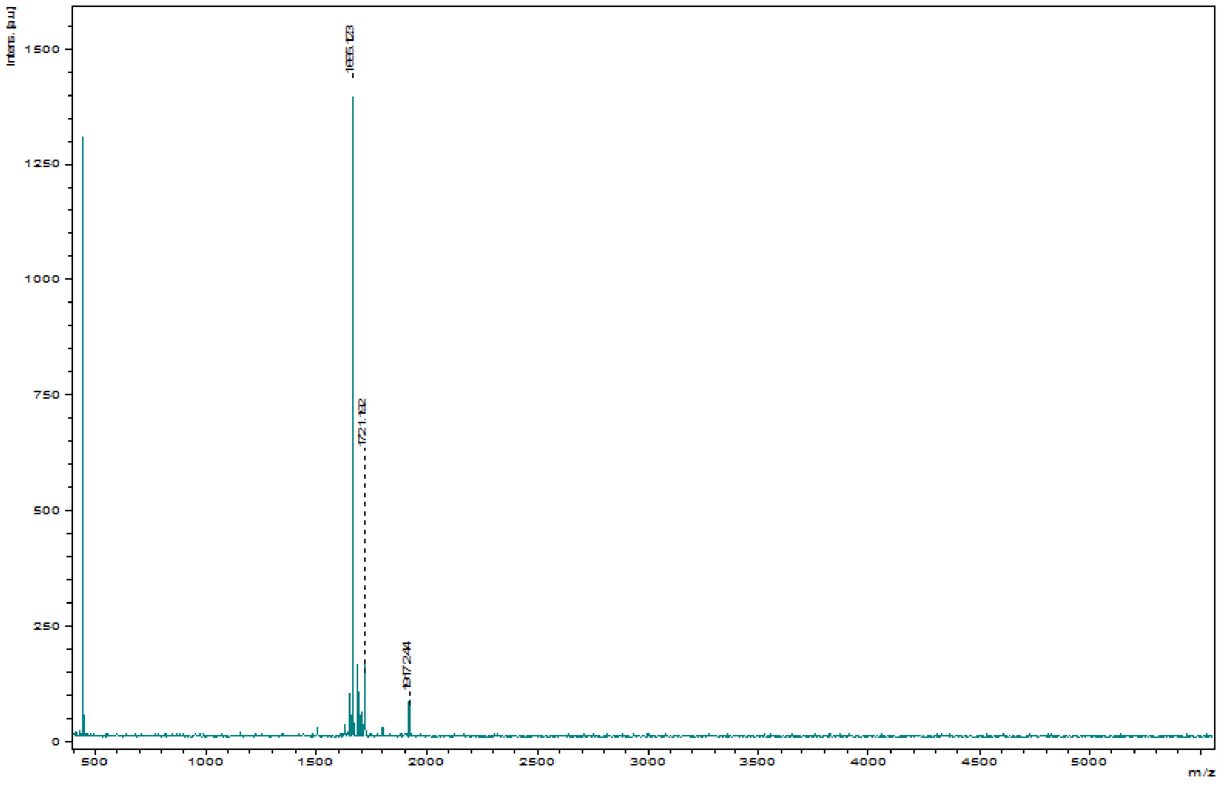


*Figure 5(B). Chromatogram and Mass Spectrum of KR12A*

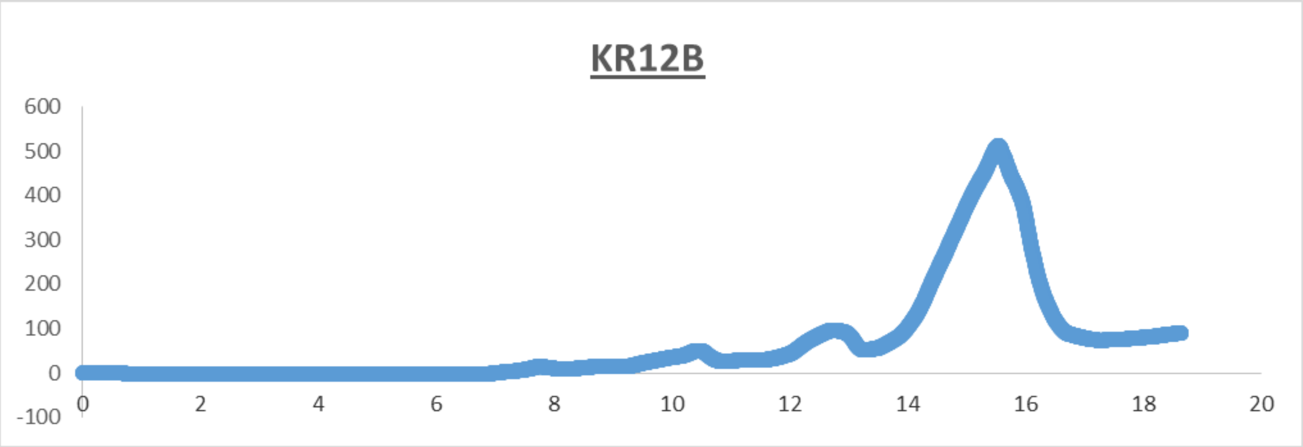


| Peak No. | Reten. time | Area [mV.s] | Height [mV] | Area [%] | Height [%] |
|---|---|---|---|---|---|
| | | | | | |
| 1 | 7.2 | 29.0293 | 3.632 | 0.065 | 0.659 |
| 2 | 7.78 | 266.0457 | 9.454 | 0.598 | 1.714 |
| 3 | 8.82 | 94.2845 | 2.255 | 0.212 | 0.409 |
| 4 | 10.443 | 1266.728 | 28.407 | 2.846 | 5.151 |
| 5 | 12.74 | 2974.266 | 56.913 | 6.683 | 10.32 |
| 6 | 15.54 | 39876.26 | 450.835 | 89.596 | 81.748 |
| | Total | 44506.62 | 551.496 | 100 | 100 |

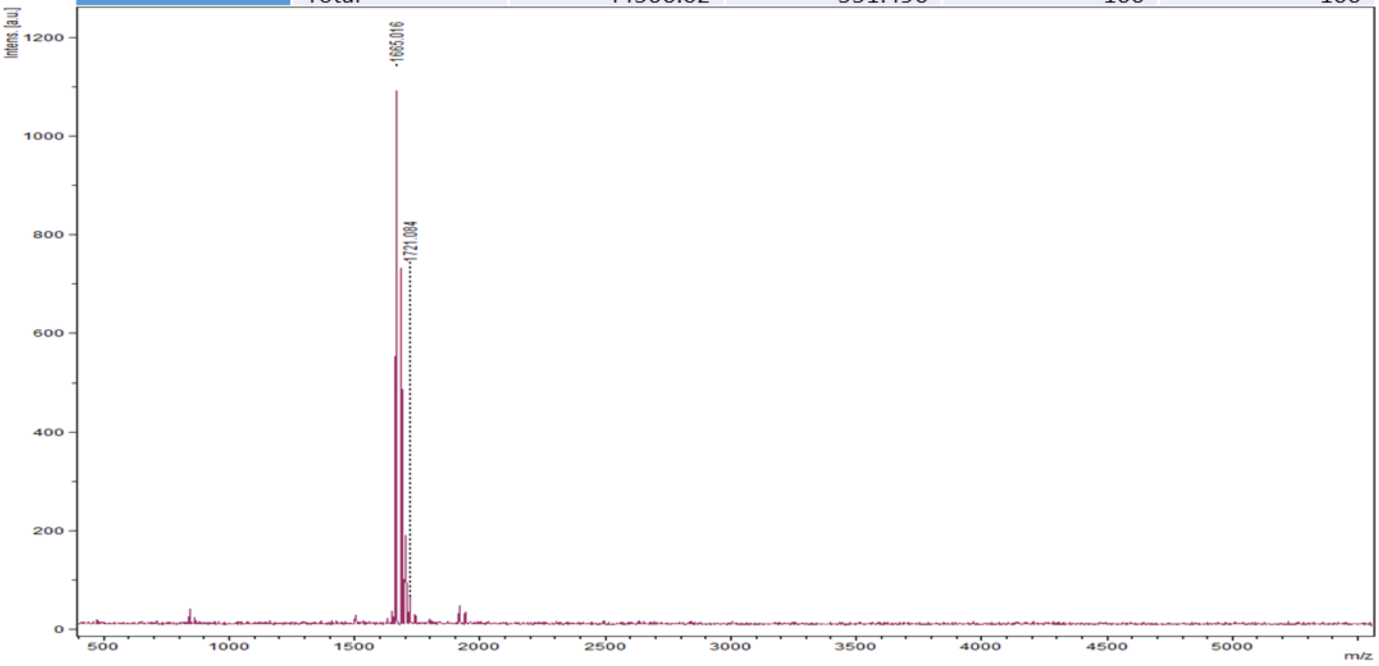


*Figure 5(C). Chromatogram and Mass Spectrum of KR12B*

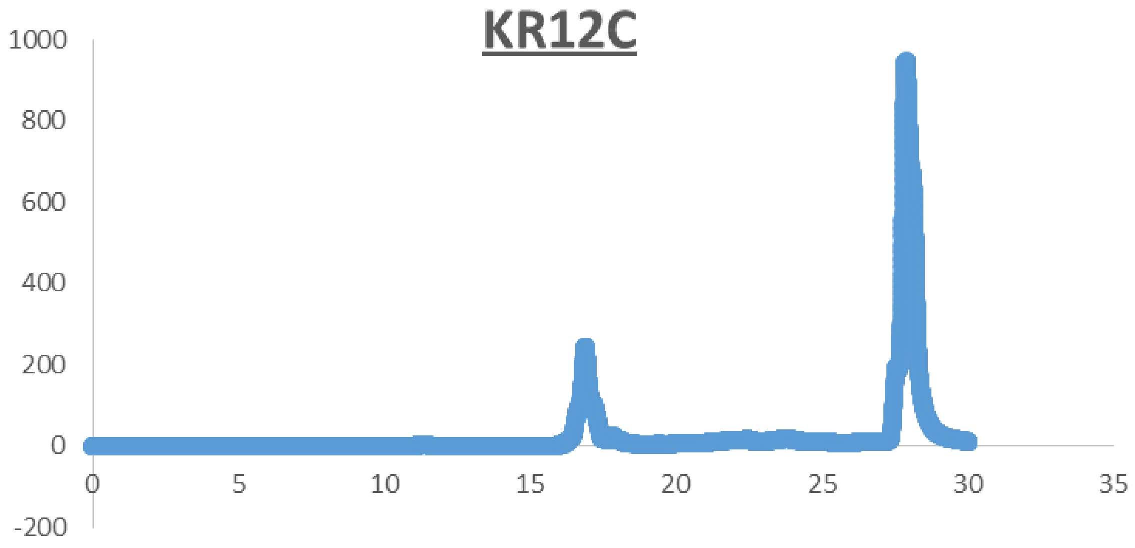


| Peak No. | Reten. time | Area [mV.s] | Height [mV] | Area [%] | Height [%] |
|---|---|---|---|---|---|
| | | | | | |
| 1 | 16.9 | 6973.235 | 243.264 | 19.321 | 20.611 |
| 2 | 27.897 | 29118.49 | 936.988 | 80.679 | 79.389 |
| | Total | 36091.73 | 1180.252 | 100 | 100 |

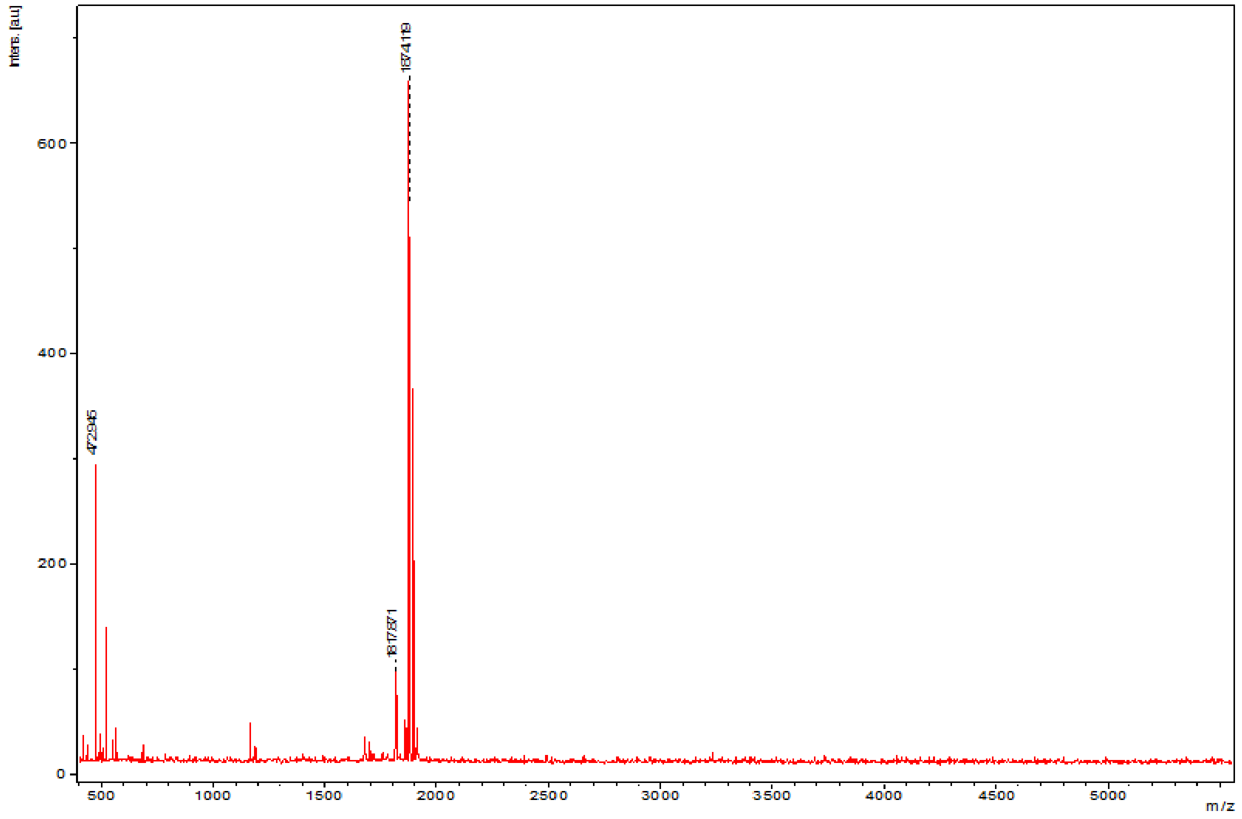


*Figure 5(D). Chromatogram and Mass Spectrum of KR12C*

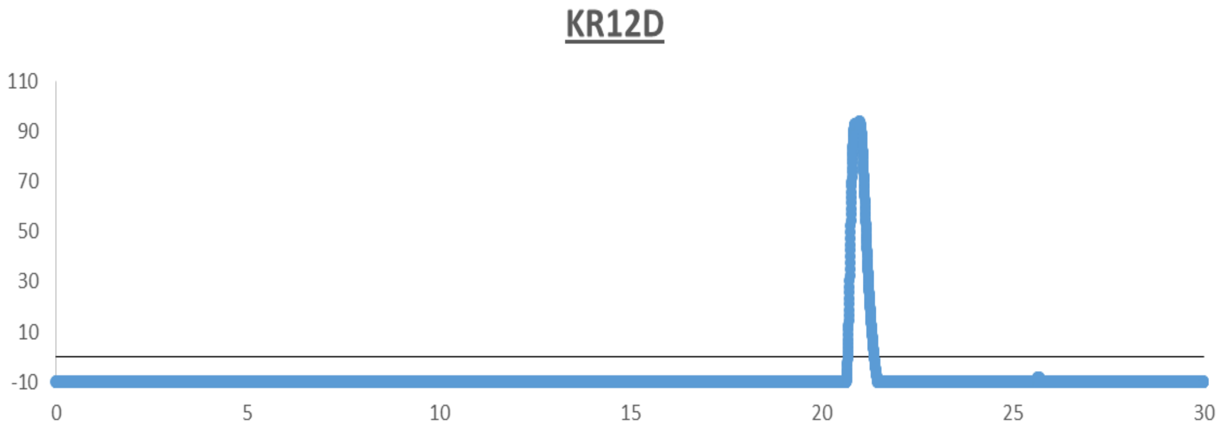


| Peak No. | Reten. time | Area [mV.s] | Height [mV] | Area [%] | Height [%] |
|---|---|---|---|---|---|
| 1 | 20.89 | 1057.488 | 102.25 | 37.364 | 49.408 |
| 2 | 21.003 | 1766.404 | 103.191 | 62.413 | 49.862 |
| 3 | 25.68 | 6.3123 | 1.511 | 0.223 | 0.73 |
| | Total | 2830.204 | 206.952 | 100 | 100 |

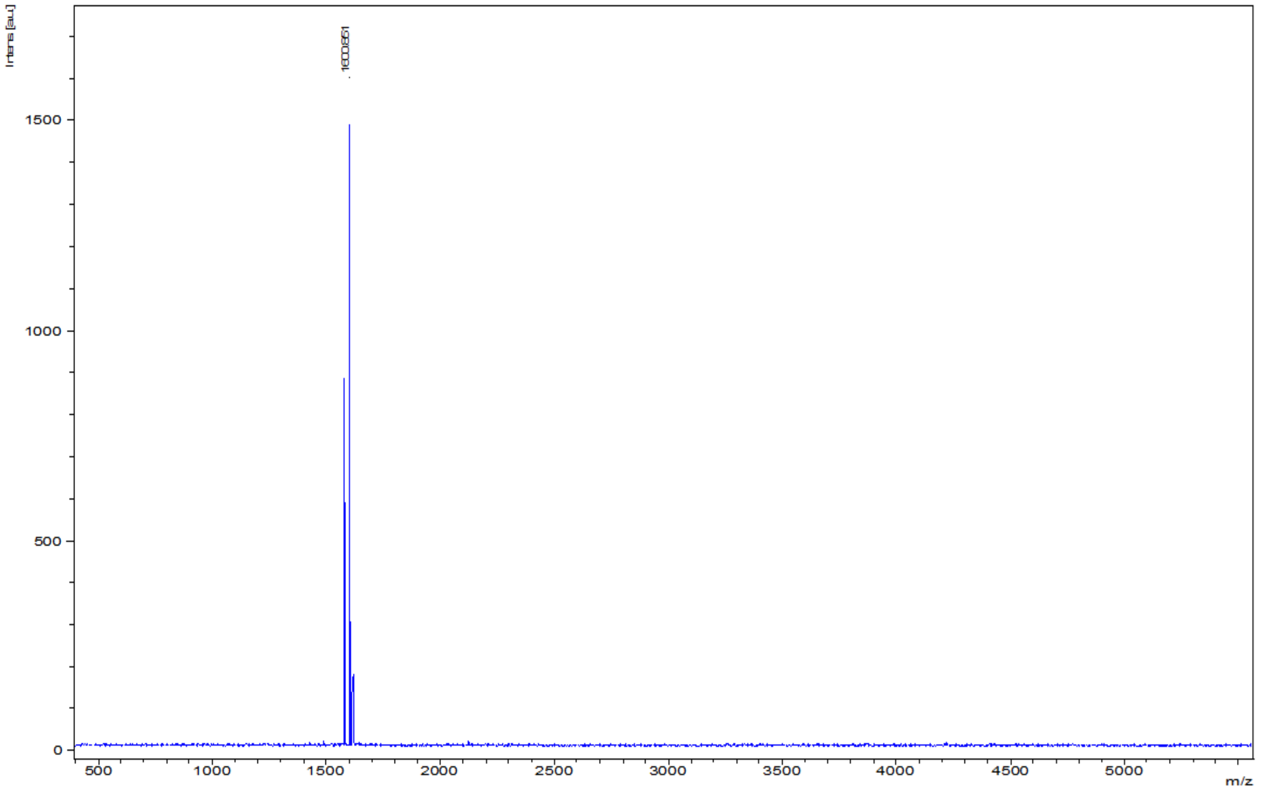


*Figure 5(E). Chromatogram and Mass Spectrum of KR12D*

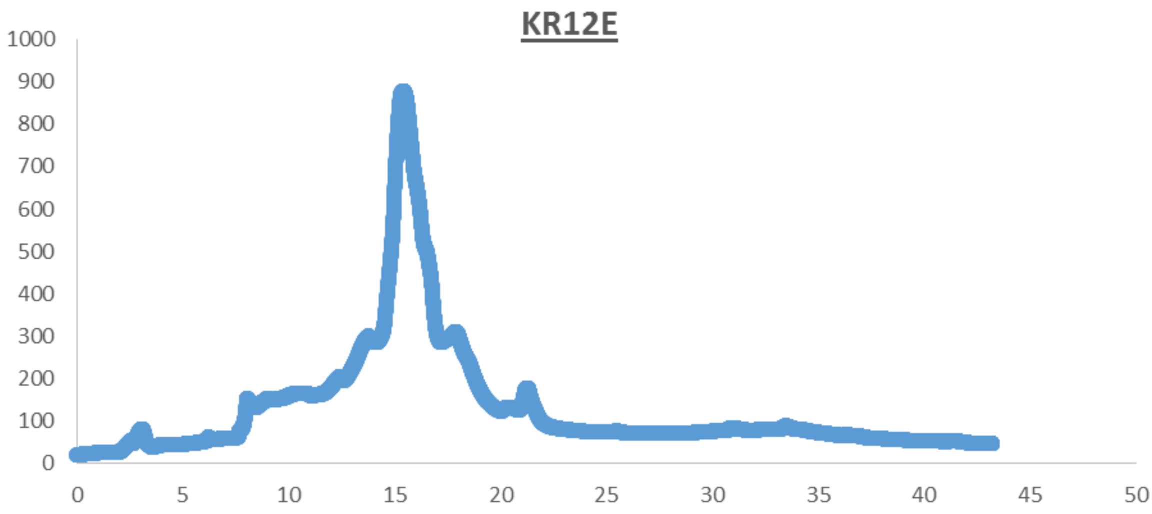


| Peak No. | Reten. time | Area [mV.s] | Height [mV] | Area [%] | Height [%] |
|---|---|---|---|---|---|
| | | | | | |
| 1 | 15.417 | 96942.93 | 848.942 | 86.015 | 85.389 |
| 2 | 21.25 | 15762.05 | 145.261 | 13.985 | 14.611 |
| | Total | 112705 | 994.203 | 100 | 100 |

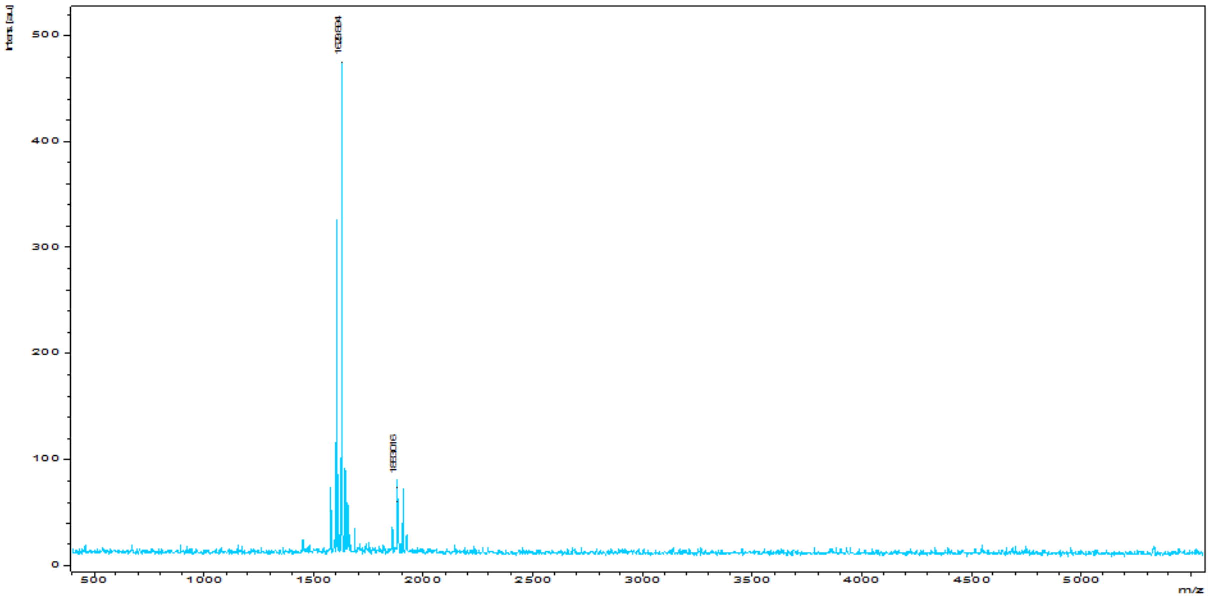


*Figure 5(F) Chromatogram and Mass Spectrum of KR12E*

**Figure S1**

| | Amino acid sequence | Net charge† | Permeability†† | Toxicity‡ | Anti-cancer† |
|---|---|---|---|---|---|
| LL37 | LLGDFFRKSKEKIGKEFKRIVQRIKDFLRNLVPRTES | +6 | - | - | + |
| Truncated peptides: FK13 | FKRIVQRIKDFLR | +4 | - | - | ++ |
| KR12A | KRIVQRIKKWLR | +6 | + | - | ++ |
| KR12B | KRIVKRIKKWLR | +7 | + | - | ++ |
| KR12C | KRIVKLIKKWLR | +6 | + | - | ++ |
| KR12D | KRIVKVIKKWLR | +6 | + | - | ++ |
| KR12E | KRIVKRIKKWLL | +5 | + | - | ++ |

† AntiCP prediction †† CellPPD prediction ‡ ToxinPred prediction

**Figure S2**

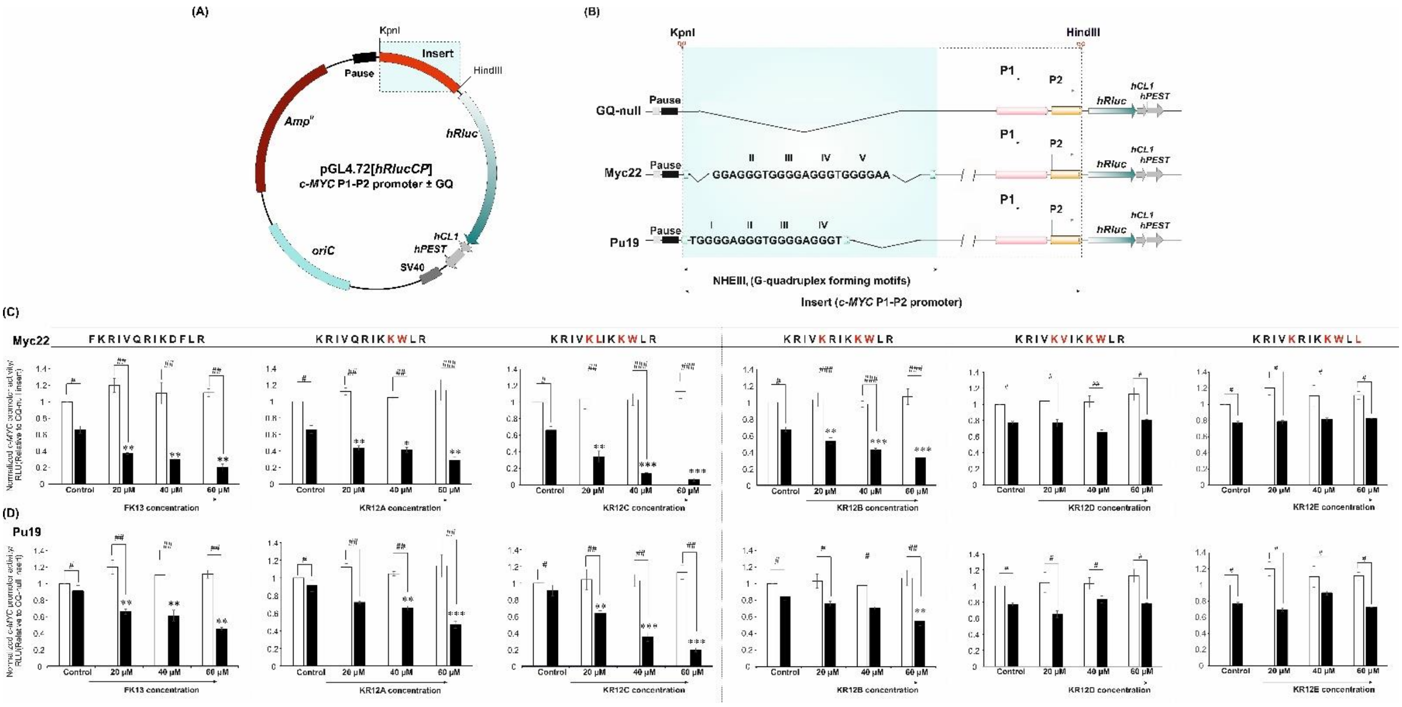

**Figure S3**

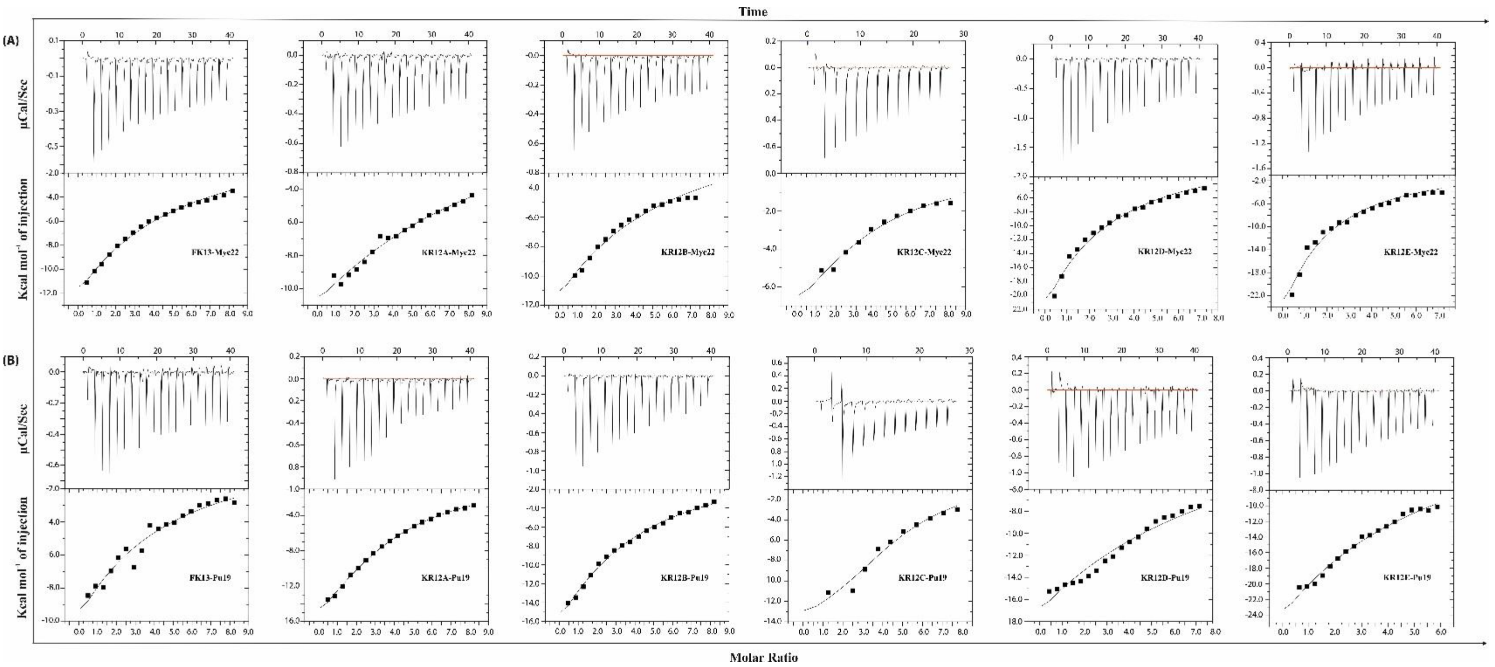


**Figure S4**

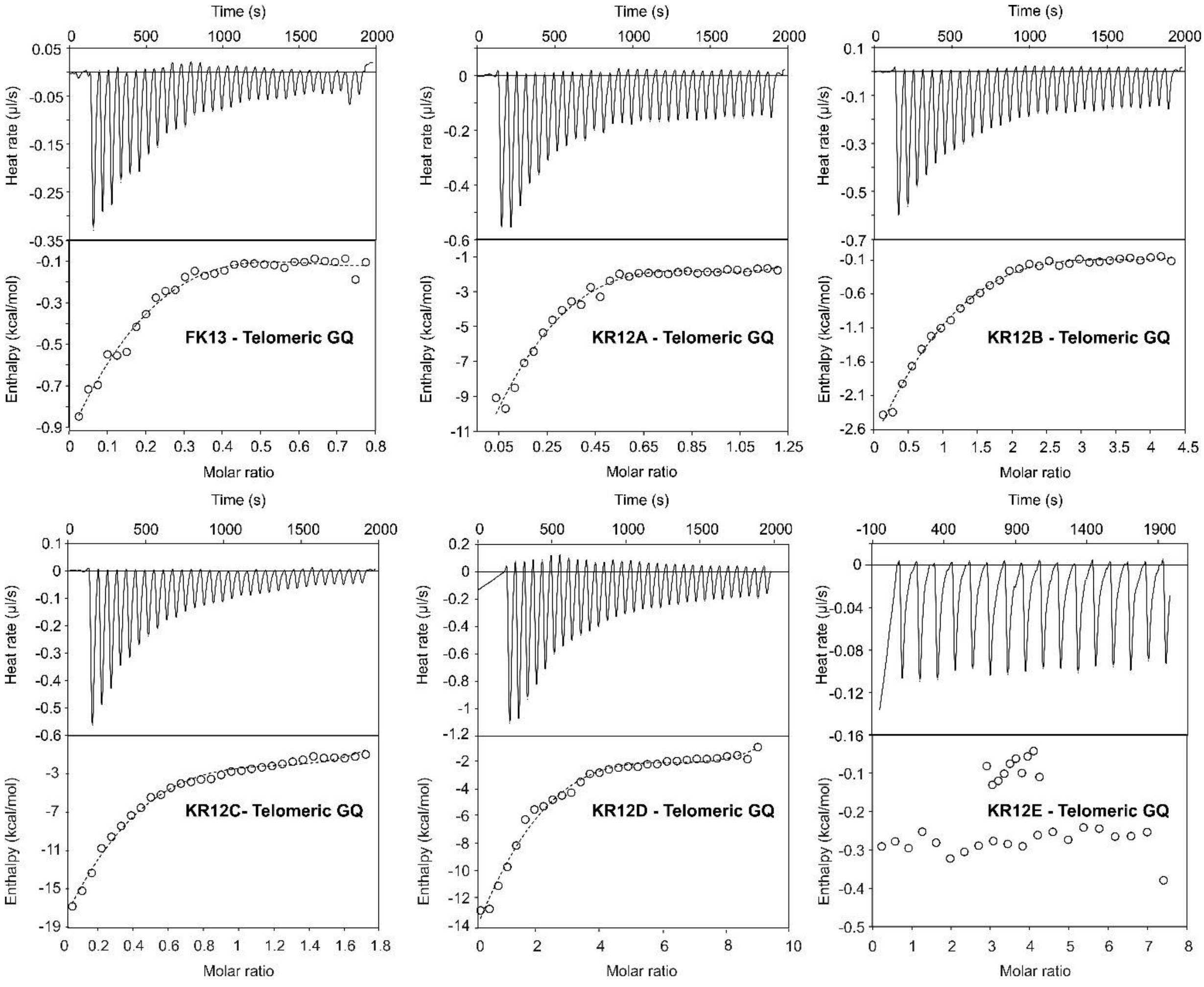

**Figure S5**

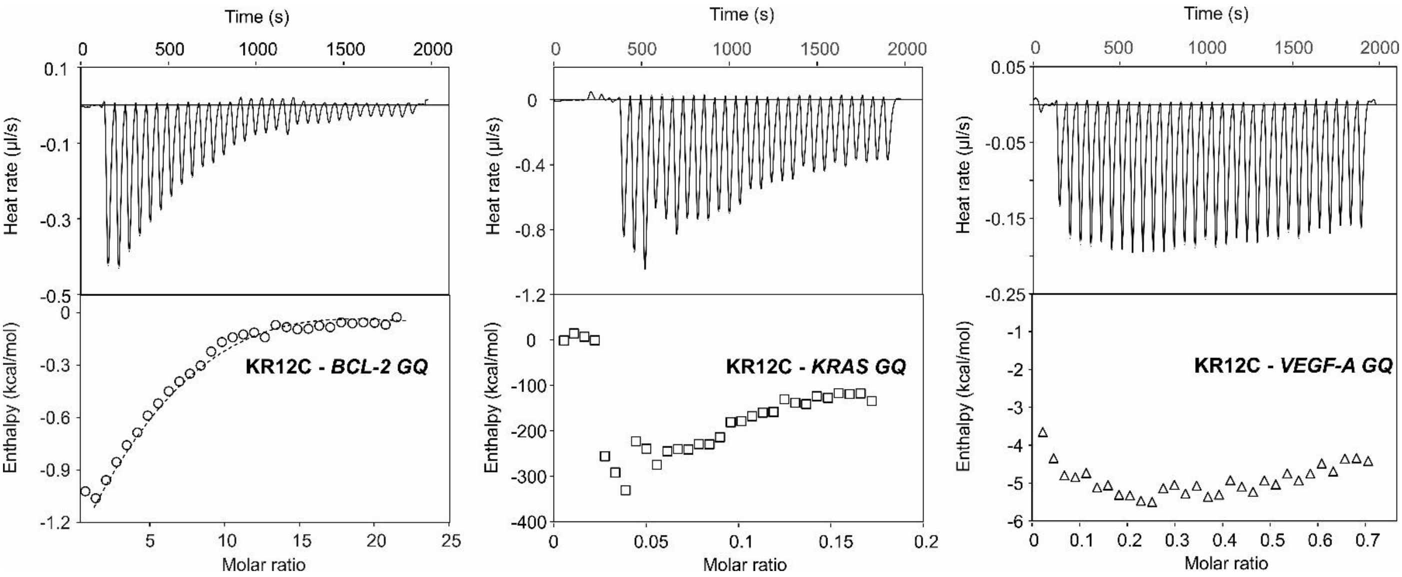


**Figure S6(A)**

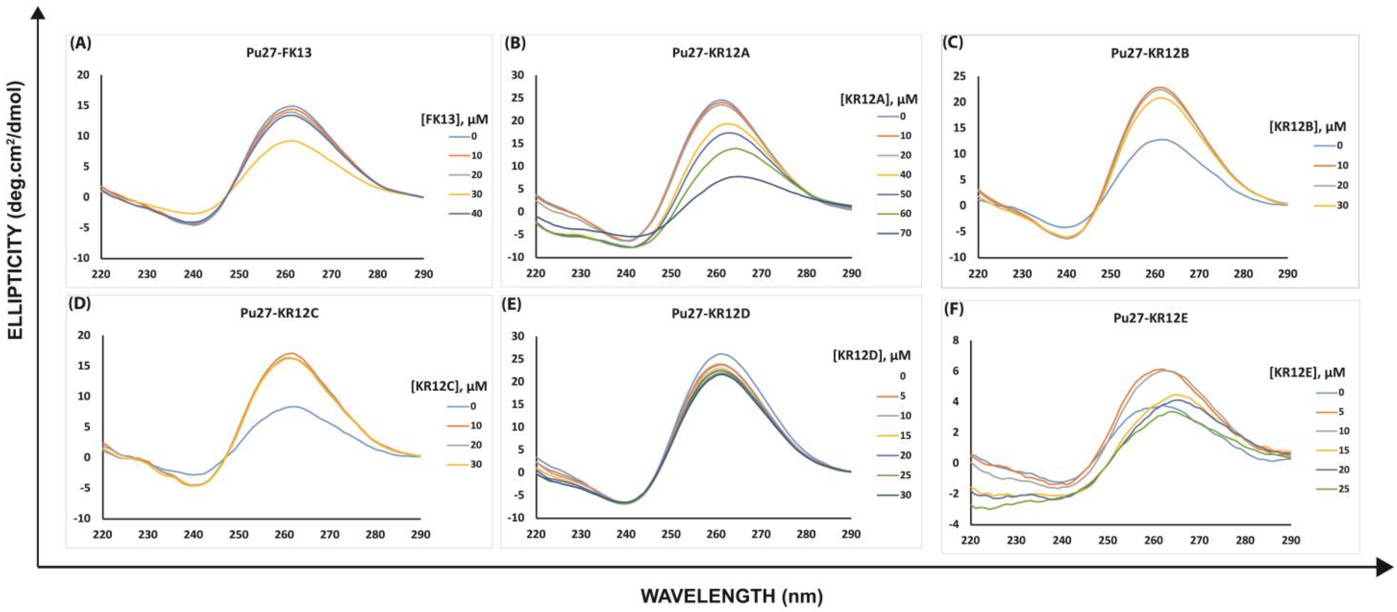

**Figure S6(B)**

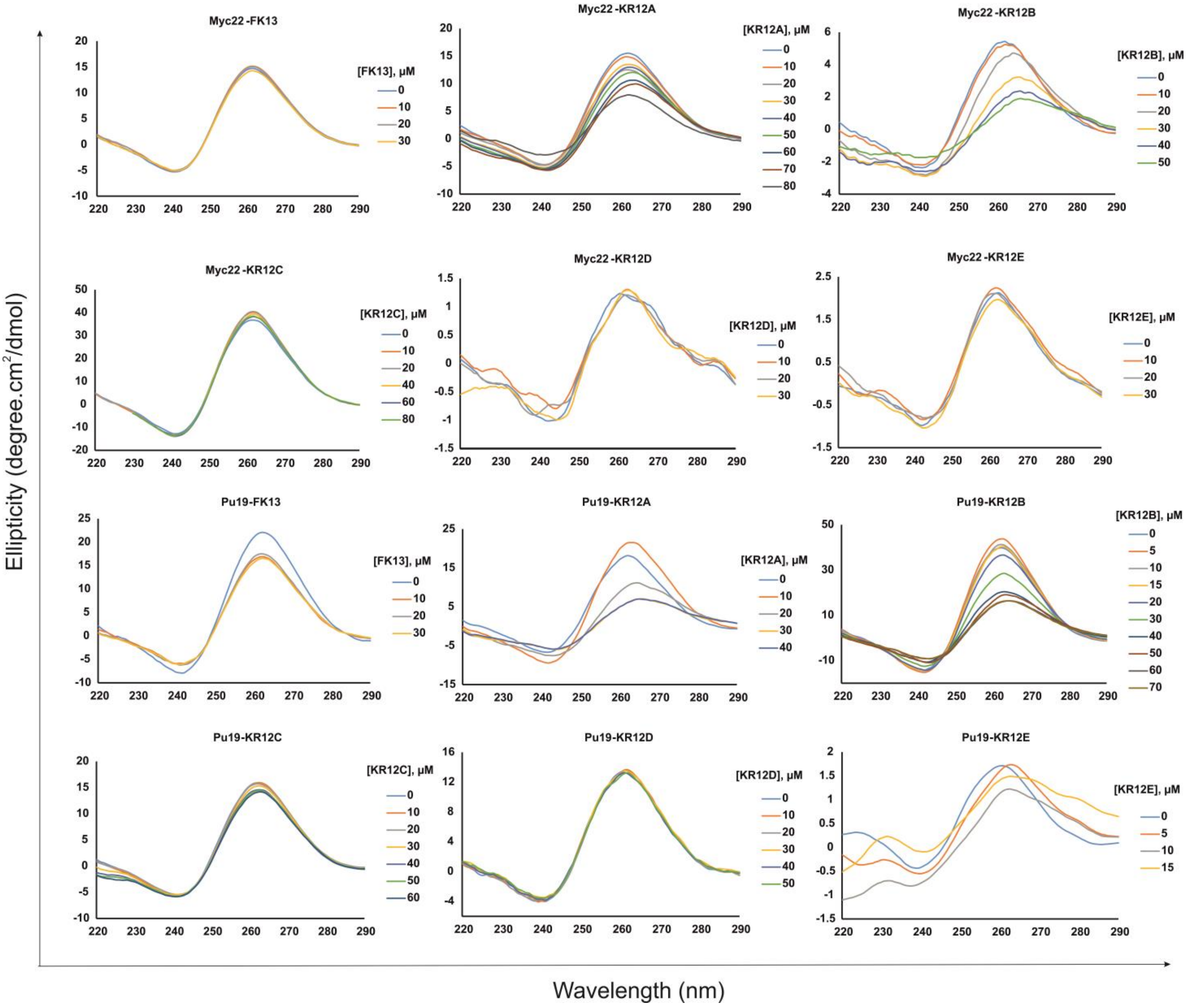
Myc22 -FK13
[FK13], μM
Myc22 -KR12A
[KR12A], μM
Myc22 -KR12B
[KR12B], μM
Myc22 -KR12C
[KR12C], μM
Myc22 -KR12D
[KR12D], μM
Myc22 -KR12E
[KR12E], μM
Pu19-FK13
[FK13], μM
Pu19-KR12A
[KR12A], μM
Pu19-KR12B
[KR12B], μM
Pu19-KR12C
[KR12C], μM
Pu19-KR12D
[KR12D], μM
Pu19-KR12E
[KR12E], μM
Ellipticity (degree.cm²/dmol)
Wavelength (nm)

**Figure S7**

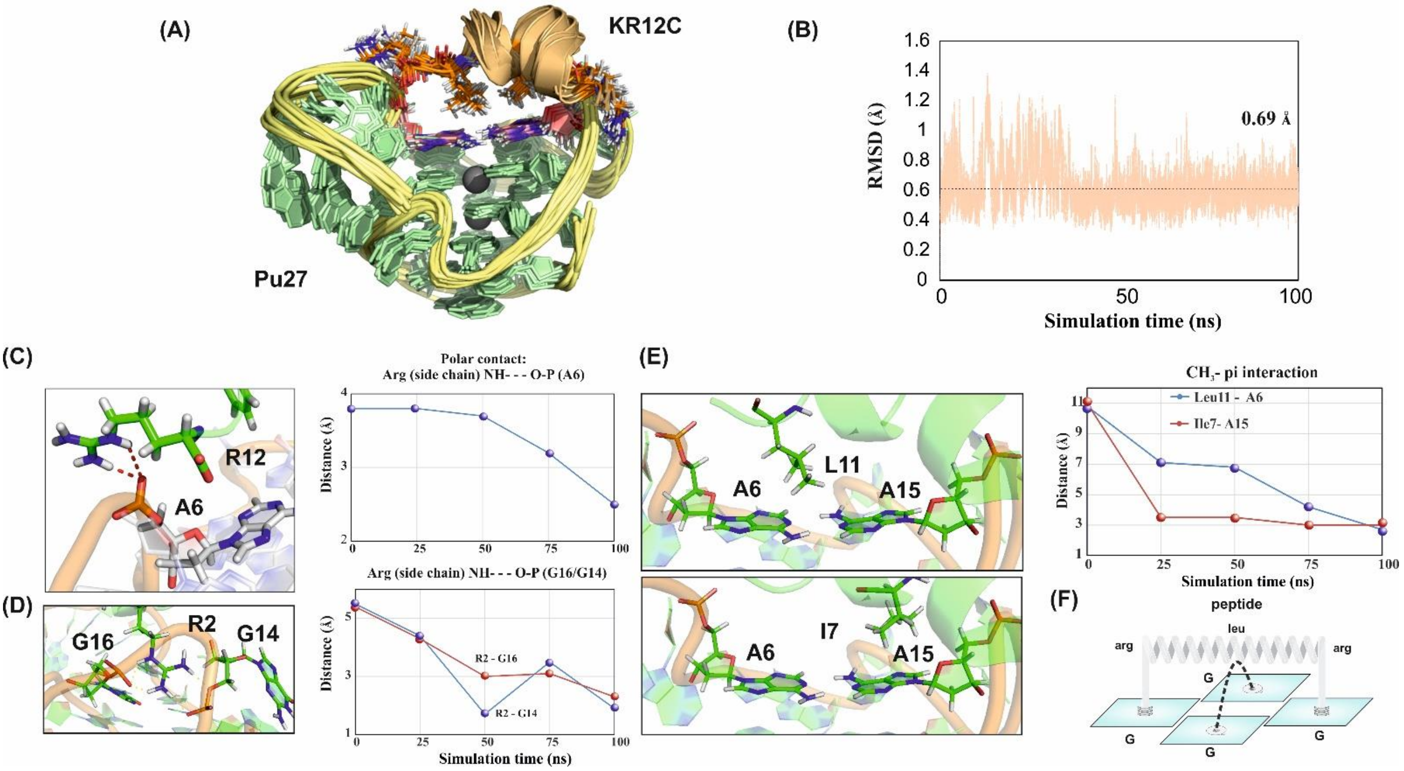

(A)
KR12C
Pu27
(B)
RMSD (Å)
0.69 Å
Simulation time (ns)
(C)
R12
A6
Polar contact:
Arg (side chain) NH- - - O-P (A6)
Distance (Å)
(D)
G16
R2
G14
Arg (side chain) NH- - - O-P (G16/G14)
R2 - G16
R2 - G14
Simulation time (ns)
(E)
A6
L11
A15
I7
CH3- pi interaction
Leu11 - A6
Ile7- A15
Simulation time (ns)
(F)
peptide
arg
leu
G

**Figure S8**

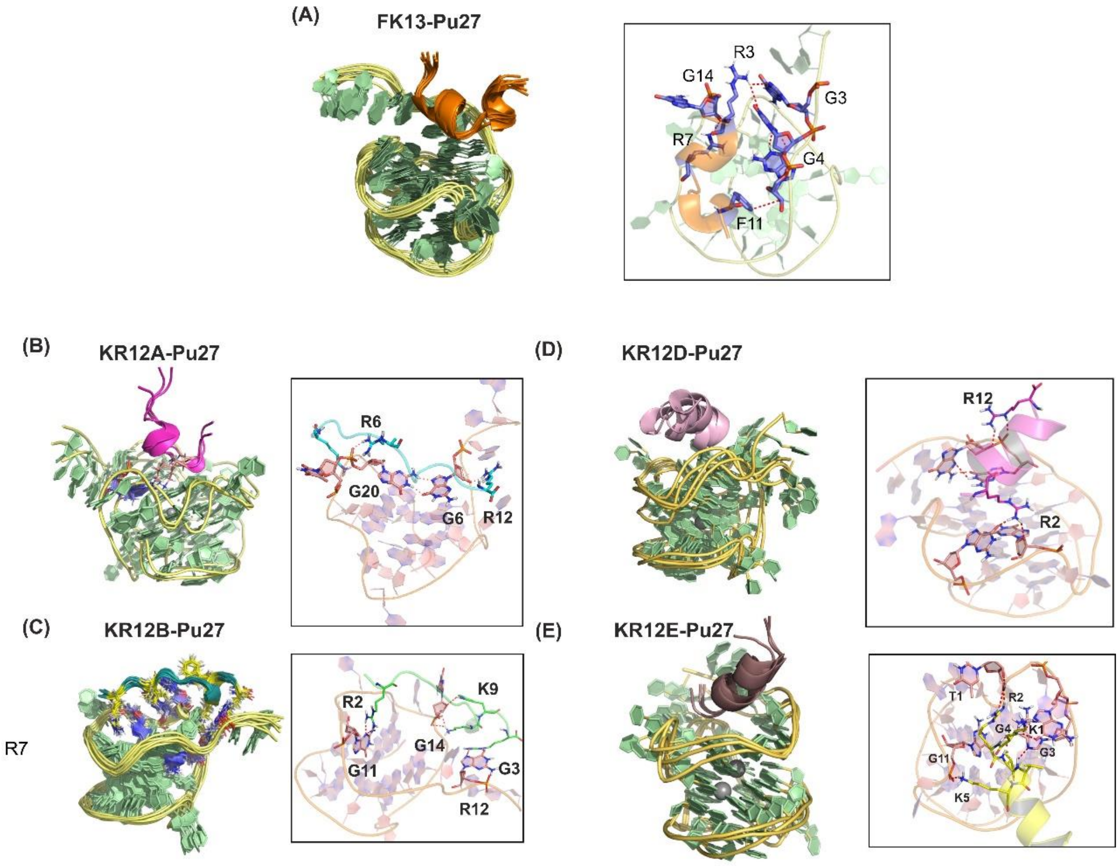
(A)
FK13-Pu27
R3
G14
G3
R7
G4
F11
(B)
KR12A-Pu27
R6
G20
G6
R12
(D)
KR12D-Pu27
R12
R2
(C)
KR12B-Pu27
R7
R2
K9
G14
G11
G3
R12
(E)
KR12E-Pu27
T1
R2
G4
K1
G3
G11
K5

**Figure S9**

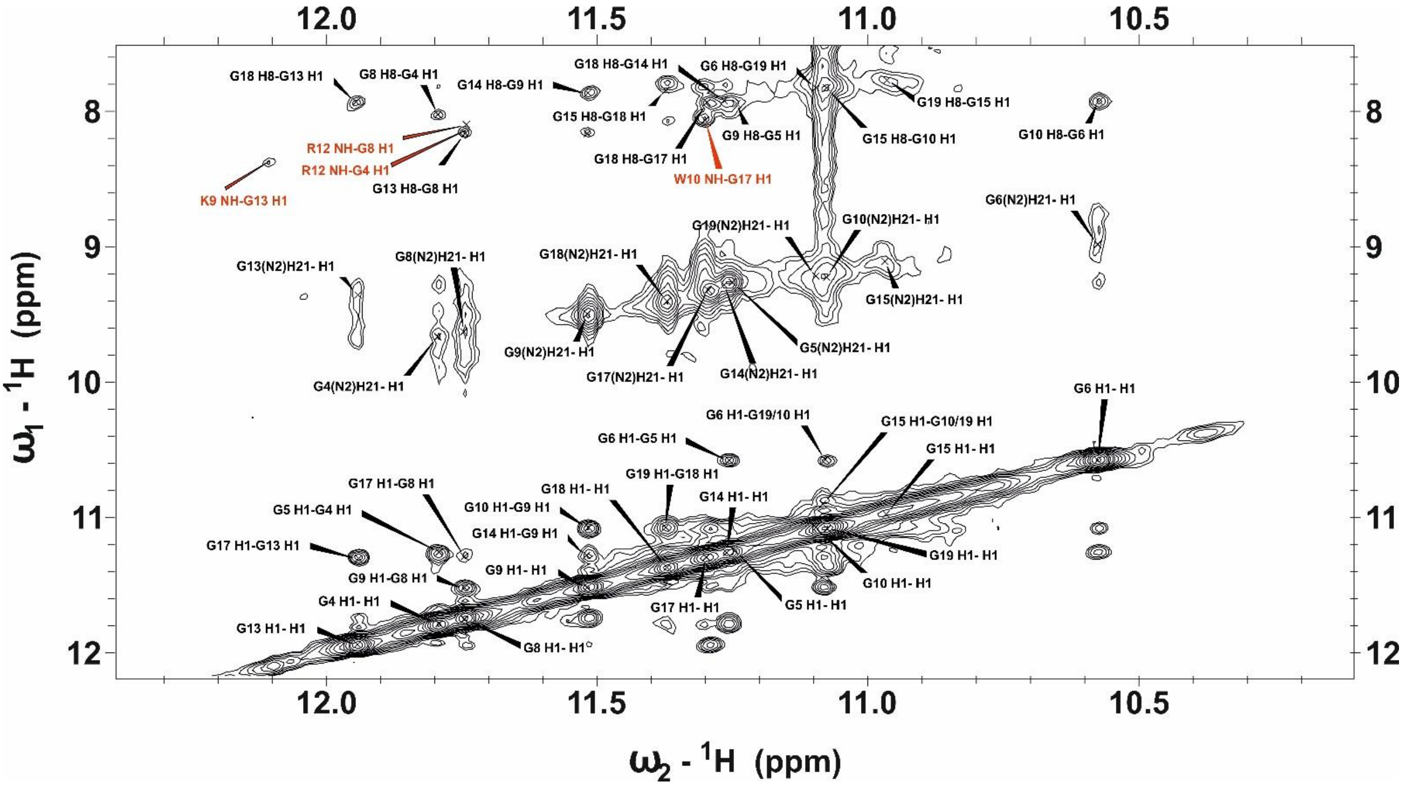


**Figure S10**

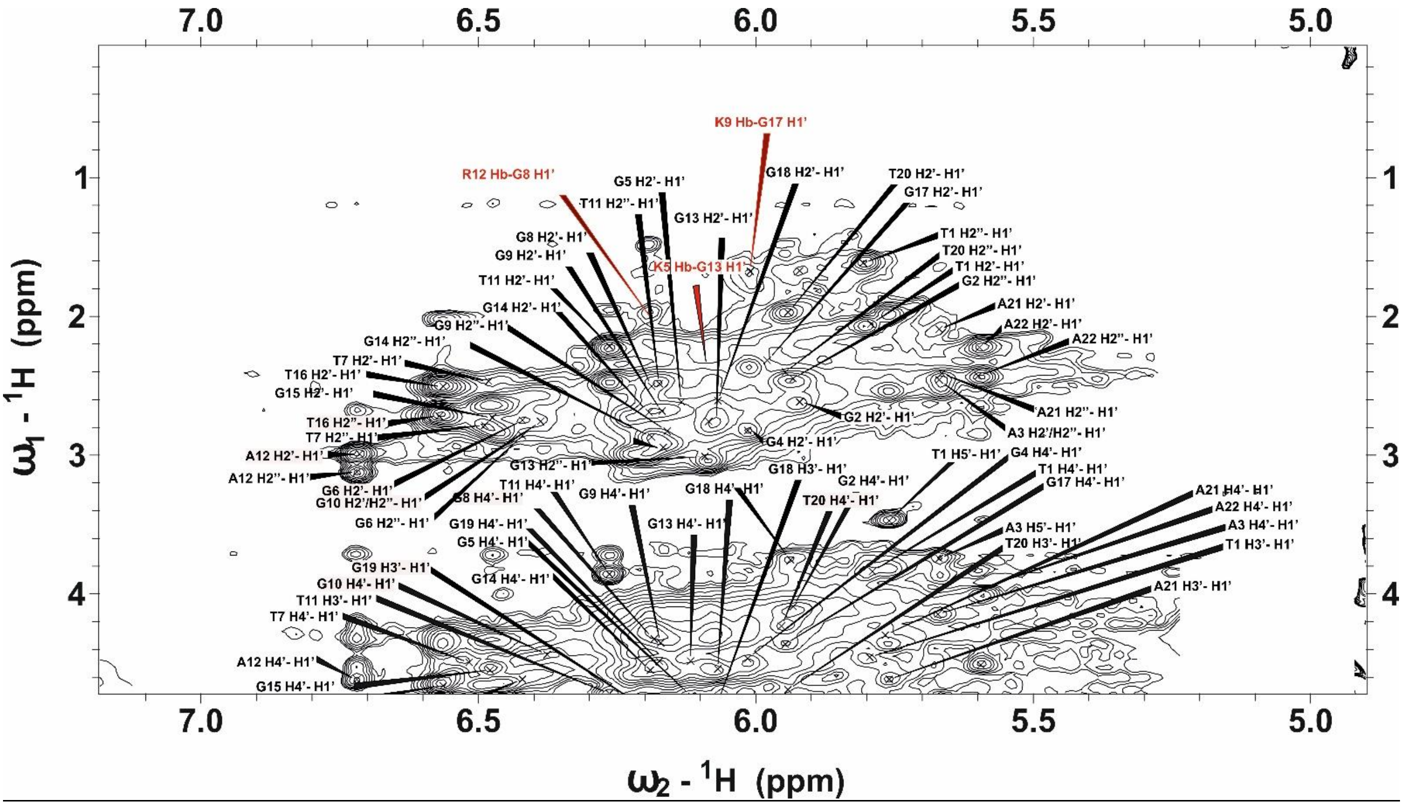

Figure S11

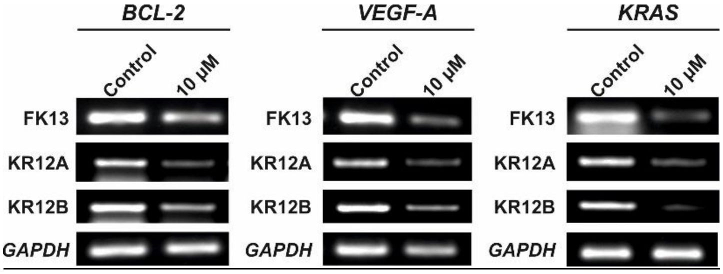


Figure S12

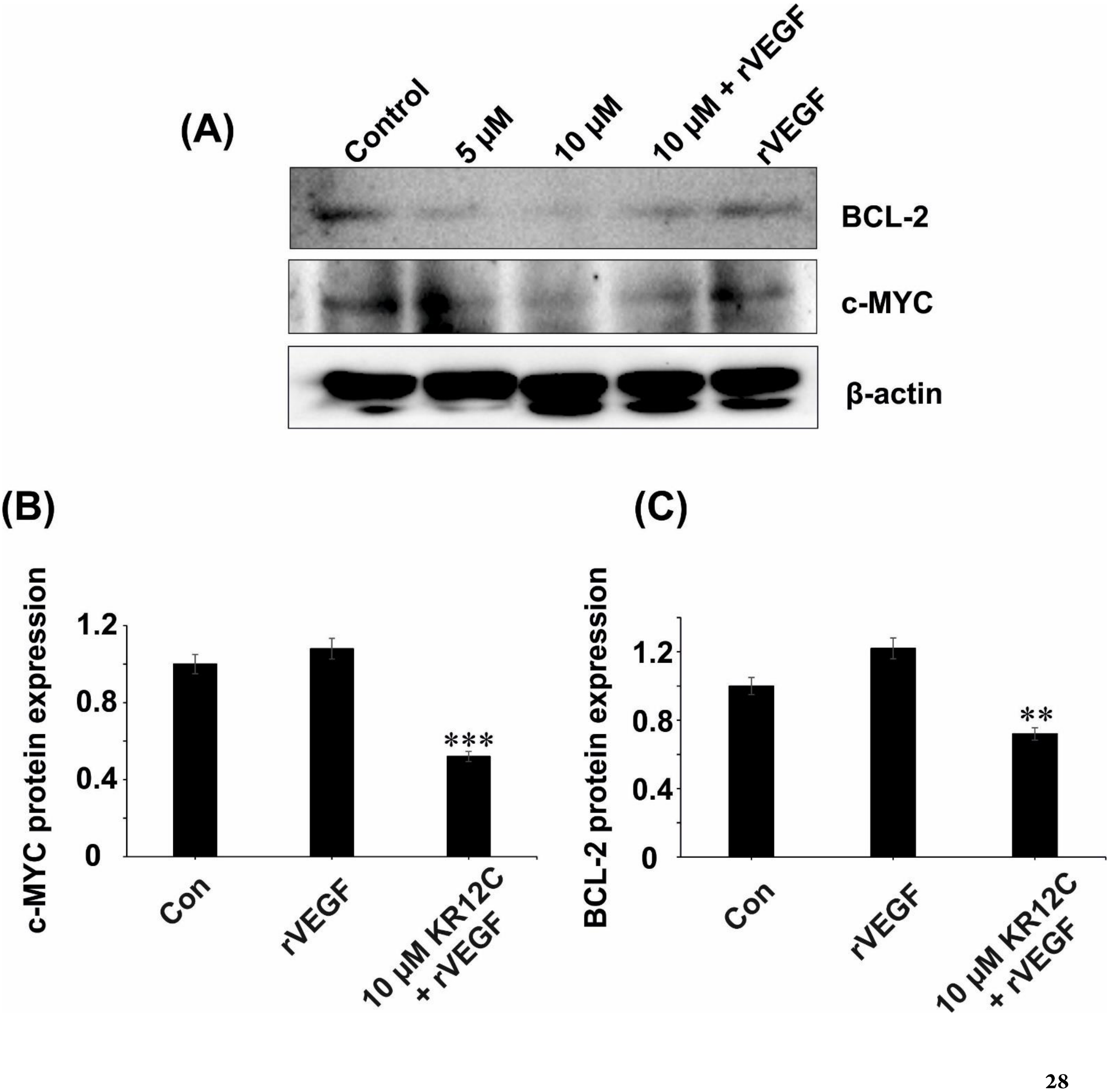

## 6. References:


1. Jana, J., Mondal, S., Bhattacharjee, P., Sengupta, P., Roychowdhury, T., Saha, P., Kundu, P. and Chatterjee, S. (2017) Chelerythrine down regulates expression of VEGFA, BCL2 and KRAS by arresting G-Quadruplex structures at their promoter regions. *Scientific reports*, 7, 40706.

2. Li, X., Zhao, X., Fang, Y., Jiang, X., Duong, T., Fan, C., Huang, C.C. and Kain, S.R. (1998) Generation of destabilized green fluorescent protein as a transcription reporter. *The Journal of biological chemistry*, **273**, 34970-34975.

3. Gilon, T., Chomsky, O. and Kulka, R.G. (1998) Degradation signals for ubiquitin system proteolysis in Saccharomyces cerevisiae. *The EMBO journal*, **17**, 2759-2766.

4. Han, Y., San-Marina, S., Liu, J. and Minden, M.D. (2004) Transcriptional activation of c-myc proto-oncogene by WT1 protein. *Oncogene*, **23**, 6933-6941.

5. Greenfield, N.J. (2006) Using circular dichroism spectra to estimate protein secondary structure. *Nature protocols*, **1**, 2876-2890.

6. Velazquez-Campoy, A. and Freire, E. (2006) Isothermal titration calorimetry to determine association constants for high-affinity ligands. *Nature protocols*, **1**, 186-191.

7. Krippahl, L. and Barahona, P. (2015) Protein docking with predicted constraints. *Algorithms for molecular biology : AMB*, **10**, 9.

8. Wang, J., Wolf, R.M., Caldwell, J.W., Kollman, P.A. and Case, D.A. (2004) Development and testing of a general amber force field. *Journal of computational chemistry*, **25**, 1157-1174.

9. Maier, J.A., Martinez, C., Kasavajhala, K., Wickstrom, L., Hauser, K.E. and Simmerling, C. (2015) ff14SB: Improving the Accuracy of Protein Side Chain and Backbone Parameters from ff99SB. *Journal of chemical theory and computation*, **11**, 3696-3713.

10. Price, D.J. and Brooks, C.L., 3rd. (2004) A modified TIP3P water potential for simulation with Ewald summation. *The Journal of chemical physics*, **121**, 10096-10103.

11. Roe, D.R. and Cheatham, T.E., 3rd. (2013) PTRAJ and CPPTRAJ: Software for Processing and Analysis of Molecular Dynamics Trajectory Data. *Journal of chemical theory and computation*, **9**, 3084-3095.

12. Humphrey, W., Dalke, A. and Schulten, K. (1996) VMD: visual molecular dynamics. *Journal of molecular graphics*, **14**, 33-38, 27-38.

13. Dai, J., Carver, M., Hurley, L.H. and Yang, D. (2011) Solution structure of a 2:1 quindoline-c-MYC G-quadruplex: insights into G-quadruplex-interactive small molecule drug design. *Journal of the American Chemical Society*, **133**, 17673-17680.
